# Changes in preterm birth and stillbirth during COVID-19 lockdowns in 26 countries

**DOI:** 10.1038/s41562-023-01522-y

**Published:** 2023-02-27

**Authors:** Clara Calvert, Meredith (Merilee) Brockway, Helga Zoega, Jessica E. Miller, Jasper V. Been, Adeladza Kofi Amegah, Amy Racine-Poon, Solmaz Eradat Oskoui, Ishaya I. Abok, Nima Aghaeepour, Christie D. Akwaowo, Belal N. Alshaikh, Adejumoke I. Ayede, Fabiana Bacchini, Behzad Barekatain, Rodrigo Barnes, Karolina Bebak, Anick Berard, Zulfiqar A. Bhutta, Jeffrey R. Brook, Lenroy R. Bryan, Kim N. Cajachagua-Torres, Marsha Campbell-Yeo, Dinh-Toi Chu, Kristin L. Connor, Luc Cornette, Sandra Cortés, Mandy Daly, Christian Debauche, Iyabode Olabisi F. Dedeke, Kristjana Einarsdóttir, Hilde Engjom, Guadalupe Estrada-Gutierrez, Ilaria Fantasia, Nicole M. Fiorentino, Meredith Franklin, Abigail Fraser, Onesmus W. Gachuno, Linda A. Gallo, Mika Gissler, Siri E. Håberg, Abbas Habibelahi, Jonas Häggström, Lauren Hookham, Lisa Hui, Luis Huicho, Karen J. Hunter, Sayeeda Huq, Ashish KC, Seilesh Kadambari, Roya Kelishadi, Narjes Khalili, Joanna Kippen, Kirsty Le Doare, Javier Llorca, Laura A. Magee, Maria C. Magnus, Kenneth K. C. Man, Patrick M. Mburugu, Rishi P. Mediratta, Andrew D. Morris, Nazeem Muhajarine, Rachel H. Mulholland, Livia Nagy Bonnard, Victoria Nakibuuka, Natasha Nassar, Sylvester D. Nyadanu, Laura Oakley, Adesina Oladokun, Oladapo O. Olayemi, Olanike A. Olutekunbi, Rosena O. Oluwafemi, Taofik O. Ogunkunle, Chris Orton, Anne K. Örtqvist, Joseph Ouma, Oyejoke Oyapero, Kirsten R. Palmer, Lars H. Pedersen, Gavin Pereira, Isabel Pereyra, Roy K. Philip, Dominik Pruski, Marcin Przybylski, Hugo G. Quezada-Pinedo, Annette K. Regan, Natasha R. Rhoda, Tonia A. Rihs, Taylor Riley, Thiago Augusto Hernandes Rocha, Daniel L. Rolnik, Christoph Saner, Francisco J. Schneuer, Vivienne L. Souter, Olof Stephansson, Shengzhi Sun, Emma M. Swift, Miklós Szabó, Marleen Temmerman, Lloyd Tooke, Marcelo L. Urquia, Peter von Dadelszen, Gregory A. Wellenius, Clare Whitehead, Ian C. K. Wong, Rachael Wood, Katarzyna Wróblewska-Seniuk, Kojo Yeboah-Antwi, Christopher S. Yilgwan, Agnieszka Zawiejska, Aziz Sheikh, Natalie Rodriguez, David Burgner, Sarah J. Stock, Meghan B. Azad

**Affiliations:** 1 Usher Institute, University of Edinburgh, Edinburgh, UK; 2 Children’s Hospital Research Institute of Manitoba, Department of Pediatrics and Child Health, University of Manitoba, Winnipeg, Manitoba Canada; 3 School of Population Health, Faculty of Medicine & Health, University of New South Wales Sydney, Sydney, New South Wales Australia; 4 Centre of Public Health Sciences, Faculty of Medicine, University of Iceland, Reykjavik, Iceland; 5 Murdoch Children’s Research Institute, Royal Children’s Hospital, Parkville, Victoria Australia; 6 Department of Paediatrics, University of Melbourne, Parkville, Victoria Australia; 7 Division of Neonatology, Department of Paediatrics; Department of Obstetrics and Gynaecology; Department of Public Health; Erasmus MC - Sophia Children’s Hospital, University Medical Centre Rotterdam, Rotterdam, the Netherlands; 8 Public Health Research Group, Department of Biomedical Sciences, University of Cape Coast, Cape Coast, Ghana; 9 Novartis, Basel, Switzerland; 10 Department of Pediatrics, University of Jos/Jos University Teaching Hospital, Jos, Nigeria; 11 Department of Anesthesiology, Pain, and Perioperative Medicine, Stanford University School of Medicine, Stanford, CA USA; 12 Institute of Health Research and Development, University of Uyo Teaching Hospital, Uyo, Nigeria; 13 College of Health Sciences, University of Uyo, Uyo, Nigeria; 14 Department of Pediatrics, University of Calgary, Calgary, Alberta Canada; 15 Department of Pediatrics, College of Medicine, University of Ibadan and University College Hospital, Ibadan, Nigeria; 16 Canadian Premature Babies Foundation, Toronto, Ontario Canada; 17 Department of Pediatrics, Division of Neonatology, Child Growth and Development Research Center, Isfahan University of Medical Sciences, Isfahan, Iran; 18 Aridhia Informatics, Glasgow, UK; 19 Obstetrics and Gynaecology Ward, District Public Hospital in Poznań, Poznań, Poland; 20 Faculty of Pharmacy, University of Montreal, Montreal, Quebec Canada; 21 CHU Ste-Justine, Montreal, Quebec Canada; 22 Faculty of Medicine, Université Claude Bernard Lyon 1, Lyon, France; 23 Center of Excellence in Women Child Health, The Aga Khan University, Karachi, Pakistan; 24 Centre for Global Child Health, Hospital for Sick Children, Toronto, Ontario Canada; 25 Dalla Lana School of Public Health, University of Toronto, Toronto, Ontario Canada; 26 Department of Obstetrics & Gynaecology and Child Health, University of The West MonaIndies, Mona, Jamaica; 27 The Generation R Study Group, Erasmus MC, University Medical Center Rotterdam, Rotterdam, The Netherlands; 28 The Department of Paediatrics, Erasmus MC, University Medical Center Rotterdam, Rotterdam, The Netherlands; 29 Centro de Investigación en Salud Materna e Infantil and Centro de Investigación para el Desarrollo Integral y Sostenible, Universidad Peruana Cayetano Heredia, Lima, Peru; 30 School of Nursing, Dalhousie University and IWK Health, Halifax, Nova Scotia Canada; 31 Center for Biomedicine and Community Health, International School, Vietnam National University, Hanoi, Vietnam; 32 Department of Health Sciences, Carleton University, Ottawa, Ontario Canada; 33 AZ St-Jan Bruges-Ostend AV Hospital, Bruges, Belgium; 34 Department of Public Health, School of Medicine, Advanced Center for Chronic Diseases Diagonal (ACCDIS), Santiago, Chile; 35 Irish Neonatal Health Alliance, Wicklow, Ireland; 36 Department of Neonatology, Cliniques Universitaires Saint-Luc, IREC, UCLouvain, Brussels, Belgium; 37 CEpiP (Centre d’Epidémiologie Périnatale), Brussels, Belgium; 38 Department of Paediatrics, Federal Medical Centre, Abeokuta, Nigeria; 39 Curtin School of Population Health, Curtin University, Perth, Western Australia Australia; 40 Department of Health Registry Research and Development, Norwegian Institute of Public Health, Oslo, Norway; 41 National Institute of Perinatology, Mexico City, Mexico; 42 Institute for Maternal and Child Health, IRCCS Burlo Garofolo Children’s Hospital, Trieste, Italy; 43 Department of Statistical Sciences and School of the Environment, University of Toronto, Toronto, Ontario Canada; 44 Population Health Sciences, Bristol Medical School, University of Bristol, Bristol, UK; 45 Obstetrics and Gynecology, Medicine, University of Nairobi, Nairobi, Kenya; 46 School of Biomedical Sciences, The University of Queensland, St. Lucia, Queensland Australia; 47 Department of Knowledge Brokers, THL Finnish Institute for Health and Welfare, Helsinki, Finland; 48 Academic Primary Health Care Centre, Region Stockholm, Stockholm, Sweden; 49 Department of Molecular Medicine and Surgery, Karolinska Institutet, Stockholm, Sweden; 50 Centre for Fertility and Health, Norwegian Institute of Public Health, Oslo, Norway; 51 Neonatology, Neonatal Health Office, Ministry of Health and Medical Education, Tehran, Iran; 52 Cytel, Waltham, MA USA; 53 St. George’s University, Makerere University - Johns Hopkins University Research Collaboration, London, UK; 54 Department of Obstetrics and Gynaecology, University of Melbourne, Melbourne, Victoria Australia; 55 Centro de Investigación en Salud Materna e Infantil, Centro de Investigación para el Desarrollo Integral y Sostenible and School of Medicine, Universidad Peruana Cayetano Heredia, Lima, Peru; 56 Nutrition and Clinical Services Division, ICDDR,B (International Centre for Diarrhoeal Disease Research, Bangladesh), Dhaka, Bangladesh; 57 Uppsala University, Uppsala, Sweden; 58 Oxford Vaccine Group, Department of Paediatrics, University of Oxford and the NIHR Oxford Biomedical Research Centre, Oxford, UK; 59 Child Growth and Development Research Center, Research Institute for Primordial Prevention of Non-Communicable Disease, Isfahan University of Medical Sciences, Isfahan, Iran; 60 Preventive Medicine and Public Health Research Center, Psychosocial Health Research Institute, Department of Community and Family Medicine, School of Medicine, Iran University of Medical Sciences, Tehran, Iran; 61 International Centre for Neonatal and Paediatric Infection, St. George’s, University of London, London, UK; 62 Medical Research Council/Uganda Virus Research Institute and London School of Medical Hygiene & Tropical Medicine Uganda Research Unit, Entebbe, Uganda; 63 Universidad de Cantabria, Santander, Spain; 64 CIBERESP (Consortium for Biomedical Research in Epidemiology & Public Health, en Epidemiología y Salud Pública), Madrid, Spain; 65 Institute of Women and Children’s Health, School of Life Course and Population Sciences, King’s College London, London, UK; 66 Research Department of Practice and Policy, University College London School of Pharmacy, London, UK; 67 Department of Pharmacology and Pharmacy, Li Ka Shing Faculty of Medicine, The University of Hong Kong, Hong Kong, Hong Kong; 68 Laboratory of Data Discovery for Health, Hong Kong Science Park, Hong Kong, Hong Kong; 69 Department of Child Health and Paediatrics, School of Medicine, Jomo Kenyatta University of Agriculture and Technology, Nairobi, Kenya; 70 Division of Pediatric Hospital Medicine, Department of Pediatrics, Stanford University School of Medicine, Stanford, CA USA; 71 Health Data Research UK, London, UK; 72 Community Health and Epidemiology, College of Medicine, University of Saskatchewan, Saskatoon, Saskatchewan Canada; 73 Melletted a helyem Egyesület, Right(s) Beside You Association, Budapest, Hungary; 74 Department of Paediatrics, St. Francis Nsambya Hospital, Kampala, Uganda; 75 Child Population and Translational Health Research, Children’s Hospital at Westmead Clinical School, Faculty of Medicine and Health, University of Sydney, Sydney, New South Wales Australia; 76 Education, Culture, and Health Opportunities (ECHO) Research Group International, Aflao, Ghana; 77 Faculty of Epidemiology and Population Health, London School of Hygiene and Tropical Medicine, London, UK; 78 Department of Obstetrics and Gynaecology, College of Medicine, University of Ibadan and University College Hospital, Ibadan, Nigeria; 79 Lagos Island Maternity Hospital, Lagos, Nigeria; 80 Department of Paediatrics and Child Health, Mother and Child Hospital, Akure, Nigeria; 81 Department of Paediatrics, Dalhatu Araf Specialist Hospital, Lafia, Nigeria; 82 Swansea University, Swansea, UK; 83 Clinical Epidemiology Division, Department of Medicine, Solna, Karolinska Institutet, Stockholm, Sweden; 84 Department of Obstetrics and Gynecology, Visby County Hospital, Visby, Sweden; 85 Makerere University - Johns Hopkins University Research Collaboration, Kampala, Uganda; 86 Paediatrics Department, Ikorodu General Hospital, Ikorodu, Nigeria; 87 Department of Obstetrics and Gynaecology, Monash University, Clayton, Victoria Australia; 88 Department of Obstetrics and Gynecology, Aarhus University and Aarhus University Hospital, Aarhus, Denmark; 89 Curtin School of Population Health and enAble Institute, Curtin University, Perth, Western Australia Australia; 90 School of Nutrition, Catholic University del Maule, Region del Maule, Chile; 91 Division of Neonatology, Department of Paediatrics, University Maternity Hospital Limerick and University of Limerick School of Medicine, Limerick, Ireland; 92 School of Nursing and Health Professions, University of San Francisco, San Francisco, CA USA; 93 Paediatric Department, School of Adolescent and Child Health, University of Cape Town, Cape Town, South Africa; 94 Mowbray Maternity Hospital, Western Cape Department of Health, Cape Town, South Africa; 95 Federal Statistical Office (FSO), Neuchâtel, Switzerland; 96 Department of Epidemiology, University of Washington, Seattle, WA USA; 97 Evidence and Intelligence for Action in Health Department, Pan-American Health Organization - World Health Organization, Washington, DC USA; 98 Division of Pediatric Endocrinology, Diabetology and Metabolism, Department of Pediatrics, Inselspital, Bern University Hospital, University of Bern, Bern, Switzerland; 99 Department of Biomedical Research, University of Bern, Bern, Switzerland; 100 The Children’s Hospital at Westmead Clinical School, Faculty of Medicine and Health, The University of Sydney, Sydney, New South Wales Australia; 101 Foundation for Health Care Quality, Seattle, WA USA; 102 Department of Environmental Health, Boston University School of Public Health, Boston, MA USA; 103 Faculty of Nursing, Department of Midwifery, University of Iceland, Reykjavík, Iceland; 104 Division of Neonatology, 1st Department of Pediatrics, Semmelweis University, Budapest, Hungary; 105 Centre of Excellence in Women and Child Health, Aga Khan University, Nairobi, Kenya; 106 Department of Paediatrics, University of Cape Town, Cape Town, South Africa; 107 Manitoba Centre for Health Policy, Department of Community Health Sciences, Rady Faculty of Health Sciences, University of Manitoba, Winnipeg, Manitoba Canada; 108 The Royal Women’s Hospital, University of Melbourne, Melbourne, Victoria Australia; 109 Public Health Scotland, Edinburgh, UK; 110 II Department of Neonatology, Poznań University of Medical Sciences, Poznań, Poland; 111 Public Health Unit, Father Thomas Alan Rooney Memorial Hospital, Asankrangwa, Western Region, Ghana; 112 Department of Paediatrics, University of Jos, Jos, Nigeria; 113 Department of Medical Simulation, Chair of Medical Education, Poznań University of Medical Sciences, Poznań, Poland; 114 Departments of Pediatrics and Child Health, Community Health Sciences, and Immunology, University of Manitoba, Winnipeg, Manitoba, Canada, Winnipeg, Manitoba Canada

**Keywords:** Outcomes research, Epidemiology

## Abstract

Preterm birth (PTB) is the leading cause of infant mortality worldwide. Changes in PTB rates, ranging from −90% to +30%, were reported in many countries following early COVID-19 pandemic response measures (‘lockdowns’). It is unclear whether this variation reflects real differences in lockdown impacts, or perhaps differences in stillbirth rates and/or study designs. Here we present interrupted time series and meta-analyses using harmonized data from 52 million births in 26 countries, 18 of which had representative population-based data, with overall PTB rates ranging from 6% to 12% and stillbirth ranging from 2.5 to 10.5 per 1,000 births. We show small reductions in PTB in the first (odds ratio 0.96, 95% confidence interval 0.95–0.98, *P* value <0.0001), second (0.96, 0.92–0.99, 0.03) and third (0.97, 0.94–1.00, 0.09) months of lockdown, but not in the fourth month of lockdown (0.99, 0.96–1.01, 0.34), although there were some between-country differences after the first month. For high-income countries in this study, we did not observe an association between lockdown and stillbirths in the second (1.00, 0.88–1.14, 0.98), third (0.99, 0.88–1.12, 0.89) and fourth (1.01, 0.87–1.18, 0.86) months of lockdown, although we have imprecise estimates due to stillbirths being a relatively rare event. We did, however, find evidence of increased risk of stillbirth in the first month of lockdown in high-income countries (1.14, 1.02–1.29, 0.02) and, in Brazil, we found evidence for an association between lockdown and stillbirth in the second (1.09, 1.03–1.15, 0.002), third (1.10, 1.03–1.17, 0.003) and fourth (1.12, 1.05–1.19, <0.001) months of lockdown. With an estimated 14.8 million PTB annually worldwide, the modest reductions observed during early pandemic lockdowns translate into large numbers of PTB averted globally and warrant further research into causal pathways.

## Abstract (web summary)

An analysis of 52 million births in 26 countries shows small reductions in preterm birth during the first to third months of lockdown. Further research is needed to examine causal pathways.

## Main

Approximately 10% of babies are born preterm (that is, before 37 completed weeks gestation), corresponding to nearly 15 million preterm births annually^1^. Preterm birth and related complications are the leading cause of infant mortality, and those who survive face an increased risk of morbidity and mortality across the life course^2^. While most preterm births are spontaneous, some are planned to reduce the risk of adverse outcomes including stillbirth, which account for two million in utero deaths globally each year^3,4^. A decline in preterm birth rates can therefore be an indicator that high-risk women and their babies are not receiving timely, quality care, potentially leading to increases in stillbirths.

In the first few months following the introduction of pandemic-related restrictions (henceforth referred to as ‘lockdowns’) in response to the first wave of coronavirus disease 2019 (COVID-19), there were markedly varying reports of changes in preterm birth and stillbirth rates across countries. Substantial reductions in preterm birth were reported from a number of high-income countries (HICs), including Australia (29–36%) (refs. ^5,6^), Israel (40%) (ref. ^7^) and some European countries (16–91%) (refs. ^8–13^). Conversely, data from Nepal, Uruguay and California showed increases of 11–30% in preterm birth rates^14–16^, whereas national data from Canada, Spain, Sweden and the United States indicated small or no changes^17–22^. In parallel, studies from low- and middle-income countries (LMICs; Nepal and Nigeria) and HICs (the UK and Italy) reported increases in stillbirth rates of 2–22% (refs. ^12,14,23,24^), but few studies analysed preterm birth and stillbirth simultaneously.

There have been several systematic reviews and meta-analyses examining the impact of pandemic restrictions on perinatal outcomes. These studies have generally found insufficient evidence to suggest an overall change in global preterm birth and stillbirth rates, but they have reported changes in certain subgroups^25–27^. For example, when restricting to studies from HIC settings, Chmielewska et al. found a decrease in preterm birth rates (crude odds ratio (OR) 0.91, 95% confidence interval (CI) 0.84–0.99; 795,105 pregnancies from 12 studies) and an increase in stillbirth rates (OR 1.28, 95% CI 1.07–1.54; 367,288 pregnancies from 12 studies)^27^. However, comparison across studies was hampered by methodological differences. Notably, only one study in the review accounted for pre-pandemic trends in preterm births in their analysis^10,28^. Additionally, most studies used facility-based data, which are difficult to interpret because changes in perinatal outcome rates at individual health facilities could reflect lockdown-induced changes in healthcare delivery (for example, diversion of high-risk births from one facility to another) rather than true population-level changes in perinatal outcomes. Indeed, a living systematic review and meta-analysis demonstrated important differences in the estimated impact of pandemic restrictions on preterm birth, depending on whether the study used single-centre (10% relative reduction: adjusted OR 0.90, 95% CI 0.86–0.94; 183,422 pregnancies from 20 studies) or regional/national-level data (no change: adjusted OR 0.99, 95% CI 0.94–1.03; 1,385,403 pregnancies from eight studies)^26^.

While methodological challenges have hindered robust conclusions on whether lockdowns led to reductions in preterm births, there were undoubtedly unprecedented health, social and economic impacts that occurred as part of lockdowns that could potentially lead to reductions in preterm birth rates^29^. The most well-established cause of spontaneous preterm birth is infection^30^, and it is plausible that an immediate and substantial reduction in circulating infections during lockdown, due to reductions in social interaction and increased hygiene measures^31,32^, could directly influence preterm birth rates. Additionally, observational studies have shown an increased risk of poor pregnancy outcomes at times of high air pollution, particularly in association with exposure in the third trimester^33,34^; thus, reductions in air pollution linked with lockdown could potentially have an immediate impact on preterm birth^35,36^. It is, however, also plausible that any reduction in preterm birth rates might signal that high-risk women were not receiving timely and quality maternity care^37^, and the reduction in preterm births may have been offset by an increase in stillbirths.

In this Article, given the uncertainties in the available evidence on the impact of COVID-19 pandemic lockdowns on perinatal outcomes, particularly where studies have not used population-based data, we aimed to conduct a rigorous, standardized analysis using high-quality data from across the world through the International Perinatal Outcomes in the Pandemic (iPOP) study. Specifically, we explored whether lockdowns in response to the first wave of the COVID-19 pandemic were associated with a change in preterm birth rates using interrupted time series (ITS) analysis, and whether any associations identified varied by country income level or by type or timing of preterm birth, or could be explained by changes in stillbirth rates. Detailed time-series data enabled us to use pre-lockdown trends in preterm birth and stillbirth rates to forecast the expected trend in these outcomes had lockdown not occurred, and compare these forecasted rates with the observed rates for each country individually and combined across countries in a meta-analysis.

## Results

### Study population and preterm and stillbirth rates

We included 52,067,596 births occurring between January 2015 and July 2020. Of these, 51,340,025 (98.6%) were from the 18 population-based datasets capturing whole countries or regions and 727,571 (1.4%) were from the 26 non-population-based datasets (Supplementary Table 1). A total of 3,115,628 births were from the lockdown period, that is, the first four months after the stringency score first exceeded 50 on the Oxford COVID-19 Government Response Tracker Lockdown Stringency Index (henceforth ‘Oxford Stringency Index^38^). As described in Supplementary Table 2 and Supplementary Fig. 1, non-population-based datasets from five countries were excluded from the analysis due to data availability and quality issues. Lockdowns remained above the threshold of 50 on the Oxford Stringency Index in most countries throughout the four month lockdown period used in this study, apart from Finland, Iceland, Norway and Switzerland (Supplementary Fig. 2).

**Fig. 1:**
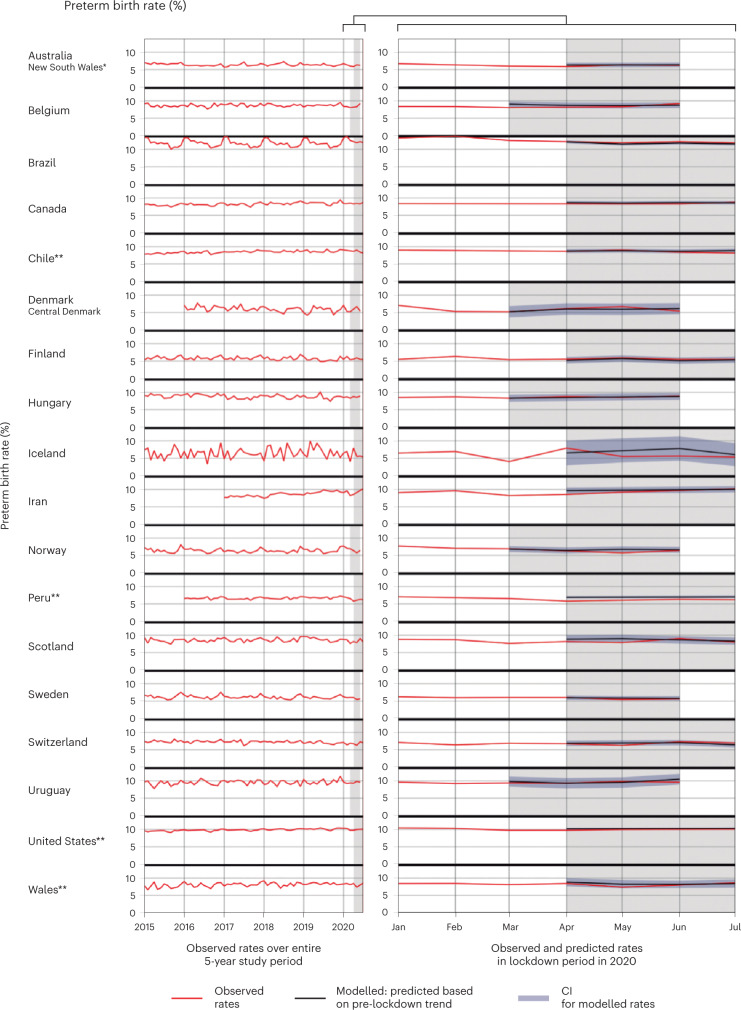
Trends in preterm birth rates among population-based datasets included in the iPOP Study; 2015 –2020. Observed rates of preterm birth (among all births 22 weeks onwards) over time (2015–2020) for countries providing population-based data, with the forecasted preterm birth rates and 95% CIs also plotted for the lockdown period. Lockdown period shown in shaded grey. Unless specified otherwise, preterm birth rates are the percentage of all births from 22 weeks onwards that were born before 37 weeks gestation. Left: entire study period (2015–2020) illustrating seasonality and trends over time. Right: 2020 period enlarged to show the observed and forecasted births during lockdown. Forecasted (‘modelled’) rates were estimated from a ‘pre-lockdown model’ that was used to forecast the expected rates of the preterm birth for each of the first four months of lockdown assuming lockdown had not occurred. *Preterm birth rates restricted to births from 24 weeks onwards; **Preterm birth rates restricted to live births only.

**Extended Data Fig. 1:**
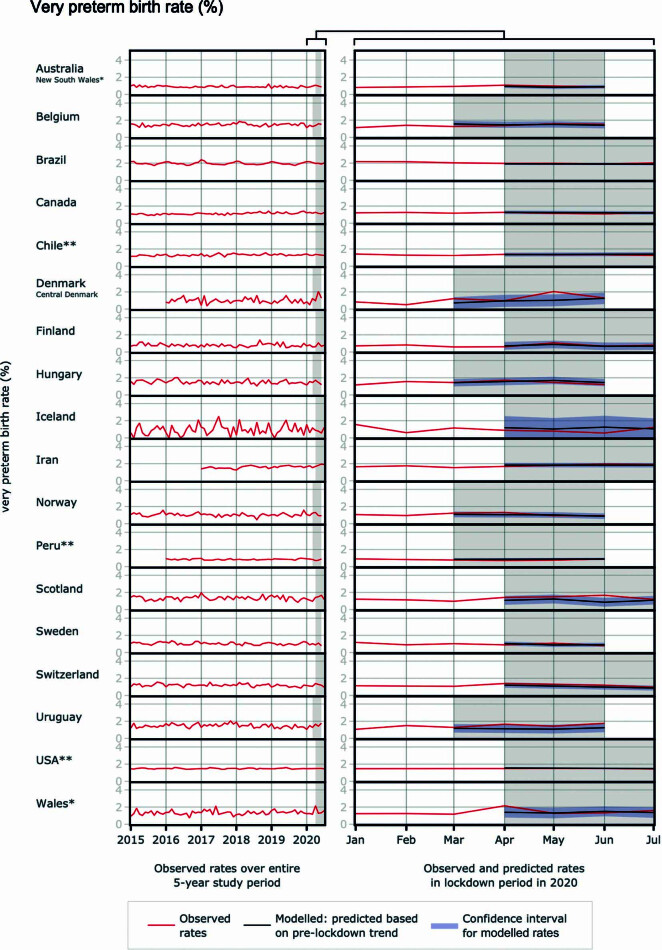
Observed rates of very preterm births (amongst all births 22 weeks onwards) over time (2015-2020) for countries with population-based data, with the forecasted very preterm births and 95% confidence intervals also plotted for the lockdown period. Lockdown period shown in shaded grey. Unless specified otherwise, very preterm birth rates are the percentage of all births from 22 weeks onwards that were born before 32 weeks gestation. Left panel: entire study period (2015-2020) illustrating seasonality and trends over time. Right panel: 2020 period enlarged to show the observed and forecasted very preterm birth rates during lockdown. Forecasted (‘modelled’) rates were estimated from a ‘pre-lockdown model’ which was used to forecast the expected rates of very preterm birth for each of the first four months of lockdown assuming lockdown had not occurred. *Very preterm birth rates restricted to births from 24 weeks onwards; **Very preterm birth rates restricted to live births only.

**Extended Data Fig. 2:**
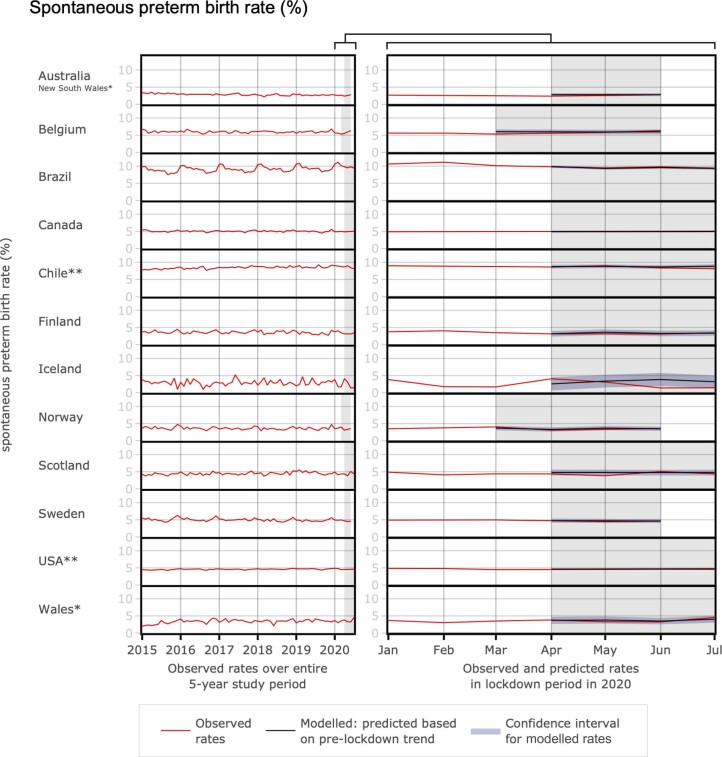
Observed rates of spontaneous preterm births (amongst all births 22 weeks onwards) over time (2015-2020) for countries with population-based data, with the forecasted spontaneous preterm births and 95% confidence intervals also plotted for the lockdown period. Lockdown period shown in shaded grey. Unless specified otherwise, spontaneous preterm birth rates are the percentage of all births from 22 weeks onwards that were born before 37 weeks gestation and where the birth was preceded by spontaneous contractions or preterm prelabour rupture of membranes. Left panel: entire study period (2015-2020) illustrating seasonality and trends over time. Right panel: 2020 period enlarged to show the observed and forecasted spontaneous preterm birth rates during lockdown. Forecasted (‘modelled’) rates were estimated from a ‘pre-lockdown model’ which was used to forecast the expected rates of spontaneous preterm birth for each of the first four months of lockdown assuming lockdown had not occurred. *Spontaneous preterm birth rates restricted to births from 24 weeks onwards; **Spontaneous preterm birth rates restricted to live births only.

**Extended Data Fig. 3:**
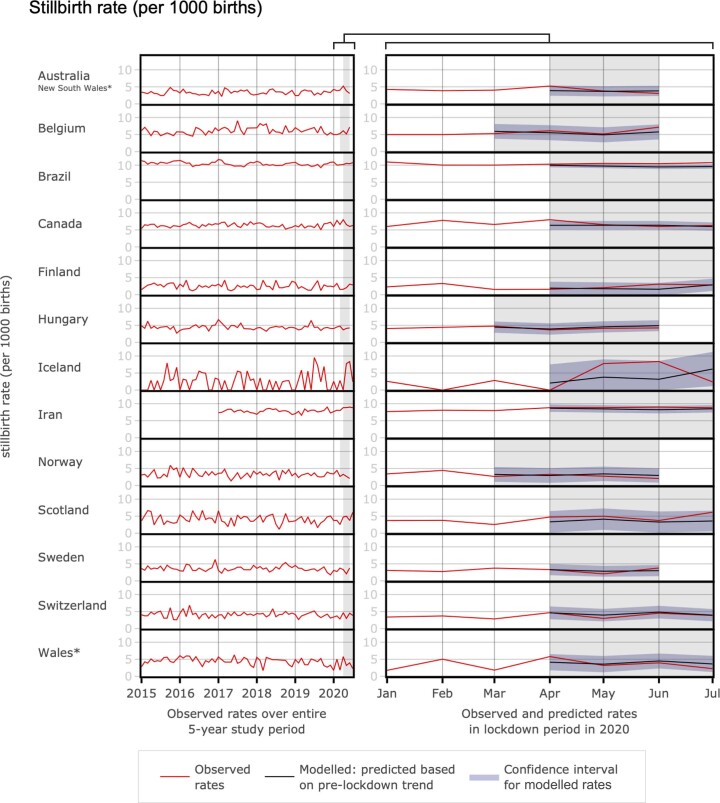
Observed rates of stillbirth (amongst all births 22 weeks onwards) over time (2015-2020) for countries with population-based data, with the forecasted stillbirth rates and 95% confidence intervals also plotted for the lockdown period. Lockdown period shown in shaded grey. Unless specified otherwise, stillbirth rates are the number of all births from 22 weeks onwards that were stillborn expressed per 1000 births. Left panel: entire study period (2015-2020) illustrating seasonality and trends over time. Right panel: 2020 period enlarged to show the observed and forecasted stillbirth rates during lockdown. Forecasted (‘modelled’) rates were estimated from a ‘pre-lockdown model’ which was used to forecast the expected rates of stillbirth for each of the first four months of lockdown assuming lockdown had not occurred. *Stillbirths rates restricted to births from 24 weeks onwards.

As shown in Table 1, among population-based datasets, the preterm birth rates (<37 weeks gestation) across the study period ranged from 5.8% (Finland) to 11.8% (Brazil); very preterm birth rates (<32 weeks gestation) from 0.8% (Finland and Peru) to 2.0% (Brazil); spontaneous preterm birth rates from 2.8% (New South Wales, Australia) to 9.2% (Brazil); and stillbirths from 2.5 per 1,000 births (Finland) to 10.4 per 1,000 births (Brazil). Temporal trends in preterm birth rates for each country are shown in Fig. 1, with equivalent plots for very preterm, spontaneous preterm birth and stillbirth rates in Extended Data (Extended Data Figs. 1–3). In the non-population-based data, there was wide variation in preterm and stillbirth rates both within and between countries (Table 2 and Supplementary Figs. 3–12).

**Table 1:** Data availability, overall preterm; very preterm; spontaneous preterm and stillbirth rates for population-based datasets, and data quality from 2015 to 2020 (exact timeframes vary by country as outlined in Supplementary Table 1)

							Data quality	
	Total number of births included	Monthly number of births, average	Preterm births (%)	Very preterm births (%)	Spontaneous preterm births (%)	Stillbirths per 1,000 births	Observed versus expected total number of births during lockdown^d^ (%)	Births missing gestational age (%)
Population-based data								
**Asia-Pacific**								
Australia, New South Wales^a^	518,281	7,853	6.4	0.9	2.8	3.4	−1%	0.1
**Middle East and North Africa**								
Iran	4,852,267	112,843	8.7	1.6	No data	7.9	+2%	0
**Europe**								
Belgium	665,086	10,077	8.6	1.5	6.0	6.1	No difference	0.02
Denmark, Central Region	66,481	1,231	6.0	1.0	No data	Not included^e^	No difference	1.3
Finland	278,376	4,155	5.8	0.8	3.6	2.5	No difference	0.2
Hungary	501,860	7,604	8.8	1.5	No data	4.4	+3%	0.02
Iceland	23,463	350	6.4	0.9	2.9	2.7	−5%	0.5
Norway	316,067	4,789	6.4	1.0	3.6	3.3	−1%	0.2
Scotland^b^	288,118	4,300	8.6	1.3	4.4	4.1	−1%	0.3
Sweden	586,914	8,892	6.2	1.0	5.0	3.6	+3%	0
Switzerland	486,357	7,259	7.2	1.2	No data	4.1	+1%	0.03
Wales^a^	176,964	2,641	8.1	1.4	3.4	4.4	−5%	0.4
**North America**								
Canada	1,610,511	24,037	8.3	1.1	5.0	6.4	−2%	0.6
United States^c^	20,979,669	313,129	9.9	1.5	4.6	No data	−1%	0.1
**Latin America and Caribbean**								
Brazil	16,356,490	244,127	11.8	2.0	9.2	10.4	−8%	2.4
Chile^c^	1,244,121	18,567	8.5	1.3	8.4	No data	−5%	0.3
Peru^c^	2,156,486	39,935	6.6	0.8	No data	No data	−7%	0.001
Uruguay	232,514	3,523	9.5	1.5	No data	No data	+8%	1.3

^a^Includes only births from 24 weeks onwards.

^b^Includes only births from 28 weeks onwards in the calculation for spontaneous preterm birth rates (as it is not possible to classify foetal losses at 22 and 23 weeks gestation as spontaneous or indicated).

^c^Includes only live births.

^d^Ascertained by comparing the observed total number of births in the lockdown period with the forecasted total number of births calculated using a Poisson time series, which accounted for preceding seasonal and yearly trends.

^e^Not included due to high levels of suppressed data.

**Table 2:** Data availability, overall preterm; very preterm; spontaneous preterm and stillbirth rates for non-population-based datasets included in the iPOP Study, and data quality from 2015 to 2020 (exact timeframes vary by country as outlined in Supplementary Table 1)

							Data quality	
	Total number of births included	Monthly number of births, average	Preterm births (%)	Very preterm births (%)	Spontaneous preterm births (%)	Stillbirths per 1,000 births	Observed versus expected total number of births during lockdown^b^ (%)	Births missing gestational age (%)
**Hong Kong**								
All public facilities (pooled)	199,134	3,064	8.4	1.4	No data	4.2	−13%	0
**Australia, Queensland**								
Facility 1	58,204	869	10.6	2.5	4.6	5.7	−2%	0
**Matlab, Bangladesh**								
Demographic surveillance area^a^	29,705	443	13.5	1.6	13.5	15.6	+7%	0.3
**Poland**								
Facility 1	8,287	126	7.0	0.3	4.9	4.1	−20%	0
Facility 2	42,243	640	15.6	4.4	13.5	8.8	−13%	0.2
**Washington State, United States**								
14 facilities (pooled)	90,586	2,107	10.0	1.7	6.3	4.2	+10%	0
**Mexico City, Mexico**								
Facility 1	10,084	235	28.9	6.0	10.8	51.0	+10%	0.8
**Ghana**								
Facility 2	12,452	290	21.3	9.5	11.9	17.5	+8%	17.7
Facility 3	7,724	188	24.5	14.2	9.6	20.5	+8%	6.2
Facility 4	8,450	197	25.3	13.9	11.5	18.1	+4%	9.3
Facility 5	13,208	194	24.2	14.4	9.1	20.6	+6%	6.1
Facility 6	13,325	333	24.1	8.5	13.6	17.7	−18%	21.3
Facility 7	15,818	406	19.1	6.2	10.6	20.0	+17%	13.9
Facility 9	15,473	360	19.8	7.7	11.0	17.5	+1%	17.1
**Kenya**								
Facility 1	34,773	527	4.0	1.4	No data	20.4	+5%	1.7
Facility 3	51,790	909	3.3	0.7	No data	13.3	−32%	0.6
**Nigeria**								
Facility 1	7,275	110	22.2	7.4	6.7	49.2	−6%	0.2
Facility 2	6,923	107	15.4	3.2	14.2	41.5	+1%	1.7
Facility 3	12,118	181	8.4	0.2	6.8	42.4	−11%	17.1
Facility 4	5,267	79	16.2	5.6	15.5	45.0	+6%	3.4
Facility 8	8,808	131	7.2	1.5	No data	55.1	−1%	8.0
Facility 9	7,252	110	16.0	4.2	No data	59.0	−10%	9.3
Facility 10	17,457	265	16.1	3.4	6.8	106.9	+22%	3.1
Facility 11	10,361	155	21.6	7.4	No data	77.1	−55%	6.3
Facility 12	14,479	216	9.4	2.4	5.7	54.2	+1%	2.5
**Uganda**								
Facility 2	26,375	394	9.5	3.3	3.0	16.2	+2%	1.6

^a^Includes only births from 28 weeks onwards for stillbirths.

^b^Ascertained by comparing the observed total number of births in the lockdown period with the forecasted total number of births calculated using a Poisson time series, which accounted for preceding seasonal and yearly trends.

### Data quality

Data quality was generally high in the population-based datasets, with most having <1% missing data on gestational age and <5% difference in observed versus expected total number of births during the lockdown period (Table 1). Among non-population-based datasets, there were low levels of missing data on gestational age (<1%) in datasets from Asia, Europe, North America and Latin America; however, some datasets from sub-Saharan Africa had substantial (up to 21%) missing information on gestational age. In addition, the total number of observed births differed by >10% (either increase or decrease) from expected during the lockdown period in some non-population-based datasets (Hong Kong, Poland and in some facilities in Ghana, Kenya and Nigeria) (Table 2). These quality issues among non-population-based datasets support our a priori decision to focus the primary analyses on population-based datasets.

### Association between lockdown and preterm birth

**Fig. 2:**
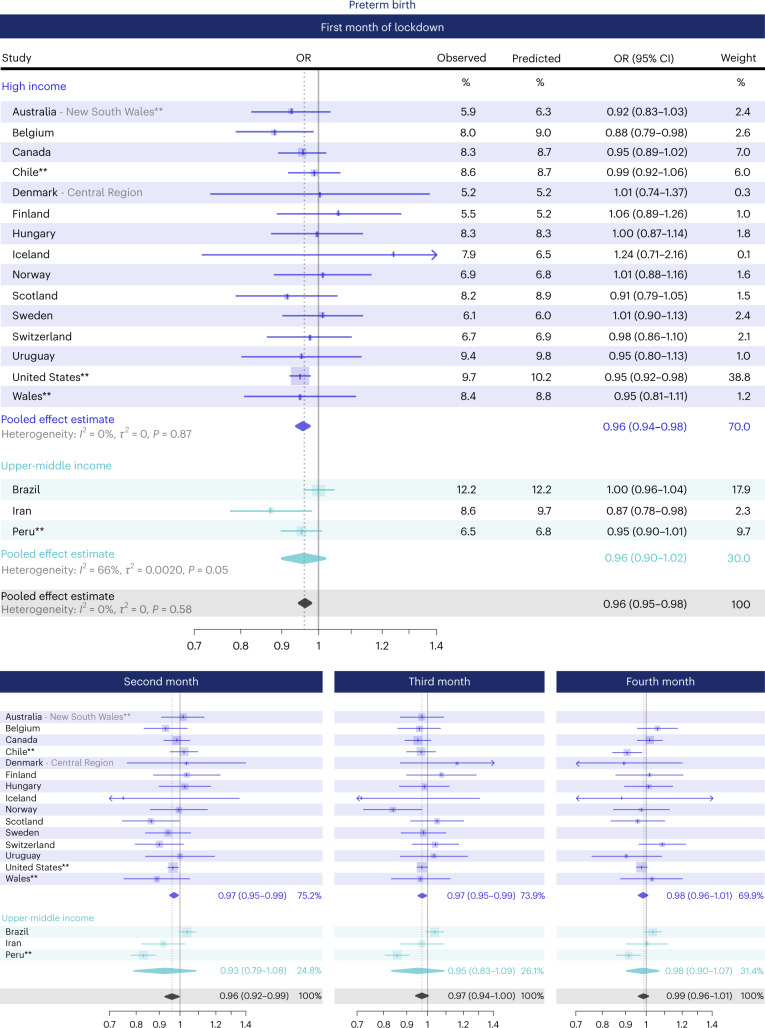
Association between lockdown and preterm birth rates in the iPOP Study in population-based datasets. Individual and pooled population-based estimates of the association between lockdown and the odds of preterm birth among all births 22 weeks onwards, stratified by time since lockdown. Individual country ORs (represented by boxes on plot) were calculated by comparing the observed odds of preterm birth with the forecasted odds of preterm birth from an ITS model that was fitted to pre-lockdown data. Horizontal lines surrounding each box on the forest plot are 95% CIs. Arrows indicate upper and/or lower bounds of the CI that are outside the x-axis limits. Pooled ORs (represented by diamonds on plot) for the association between lockdown and the odds of preterm birth were calculated using random-effects meta-analysis. Sample sizes for each country provided in Table 1. *Births from 24 weeks onwards **Live births only.

**Fig. 3:**
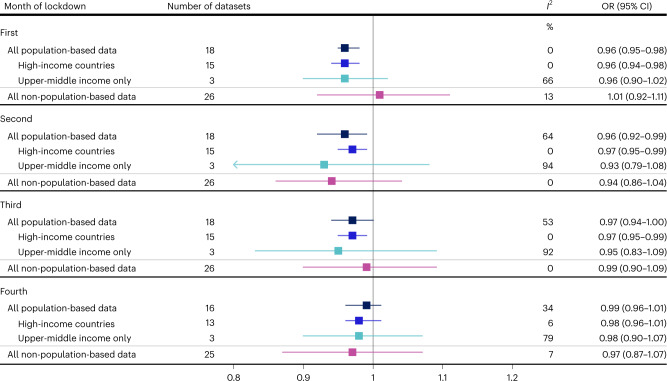
Pooled estimates of the association between lockdown and preterm birth rates in the iPOP Study. Pooled ORs capturing the association between lockdown and the odds of preterm birth, stratified by month of lockdown, type of data (population-based, non-population based) and income setting. Pooled ORs (represented by boxes on plot) were calculated using random-effects meta-analysis. Horizontal lines surrounding each box on the forest plot are 95% CIs for the OR. Arrows indicate upper and/or lower bounds of the CI that are outside the *x*-axis limits.

Figure 2 shows the country-specific OR for the impact of lockdown on preterm birth, for each month of lockdown (additional detailed plots: Supplementary Figs. 13–16). In the first month, for example, the OR for the impact of lockdown on preterm birth ranged from 0.87 in Iran (95% CI 0.78–0.98) to 1.24 in Iceland (95% CI 0.71–2.16). Our meta-analysis of population-based data indicated small reductions in preterm birth in the first (OR 0.96, 95% CI 0.95–0.98, *P* < 0.001), second (OR 0.96, 95% CI 0.92–0.99, *P* = 0.03), and third month (OR 0.97, 95% CI 0.94–1.00, *P* = 0.09) of lockdown, but none in the fourth month (OR 0.99, 95% CI 0.96–1.01, *P* = 0.34) (Figs. 2 and 3). Between-country heterogeneity (*I*^2^) was 0%, 64%, 53% and 34% for the first to fourth month of lockdown, respectively. Stratifying by country income level indicated similar reductions in the odds of preterm birth for both high and upper-middle-income country settings, with higher between-country heterogeneity among upper-middle-income countries (Fig. 3).

**Extended Data Fig. 4:**
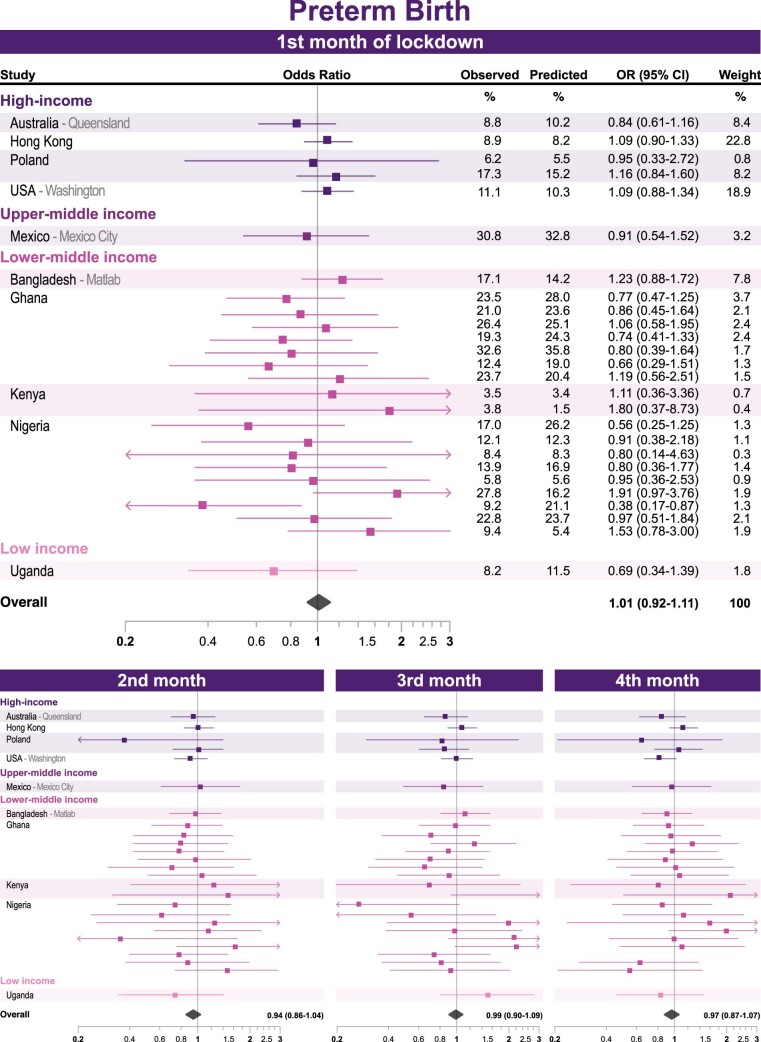
Individual and pooled non-population-based estimates of the association between lockdown and the odds of preterm birth among all births 22 weeks onwards, stratified by time since lockdown. Individual odds ratios (represented by boxes on plot) for each dataset were calculated by comparing the observed odds of preterm birth to the forecasted odds of preterm birth from a linear regression model that was fitted to pre-lockdown data. Horizontal lines surrounding each box on the forest plot are 95% confidence intervals. Pooled odds ratios (represented by diamonds on plot) for the association between lockdown and the odds of preterm birth were calculated using random-effects meta-analysis. Sample sizes for each dataset provided in Table 2.*Births from 24 weeks onwards; **Live births only.

There was a wider range of ORs across the non-population-based data with, for example in the first month of lockdown, ORs of 0.38 (95% CI 0.17–0.87) in one facility in Nigeria, and up to 1.91 (95% CI 0.97–3.76) in another facility in Nigeria (Extended Data Fig. 4 and Supplementary Fig. 17). There was no evidence for an association between lockdown and preterm birth in the meta-analysis of the non-population-based data (Fig. 3, Extended Data Fig. 4 and Supplementary Figs. 17–20).

For very preterm births, there was no evidence of an impact of lockdown over the four months of lockdown (Fig. 4, Extended Data Figs. 5 and 6 and Supplementary Figs. 21–28), with ORs for all population-based datasets varying between 1.00 and 1.02 and CIs spanning the null value. For spontaneous preterm births, in the subset of countries with data available, there were small relative decreases (3–4%) in the first three months following lockdown in HICs, but not in Brazil, the only upper-middle-income country providing these data (Fig. 5, Extended Data Fig. 7 and Supplementary Figs. 29–32). There was also evidence for a decrease in the fourth month of lockdown using only the non-population-based data (OR 0.88, 95% CI 0.78–0.99, *P* = 0.04, *I*^2^ = 0%) (Fig. 5, Extended Data Fig. 8 and Supplementary Figs. 33–36).

**Fig. 4:**
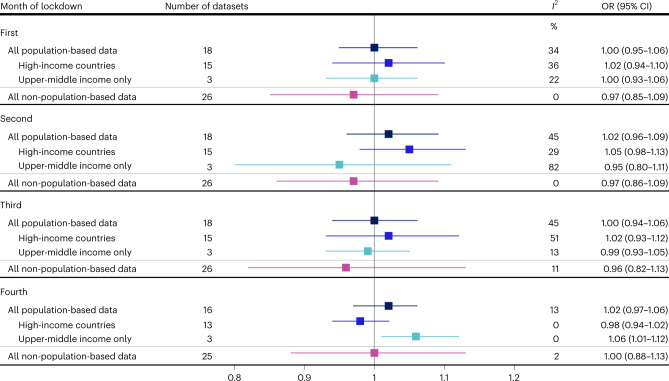
Pooled estimates of the association between lockdown and very preterm birth in the iPOP Study. Pooled ORs capturing the association between lockdown and the odds of very preterm birth (births at <32 weeks gestation), stratified by month of lockdown, type of data (population-based, non-population-based) and income setting. Pooled ORs (represented by boxes on plot) were calculated using random-effects meta-analysis. Horizontal lines surrounding each box on the forest plot are 95% CIs for the OR.

**Fig. 5:**
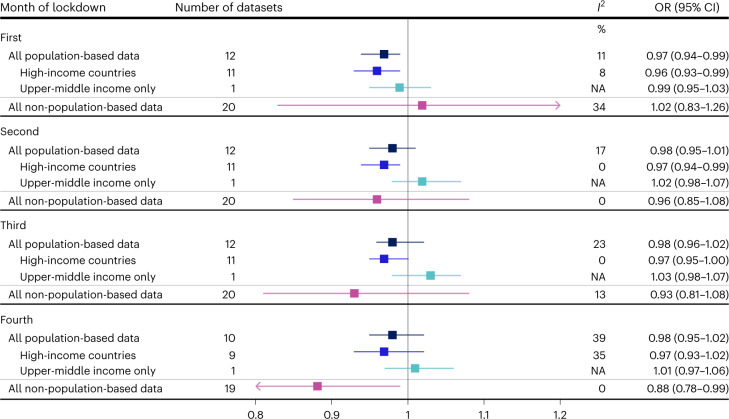
Pooled estimates of the association between lockdown and spontaneous preterm birth in the iPOP Study. Pooled ORs capturing the association between lockdown and the odds of spontaneous preterm birth, stratified by month of lockdown, type of data (population-based, non-population based) and income setting. Pooled ORs (represented by boxes on plot) were calculated using random-effects meta-analysis. Horizontal lines surrounding each box on the forest plot are 95% CIs for the OR. Arrows indicate upper and/or lower bounds of the CI that are outside the *x*-axis limits. NA, not applicable.

### Association between lockdown and stillbirths

**Fig. 6:**
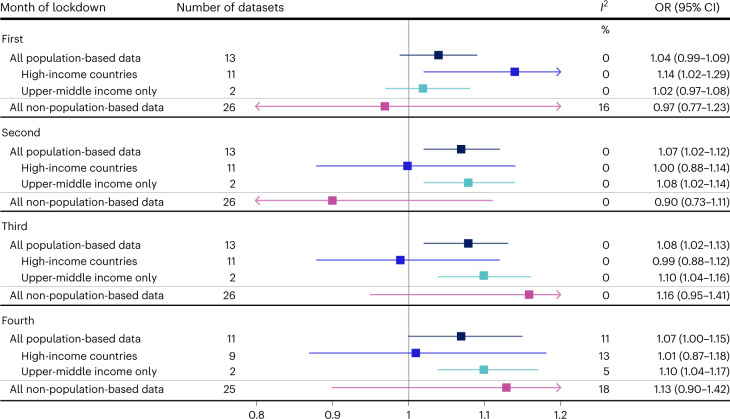
Pooled estimates of the association between lockdown and odds of stillbirth in the iPOP Study. Pooled ORs capturing the association between lockdown and the odds of stillbirth, stratified by month of lockdown, type of data (population-based, non-population based) and income setting. Pooled ORs (represented by boxes on plot) were calculated using random-effects meta-analysis. Horizontal lines surrounding each box on the forest plot are 95% CIs for the OR. Arrows indicate upper and/or lower bounds of the CI that are outside the *x*-axis limits.

**Extended Data Fig. 9:**
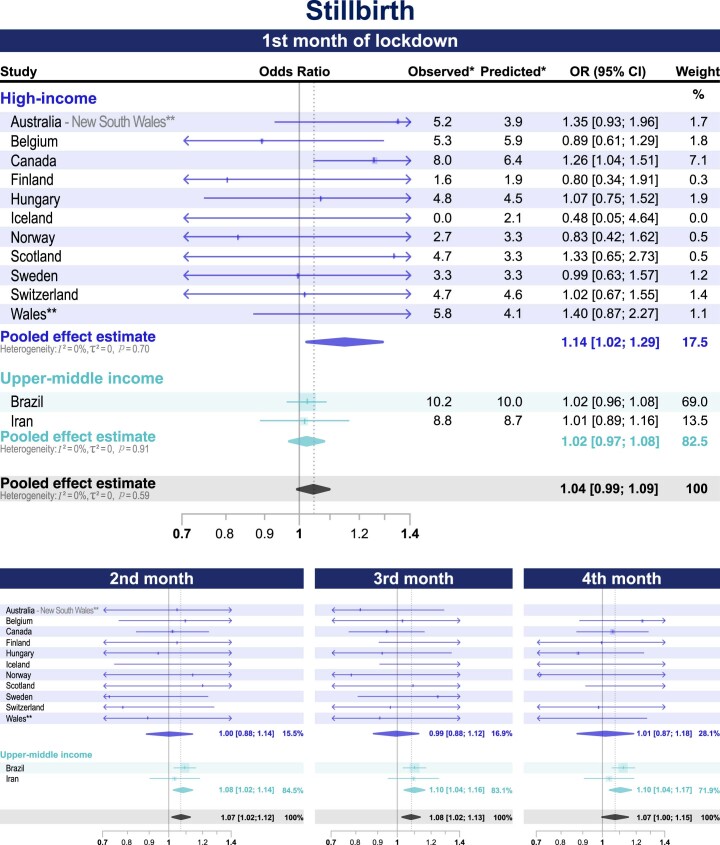
Individual and pooled population-based estimates of the association between lockdown and the odds of stillbirth among all births 22 weeks onwards, stratified by time since lockdown. Individual country odds ratios (represented by boxes on plot) were calculated by comparing the observed odds of stillbirth to the forecasted odds of stillbirth from an interrupted time series model that was fitted to pre-lockdown data. Horizontal lines surrounding each box on the forest plot are 95% confidence intervals. Pooled odds ratios (represented by diamonds on plot) for the association between lockdown and the odds of preterm birth were calculated using random-effects meta-analysis. Sample sizes for each country provided in Table 1. *Per 1000 births; **Births from 24 weeks onwards.

The OR for the impact of lockdown on stillbirth ranged from 0.80 in Finland (95% CI 0.34–1.91) to 1.35 in New South Wales, Australia (95% CI 0.93–1.96) in the population-based data in the first month of lockdown (Extended Data Fig. 9 and Supplementary Fig. 37). In the meta-analysis of the population-based datasets, we found no clear evidence of an impact of lockdown on stillbirth in the first month of lockdown overall (OR 1.04, 95% CI 0.99–1.09, *P* = 0.10, *I*^2^ = 0%), but an increase was observed when restricting to HICs (OR 1.14, 95% CI 1.02–1.29, *P* = 0.02, *I*^2^ = 0%), driven by Canada (OR 1.26, 95% CI 1.04–1.51, *P* = 0.02) (Fig. 6, Extended Data Fig. 9 and Supplementary Fig. 37). There was an increase in the odds of stillbirth across all population-based datasets in the second (OR 1.07, 95% CI 1.02–1.12, *P* = 0.001, *I*^2^ = 0%), third (OR 1.08, 95% CI 1.02–1.13, *P* = 0.004, *I*^2^ = 0%) and possibly fourth month (OR 1.07, 95% CI 1.00–1.15, *P* = 0.07, *I*^2^ = 11%) of lockdown. These ORs were driven largely by Brazil (Extended Data Fig. 9 and Supplementary Figs. 38–40), and when we restricted the meta-analysis to HICs only, we found no evidence for an association between lockdown and stillbirth in the second month (OR 1.00, 95% CI 0.88–1.12, *P* = 0.98, *I*^2^ = 0%), third month (OR 0.99, 95% CI 0.88–1.12, *P* = 0.89, *I*^2^ = 0%) and fourth month (OR 1.01, 95% CI 0.87–1.18, *P* = 0.86, *I*^2^ = 13%) of lockdown (Fig. 6).

**Extended Data Fig. 10:**
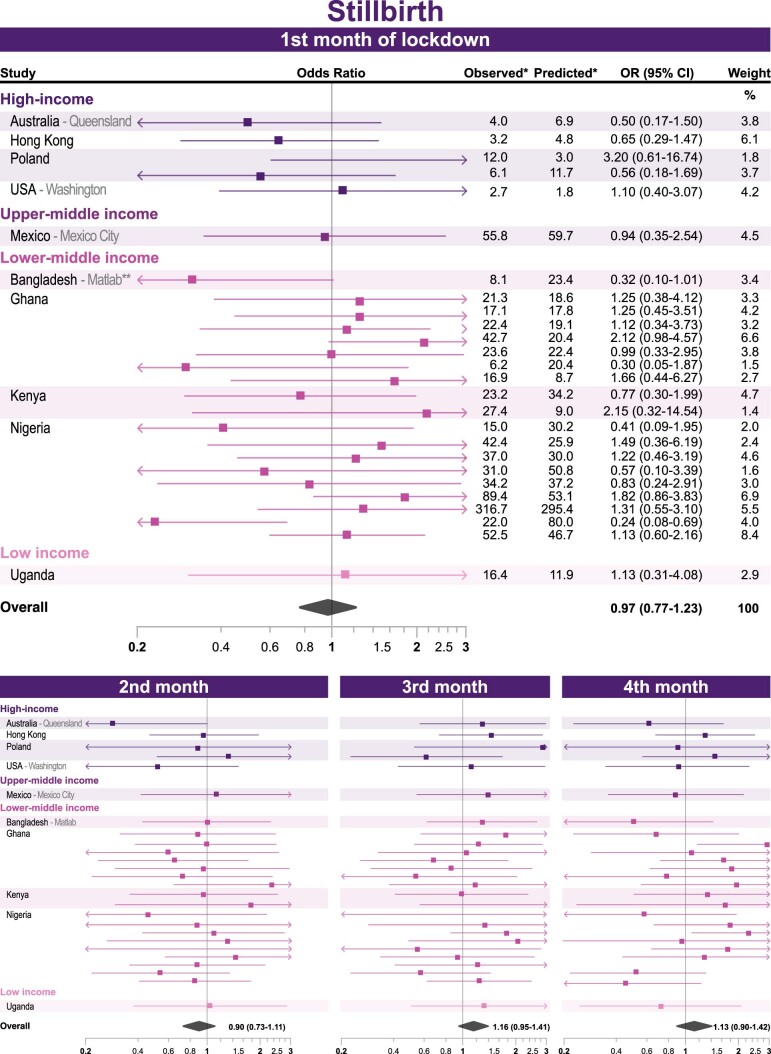
Individual and pooled non-population-based estimates of the association between lockdown and the odds of stillbirth all births 22 weeks onwards, stratified by time since lockdown. Individual odds ratios (represented by boxes on plot) for each dataset were calculated by comparing the observed odds of stillbirth to the forecasted odds of stillbirth from a linear regression model that was fitted to pre-lockdown data. Horizontal lines surrounding each box on the forest plot are 95% confidence intervals. Pooled odds ratios (represented by diamonds on plot) for the association between lockdown and the odds of stillbirth were calculated using random-effects meta-analysis. Sample sizes for each dataset provided in Table 2. *Per 1000 births; **Restricted to births from 28 weeks onwards.

In the non-population-based data, the ORs for stillbirth in the first month of lockdown ranged from 0.24 in a facility in Nigeria (95% CI 0.08–0.69) to 3.20 in a facility in Poland (95% CI 0.61–16.74) (Extended Data Fig. 10 and Supplementary Fig. 41). We observed increased odds of stillbirth for the third and fourth months of lockdown in the meta-analysis of non-population-based data with relatively low levels of between-study heterogeneity at 0% and 18%, respectively (Fig. 6, Extended Data Fig. 10 and Supplementary Figs. 41–44); however, CIs were wide and included the null value.

### Sensitivity analyses

Sensitivity analysis of the population-based data restricting the analysis to only live births (Supplementary Table 3) and restricting to only births from 28 weeks gestation onwards (Supplementary Table 4) led to negligible changes in the country-specific estimates of the impact of lockdown on preterm birth rates. Similarly, excluding data from Brazil and the United States, which together contributed slightly over 70% of the births included in the study, from the meta-analysis of the ORs for the association between lockdown and preterm birth among population-based datasets led to negligible changes in our estimates (Supplementary Table 5).

## Discussion

In this international study, we have reported on the impact of pandemic-related lockdowns on preterm birth and stillbirth. We included over 52 million births from 26 countries, largely derived from 18 population-based datasets from HICs and upper-middle-income countries. We observed small (3–4%) relative reductions in the overall rates of preterm birth following lockdown, although with some variation among countries. Reductions in spontaneous preterm birth rates were observed in HICs only, and no change in very preterm birth was observed. The observed decrease in preterm births did not appear to be driven by a reciprocal increase in stillbirth rates in HICs. We did, however, find evidence for increases in stillbirth in Brazil in the second, third and fourth months of lockdown. It remains plausible that some reduction in preterm birth rates was linked to increased stillbirth rates in HICs, but we had limited power to detect this due to the relatively small number of stillbirths. Our patient partners’ interpretation of these results are provided in Supplementary Discussion.

Multiple studies have assessed the effects of pandemic lockdowns on perinatal outcomes following initial reports of dramatic reductions in preterm birth rates^8,10,11^, and several meta-analyses have been conducted^25–27^. However, there have been important differences in data quality across prior studies, many of which did not apply analytical approaches that could account for pre-pandemic trends in perinatal outcomes^28^. Notably, few studies have included both preterm birth and stillbirth rates, despite the importance of considering perinatal outcomes together^39,40^. Our findings provide evidence by applying an ITS design to high-quality population-based data from 18 countries, and evaluating potentially competing outcomes (that is, preterm birth and stillbirth) in parallel. Even though we used the same analytical approach across data from different countries, between-country differences in the association between lockdown and both preterm birth and stillbirth rates were seen. These could be driven by contextual differences in the implementation of lockdown and differences in the impact of lockdown, which in turn may be driven by the resilience of health or social systems.

Lockdowns had important and diverse impacts on several exposures known to influence preterm birth, offering some possible explanations for the small reductions observed in our study. For spontaneous preterm birth, although the aetiology is poorly understood, putative mediating factors include reductions in air pollution and, in particular, non-COVID infections, both of which were shown to decline across a diverse range of countries, albeit to varying degrees^32,35,41^. It is possible that a reduction in physician-initiated preterm births also contributed to the overall reduction in preterm birth in some settings^6,42^, although we could not investigate this directly, as data on medically indicated preterm births were not available for all countries and could not be reliably inferred. The increase in stillbirth with lockdown in some countries might reflect reduced access to timely quality antenatal and intrapartum care^43^. As our findings represent the average impact of lockdown across populations, we cannot differentiate the relative contribution of specific factors, nor whether the impact of lockdown differed between specific population subgroups. For example, an increased risk of preterm birth in some women (for example, due to reduced access to care) might have been offset at the population level by public health responses reducing other risk factors, such as air pollution and infectious diseases other than COVID-19.

Using aggregate data, it was not possible to distinguish the impacts of severe acute respiratory syndrome coronavirus 2 (SARS-CoV-2) infection from those of pandemic-related restrictions. Relative to the essentially universal exposure of all pregnant women to lockdowns, only a small fraction^19^ experienced SARS-CoV-2 infection at this early stage of the pandemic. As SARS-CoV-2 infection increases the risk of both preterm birth and stillbirth^44–46^, it is possible that our results have underestimated the impact of lockdown on preterm birth and overestimated the impact on stillbirth, although any influence would be minimal given the relatively much smaller proportion of women experiencing infection compared with the broader effects of lockdown.

Our results highlight the paucity of population-based data in many settings, and the challenges of interpreting non-population-based data to assess changes in perinatal outcomes over time. First, in some countries, we observed large variation in preterm birth and stillbirth rates between facilities. These might reflect differences in case mix as well as challenges in accurate reporting of key variables, particularly in estimating gestational age when routine antenatal ultrasound is unavailable. Second, some facilities within the same country documented markedly different impacts of lockdown on preterm birth and stillbirth rates. In some countries, there were dramatic shifts in how and where pregnant women accessed intrapartum care^14^, urging caution in the interpretation of results from studies of single facilities. In addition, the paucity of population-based data in LMICs—where the majority of preterm births and stillbirths occur^30,47,48^—was striking. We made extensive efforts to identify high-quality data from across different country income levels, including iterative development of the data collection tools with groups from a range of different countries and, in consideration of the more intensive data preparation required in some countries to harness data on perinatal outcomes, special funding allocations to support the preparation of data from LMIC settings. While there have been substantial efforts globally to improve perinatal data and outcomes through stillbirth and preterm birth prevention initiatives, such as Every Newborn Action Plan^49^ and parent-led global organizations such as the International Stillbirth Alliance^50^, we echo previous calls for the urgent need to develop systems that routinely capture high-quality data on perinatal outcomes with standardized definitions across countries^51,52^.

The strengths of our study include the broad global coverage, use of pre-defined and internationally recognized outcome measures, and analytical approaches to account for time trends and seasonal patterns in perinatal outcomes^53^, as well as differentiation between population and non-population-based data and country income settings.

We acknowledge several limitations. First, we defined onset of lockdown as the month during which a country first exceeded 50 on the Oxford Stringency Index^38^. This is a crude measure to approximate the severity of pandemic-related restrictions in each country as a whole; it does not reflect within-country variations or individual experiences in lockdown measures. The Oxford Stringency Index also does not capture variations in access to maternity and healthcare nor provide information on the extent to which restrictions were enforced or followed. This is likely to be particularly problematic for large countries such as Brazil and the United States, but, unfortunately, subnational data on perinatal outcomes were not available for this study. Second, ITS analyses are vulnerable to confounding by unmeasured events occurring simultaneously to the intervention that might also impact the outcomes. Third, we used aggregate data and could not differentiate within-population differences on the impact of lockdown measures, which is likely to vary by socio-economic status, region and age. Fourth, as we focused on the association between lockdown and perinatal outcomes for the first four months following the lockdowns in response to the first wave of COVID-19, we mainly captured the potential impact of lockdown on pregnancies that were in their third trimester at the start of lockdown; further studies should be conducted to assess whether there was an association between lockdown and perinatal outcomes for pregnancies that were at earlier gestations in lockdown. Fifth, where we found no evidence for an association (for example, for stillbirth, very preterm birth and spontaneous preterm birth in all or some settings), we cannot rule out that there was no change as these were relatively rare with wide CIs. The use of equivalence tests to formally test whether there was no evidence for a clinically meaningful change in our outcomes was considered but ultimately not conducted as there is no minimum clinically meaningful difference for stillbirth or preterm births, with any change being of interest. Finally, the interpretation of our results is limited by difficulties with data capture, population coverage and data quality from some countries. We therefore conducted separate analyses for population-based data considered to be of high quality, yielding more robust estimates.

In summary, this international study provides evidence on global changes in preterm birth and stillbirth across 26 countries during the initial COVID-19 pandemic lockdowns. Overall, we observed a 3–4% relative reduction in the preterm birth rate during the first three months of lockdown based on population-based data from HICs and upper-middle-income countries. This decrease in preterm births did not appear to be linked with an increase in stillbirths in most settings. Consistent evidence of an increase in stillbirths was only observed in Brazil following lockdown, the causes of which certainly warrant further exploration. Although relatively small, the observed changes in preterm birth are meaningful at the population level: assuming the observed decline during lockdown is real and consistent worldwide, our findings suggest that nearly 50,000 preterm births (or approximately four per 1,000 live births) were averted in the first month of lockdown alone, based on a global pre-pandemic preterm birth rate of 10.6% (ref. ^1^). Understanding the underlying pathways linking lockdown with the reduction in preterm births could have implications for clinical practice and policy. Our study also highlights the need to develop further capacity for high-quality and appropriate standardized data collection in LMICs^54^. Finally, the iPOP platform offers novel opportunities to rapidly conduct harmonized perinatal health research globally during the COVID-19 pandemic and beyond.

## Methods

We engaged with national and subnational data custodians to standardize and analyse aggregate-level data on monthly numbers of births stratified by gestational age from population-based data sources, and to conduct exploratory analyses using non-population-based data sources. Detailed time-series data enabled us to use pre-lockdown trends in preterm birth and stillbirth rates to forecast the expected trend in these outcomes had lockdown not occurred, and compare these forecasted rates with the observed rates for each country individually and combined across countries in a meta-analysis. The study was conducted using a common protocol^55^ and reported according to the Strengthening the Reporting of Observational Studies in Epidemiology (STROBE) guideline^56^.

### Ethical considerations

Contributors from countries where the data were not publicly available obtained ethics approval from their respective institutional review boards (Supplementary Table 6). We did not seek ethical approval for publicly available data sources (Supplementary Table 6). All data contributors completed a Data Contribution Agreement, which outlined the terms and conditions for uploading and storing data to the SAIL Databank^57^.

### Study data and population

We collected aggregate data from 26 countries (Supplementary Table 1). We considered data sources as population-based if they captured more than 90% of births in the region or country, and non-population-based if coverage was ≤90%. There were 18 national and regional population-based data sources that, where possible, included all recorded births from 22 + 0 weeks gestation between January 2015 and July 2020. Births recorded as ≥45 + 0 weeks gestation were censored as unfeasible gestation of birth. Data were also included in the analyses if they were available for a shorter pre-pandemic period (Denmark, Iran and Peru), for live births only (Chile, Peru and the United States), or used a slightly different cut-off for the lower limit of gestational age (≥24 + 0 weeks gestation in New South Wales, Australia and Wales, UK).

Data available from low- and lower-middle-income country settings were exclusively non-population-based, and we therefore included non-population-based data as part of the main analysis in a deviation from the original protocol^55^, to provide insights across a range of countries by income levels. There were 26 non-population-based data sources from ten countries, which included data from individual health facilities (23 datasets from seven countries), pooled data from a group of health facilities (two datasets from two countries) and demographic surveillance sites (one dataset from one country) (Supplementary Table 1). For Australia and the United States, there were both population-based and non-population-based data sources included in the analysis; the data sources from Australia covered different regions of the country whereas data sources for the United States covered some overlapping regions but were not included together in any analysis (as described below).

To ensure data and measures from different settings were comparable, consistent and coherent, we developed a detailed protocol, including standardized outcome definitions and data collection templates^55^, and stored and analysed the standardized data in the Secure Anonymized Information Linkage (SAIL) Databank. We collected information on national income levels from the World Bank^58^ (Supplementary Table 1). In our study protocol^55^, we proposed to additionally collect national-level data on air pollution, adherence to lockdown, COVID-19 rates, world region and parental leave policy; we did not ultimately include these data due to (1) not being able to identify readily available reliable data for all our study settings (air pollution and adherence to lockdown) or (2) finding little or no variation between the included datasets beyond that captured by country-income level (COVID-19 rates, world region and parental leave policy).

### Defining lockdown

For each country, we defined the start of lockdown using the Oxford Stringency Index^38^. In brief, this index collects information on different social, health and economic government policies instituted in response to the COVID-19 pandemic.

We considered the onset of a country’s initial lockdown as the date at which the stringency score first exceeded 50 on the Oxford Stringency Index (range 0–100). This cut-off was pre-specified in the study protocol and based on expert advice. For dates of lockdown that occurred between the 1st and 15th of the month, the first month of lockdown was assigned to that month; for dates after the 15th, the first month of lockdown was assigned to the following month. As described below, we explored the impact on perinatal outcomes in the first four months from a country’s initial lockdown, regardless of whether the Oxford Stringency Index dropped below 50 during this time. We restricted the analysis to the first four months to facilitate comparison between different countries included in this study; this was when the strictest lockdowns were in place in response to the first wave of COVID-19, with increasing variability between countries beyond this timeframe.

### Defining perinatal outcomes

Data contributors recorded monthly numbers of births categorized into pre-specified gestational age groups, according to our data collection template. The outcome definitions aligned with global standard definitions for preterm birth and stillbirth^59,60^ and were developed in consultation with our international collaborators to ensure that all data contributors captured these outcomes consistently.

For each month, we calculated the preterm birth rate per 100 births, as the number of births from 22 + 0 to 36 + 6 weeks gestation divided by the total number of births^61^. We calculated the very preterm birth rate per 100 births as the number of births from 22 + 0 to 31 + 6 weeks gestation divided by the total number of births. We estimated the spontaneous preterm birth rate per 100 births as the number of births from 22 + 0 to 36 + 6 weeks gestation with spontaneous onset divided by the total number of births. The preterm, very preterm and spontaneous preterm birth rates were calculated, where available, using all births and live births only for settings where data on stillbirths were not available. We were not prescriptive in how data contributors should identify and define spontaneous births, beyond specifying that these should capture births preceded by spontaneous contractions or preterm prelabour rupture of membranes. Further details of the methods used to estimate gestational age across the different datasets are provided in Supplementary Table 1. The stillbirth rate was expressed per 1,000 births and calculated by dividing the number of stillbirths occurring from ≥22 + 0 weeks gestation by the total number of births.

### Data analysis

A detailed description of the steps to clean and prepare the data before undertaking the analysis is provided in Supplementary Methods. In brief, we evaluated data quality and completeness of each dataset by: (1) assessing data completeness, including calculating the percentage of births missing gestational age; (2) examining for outliers in perinatal outcome rates; and (3) assessing whether there was any evidence for a change in the documented number of births after lockdown which, given the early stage of the pandemic when fertility will not have been affected, would suggest that women were giving birth in different locations or there were changes in recording practices (further details on analytical procedures in Supplementary Methods). Any population-based datasets where there was a relative change of a 10% or more increase or decrease in the number of observed compared with expected total births following lockdown were excluded from the population-based analysis, and analysed as a non-population-based dataset.

For each country-specific population-based dataset, we undertook an ITS analysis^62^ to model the effect of lockdown on perinatal outcomes. First, we fitted weighted ITS models on the entire time series of the monthly log(odds) of the outcomes. Weights were based on the total number of births per month; imputed values for missing data (Supplementary Methods) were down-weighted to one (minimum possible number of births) to reduce bias from missing observations. Models accounted for seasonality (with inclusion of month as a fixed effect) and long-term temporal trends, and we allowed the within-period trend and intercept to be different for the pre-lockdown and lockdown periods. Given that countries could have different trends in perinatal outcomes, we fitted five different potential models for each outcome for each country evaluating the trend as a linear, square, quadratic, logarithmic and second-order polynomial effect. The model with the lowest Akaike Information Criterion was chosen as the best fit model^63^. We assessed the goodness of fit of the best model by examining the standardized residuals. Second, to compare the forecast of the best fit model to the post-lockdown observed values, we refitted the model to the pre-lockdown observations using the same trend effect selected through the Akaike Information Criterion. This ‘pre-lockdown model’ was then used to forecast the expected rates of the perinatal outcomes for each of the first four months of lockdown assuming lockdown had not occurred. Plots of the observed and forecasted rates were used to visualize trends in outcomes over time. We calculated the OR between the observed odds and the forecast odds of each perinatal outcome for each of the first four months of lockdown, a time period chosen to capture when lockdowns in response to the first wave of COVID-19 were implemented. We specified a priori to analyse each of the first four months of lockdown separately, as we hypothesized that the association between lockdown and perinatal outcomes would vary by month of lockdown given how rapidly public health measures evolved during this time. To analyse the non-population-based data, we used a linear regression model (rather than an ITS model) to forecast the log(odds) of perinatal outcomes in the first to fourth month of lockdown assuming lockdown had not occurred. This was due to non-population based datasets varying in data availability with respect to the pre-lockdown study period, frequency of reporting of outcomes, and degree of missingness. To capture changes by season and annual trends pre-lockdown in our forecasted estimates, the model included month (categorical) and year (continuous), with year also included as a squared term to account for settings with non-linear changes in the perinatal outcome rates over time. We then calculated the OR quantifying the impact of lockdown on the perinatal outcomes by dividing the observed odds of each perinatal outcome by the forecasted odds for each of the first four months of lockdown.

The ORs from each dataset for each perinatal outcome at each month after lockdown were pooled using random-effects meta-analysis^64^, and this was done separately for the population-based data and the non-population-based data. For the population-based data, we stratified the meta-analysis by country income level (where sufficient datasets for each category permitted): high income versus upper-middle income. For non-population-based data, we used a three-level meta-analysis model to account for the dependency of observations of the impact of lockdown between facilities in the same country^65^. The *I*^2^ statistic, which captures the percentage of the variability in the ORs between countries that is due to heterogeneity rather than sampling error, was used to assess for evidence of between-country heterogeneity in the ORs^66^. We did not conduct equivalence tests to assess whether there was evidence that there was no association between the pandemic and our outcomes, as these tests require identifying a minimum clinically significant difference below which we would conclude that there was no change in our outcomes. There is no clear clinically significant difference that can be used for preterm birth or stillbirth, with any increase being of concern. Where relevant, we report *P* values for the probability of observing a relative difference in our outcomes at least as big as that in our data under the assumption that there was no association between the pandemic and our outcomes.

All analyses were performed in R version 4.1.1.

### Sensitivity analyses

We conducted three sensitivity analyses. First, to assess the potential impact of including datasets that only provided data on preterm birth among live births (rather than all births, 3/18 datasets) in the main analysis, we conducted ITS analysis restricted to live births among datasets which also provided data on all births (*n* = 15 datasets). Second, to evaluate the impact of including datasets with a different lower limit for gestational age in the main population-based analysis for preterm birth, we restricted the time-series analysis to births from 28 weeks gestational age onwards, the lower threshold recommended by the World Health Organization for international comparisons^60^. Third, we also conducted a sensitivity analysis for our meta-analysis of the association between lockdown and preterm birth among all population-based datasets, excluding Brazil and the United States, which together contributed slightly over 70% of the births included in the study.

### Public and patient involvement

Parent representatives from four national patient partner organizations were included from the inception of the iPOP study to inform the common goal of timely implementation of quality research. We used mechanisms to ensure meaningful collaboration through inclusion on meeting agendas and facilitating meeting processes so that everyone had an equal voice to ensure patient partners were treated with mutual respect. Patient partners from Brazil, Canada, Hungary and Ireland co-developed the iPOP protocol, attended all iPOP meetings to ensure meaningful collaboration, edited and provided input to this manuscript, and are continuing to co-build meaningful and innovative knowledge translation strategies.

### Reporting summary

Further information on research design is available in the Nature Portfolio Reporting Summary linked to this article.

## Supplementary information

Supplementary Information Supplementary methods, discussion, Figs. 1–44, Tables 1–6 and references.

Reporting Summary

## Figures and Tables

**Extended Data Fig. 5:**
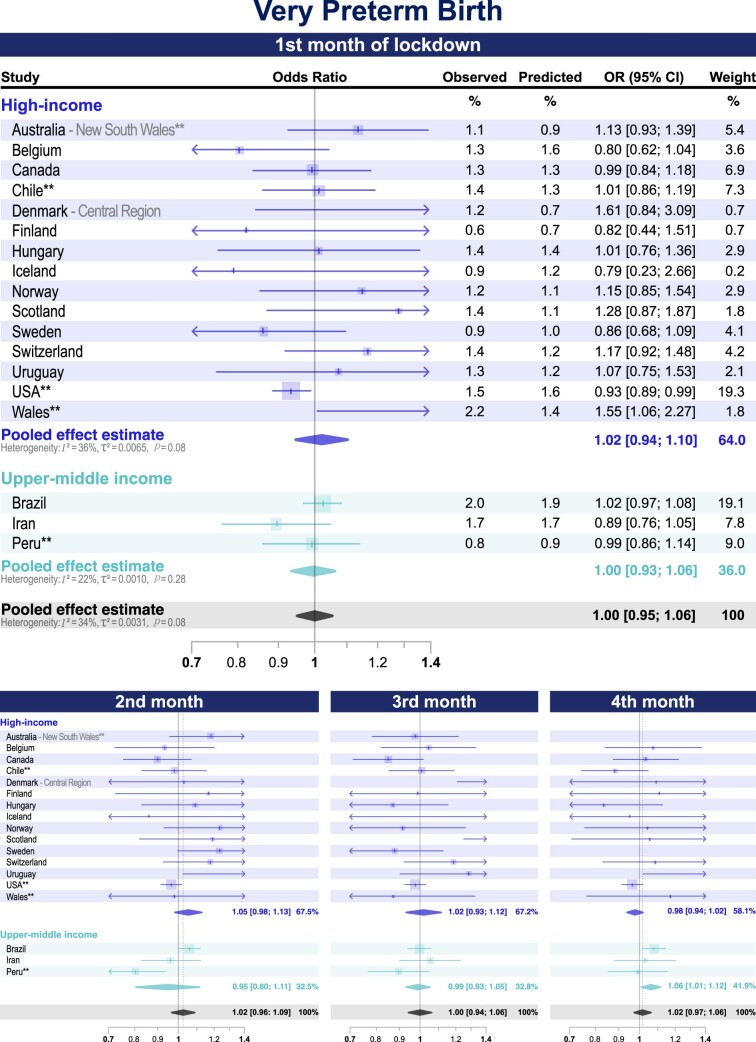
Individual and pooled population-based estimates of the association between lockdown and the odds of very preterm birth among all births 22 weeks onwards, stratified by time since lockdown. Individual country odds ratios (represented by boxes on plot) were calculated by comparing the observed odds of very preterm birth to the forecasted odds of very preterm birth from an interrupted time series model that was fitted to pre-lockdown data. Horizontal lines surrounding each box on the forest plot are 95% confidence intervals. Pooled odds ratios (represented by diamonds on plot) for the association between lockdown and the odds of very preterm birth were calculated using random-effects meta-analysis. Sample sizes for each country provided in Table 1. *Births from 24 weeks onwards; **Live births only.

**Extended Data Fig. 6:**
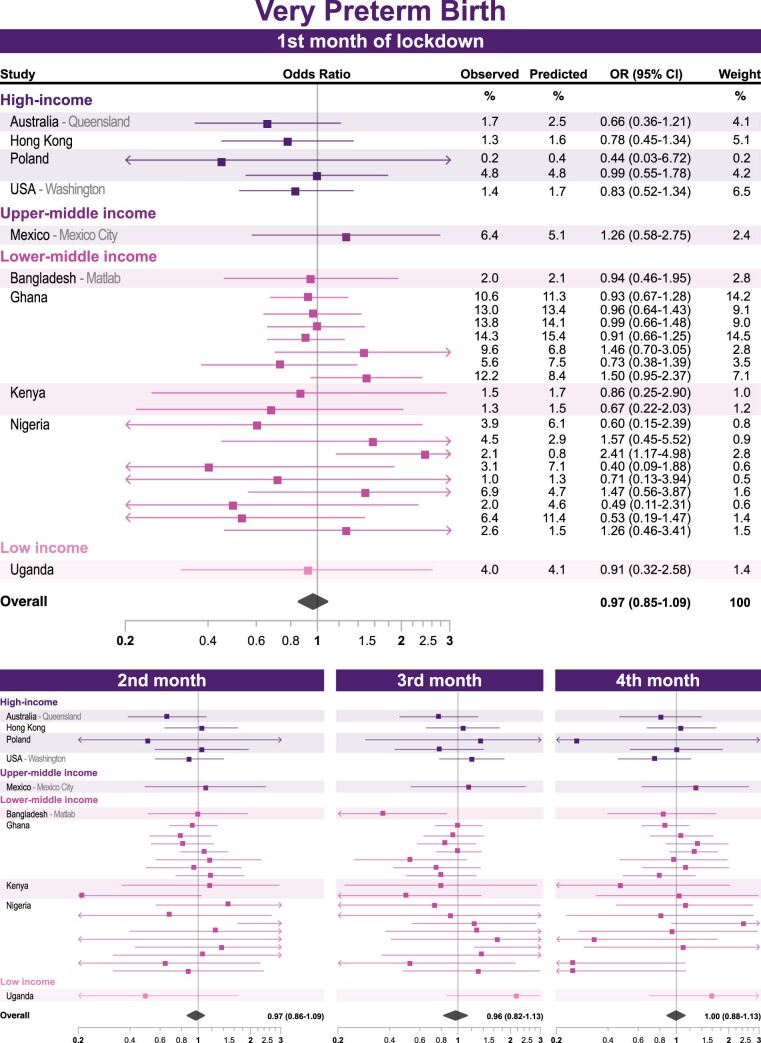
Individual and pooled non-population-based estimates of the association between lockdown and the odds of very preterm birth among all births 22 weeks onwards, stratified by time since lockdown. Individual odds ratios (represented by boxes on plot) for each dataset were calculated by comparing the observed odds of very preterm birth to the forecasted odds of very preterm birth from a linear regression model that was fitted to pre-lockdown data. Horizontal lines surrounding each box on the forest plot are 95% confidence intervals. Pooled odds ratios (represented by diamonds on plot) for the association between lockdown and the odds of very preterm birth were calculated using random-effects meta-analysis. Sample sizes for each dataset provided in Table 2.

**Extended Data Fig. 7:**
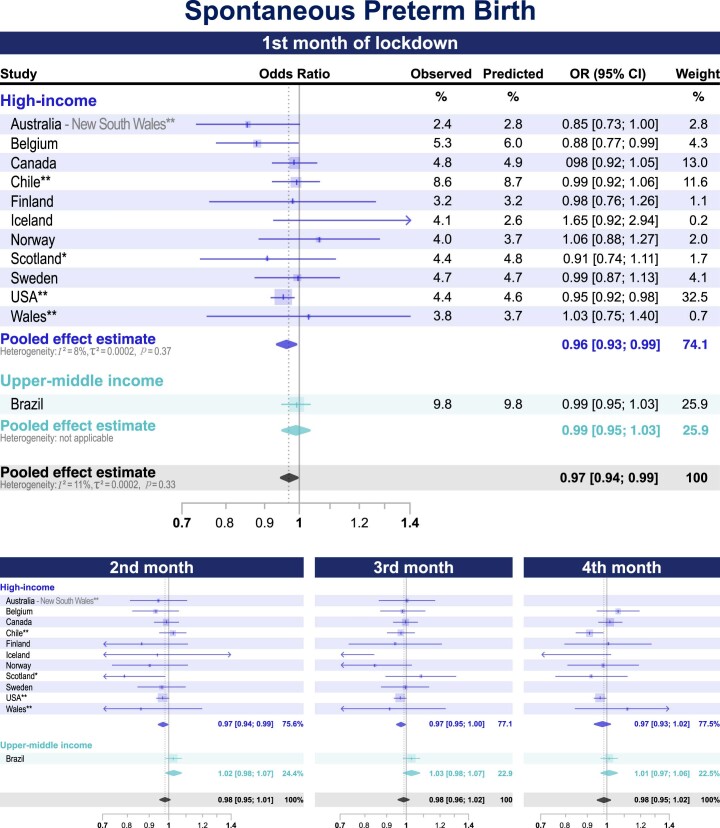
Individual and pooled population-based estimates of the association between lockdown and the odds of spontaneous preterm birth among all births 22 weeks onwards, stratified by time since lockdown. Individual country odds ratios (represented by boxes on plot) were calculated by comparing the observed odds of spontaneous preterm birth to the forecasted odds of spontaneous preterm birth from an interrupted time series model that was fitted to pre-lockdown data. Horizontal lines surrounding each box on the forest plot are 95% confidence intervals. Pooled odds ratios (represented by diamonds on plot) for the association between lockdown and the odds of spontaneous preterm birth were calculated using random-effects meta-analysis. Sample sizes for each country provided in Table 1. *Births from 24 weeks onwards; **Live births only.

**Extended Data Fig. 8:**
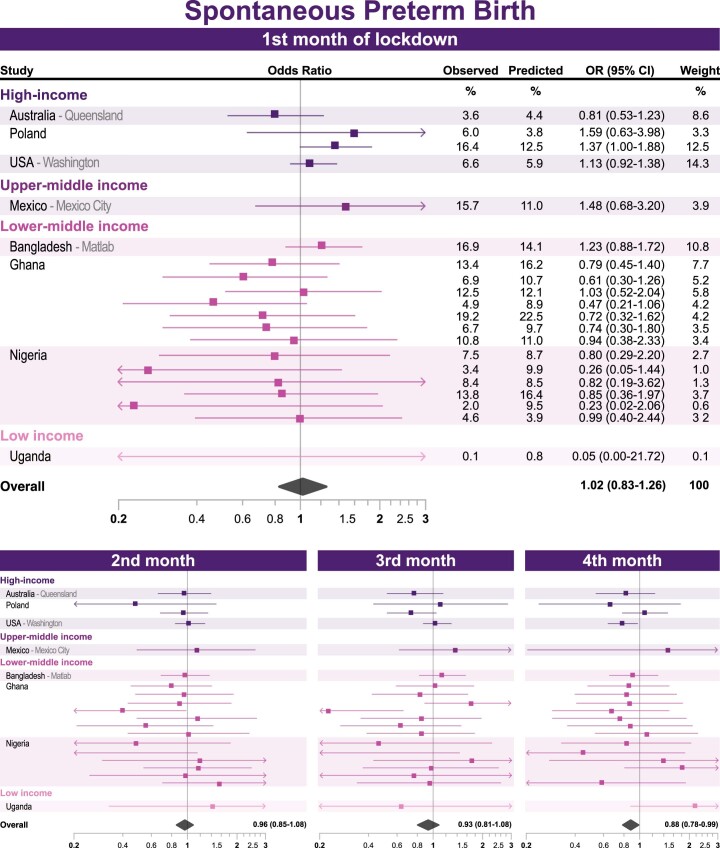
Individual and pooled non-population-based estimates of the association between lockdown and the odds of spontaneous preterm birth among all births 22 weeks onwards, stratified by time since lockdown. Individual odds ratios (represented by boxes on plot) for each dataset were calculated by comparing the observed odds of spontaneous preterm birth to the forecasted odds of spontaneous preterm birth from a linear regression model that was fitted to pre-lockdown data. Horizontal lines surrounding each box on the forest plot are 95% confidence intervals. Pooled odds ratios (represented by diamonds on plot) for the association between lockdown and the odds of spontaneous preterm birth were calculated using random-effects meta-analysis. Sample sizes for each dataset provided in Table 2.

## Data Availability

This study makes use of anonymized data held in the Secure Anonymised Information Linkage (SAIL) Databank. We would like to acknowledge all the data providers who made anonymized data available for research (listed in Supplementary Table 1). The responsibility for the interpretation of the information SAIL supplied is the authors’ alone. Data may be available to researchers for analysis after securing relevant permissions from the data contributors and the databank in which the data are held (SAIL Databank). The approvals process is managed by application to the SAIL Databank who hold data sharing agreements with the data providers. Restricted datasets may require additional approvals from data custodians and ethical authorities in the relevant country/setting. Enquiries for data access should be made using the contact form at https://saildatabank.com/contact, or by making an enquiry to ICODA at https://icoda-research.org/contact/.

## Code availability

Custom code that supports the findings of this study is available from the corresponding author Sarah Stock (sarah.stock@ed.ac.uk) upon request.

## References

[1] Chawanpaiboon, S. et al. Global, regional, and national estimates of levels of preterm birth in 2014: a systematic review and modelling analysis. *Lancet Glob. Health* **7**, e37–e46 (2019). 30389451 10.1016/S2214-109X(18)30451-0 PMC6293055

[2] Vogel, J. P. et al. The global epidemiology of preterm birth. *Best. Pract. Res. Clin. Obstet. Gynaecol.* **52**, 3–12 (2018). 29779863 10.1016/j.bpobgyn.2018.04.003

[3] Blencowe, H. et al. National, regional, and worldwide estimates of stillbirth rates in 2015, with trends from 2000: a systematic analysis. *Lancet Glob. Health* **4**, e98–e108 (2016). 26795602 10.1016/S2214-109X(15)00275-2

[4] Hug, L. et al. Global, regional, and national estimates and trends in stillbirths from 2000 to 2019: a systematic assessment. *Lancet* **398**, 772–785 (2021). 34454675 10.1016/S0140-6736(21)01112-0 PMC8417352

[5] Matheson, A. et al. Prematurity rates during the coronavirus disease 2019 (COVID-19) pandemic lockdown in Melbourne, Australia. *Obstet. Gynecol.* **137**, 405–407 (2021). 33543904 10.1097/AOG.0000000000004236 PMC7884082

[6] Gallo, L. A. et al. A decline in planned, but not spontaneous, preterm birth rates in a large Australian tertiary maternity centre during COVID-19 mitigation measures. *Aust. N. Z. J. Obstet. Gynaecol.*10.1111/ajo.13406 (2021). 34254286 10.1111/ajo.13406 PMC8441865

[7] Justman, N. et al. Lockdown with a price: the impact of the COVID-19 pandemic on prenatal care and perinatal outcomes in a tertiary care center. *Isr. Med. Assoc. J.* **22**, 533–537 (2020). 33236549

[8] Hedermann, G. et al. Danish premature birth rates during the COVID-19 lockdown. *Arch. Dis. Child. Fetal Neonatal Ed.* **106**, 93–95 (2020). 32788391 10.1136/archdischild-2020-319990 PMC7421710

[9] McDonnell, S., McNamee, E., Lindow, S. W. & O’Connell, M. P. The impact of the Covid-19 pandemic on maternity services: a review of maternal and neonatal outcomes before, during and after the pandemic. *Eur. J. Obstet. Gynecol. Reprod. Biol.* **255**, 172–176 (2020). 33142263 10.1016/j.ejogrb.2020.10.023 PMC7550066

[10] Been, J. V. et al. Impact of COVID-19 mitigation measures on the incidence of preterm birth: a national quasi-experimental study. *Lancet Public Health* **5**, e604–e611 (2020). 33065022 10.1016/S2468-2667(20)30223-1 PMC7553867

[11] Philip, R. K. et al. Unprecedented reduction in births of very low birthweight (VLBW) and extremely low birthweight (ELBW) infants during the COVID-19 lockdown in Ireland: a ‘natural experiment’ allowing analysis of data from the prior two decades. *BMJ Glob. Health* **5**, e003075 (2020). 32999054 10.1136/bmjgh-2020-003075 PMC7528371

[12] De Curtis, M., Villani, L. & Polo, A. Increase of stillbirth and decrease of late preterm infants during the COVID-19 pandemic lockdown. *Arch. Dis. Child. Fetal Neonatal Ed.*10.1136/archdischild-2020-320682 (2020). 10.1136/archdischild-2020-320682 PMC8237197 33127736

[13] Einarsdóttir, K., Swift, E. M. & Zoega, H. Changes in obstetric interventions and preterm birth during COVID-19: a nationwide study from Iceland. *Acta Obstet. Gynecol. Scand.* **100**, 1924–1930 (2021). 34255860 10.1111/aogs.14231 PMC8444658

[14] Kc, A. et al. Effect of the COVID-19 pandemic response on intrapartum care, stillbirth, and neonatal mortality outcomes in Nepal: a prospective observational study. *Lancet Glob. Health* **8**, e1273–e1281 (2020). 32791117 10.1016/S2214-109X(20)30345-4 PMC7417164

[15] Briozzo, L., Tomasso, G., Viroga, S., Nozar, F. & Bianchi, A. Impact of mitigation measures against the COVID 19 pandemic on the perinatal results of the reference maternity hospital in Uruguay. *J. Matern. Fetal. Neonatal Med*. **35**, 5060–5062 (2021). 10.1080/14767058.2021.1874911 33455516

[16] Main, E. K. et al. Singleton preterm birth rates for racial and ethnic groups during the coronavirus disease 2019 pandemic in California. *Am. J. Obstet. Gynecol.* **224**, 239–241 (2020). 33203528 10.1016/j.ajog.2020.10.033 PMC7582039

[17] Wood, R. et al. Preterm birth during the coronavirus disease 2019 (COVID-19) pandemic in a large hospital system in the United States. *Obstet. Gynecol.* **137**, 403–404 (2021). 33595244 10.1097/AOG.0000000000004237 PMC7884087

[18] Arnaez, J. et al. Lack of changes in preterm delivery and stillbirths during COVID-19 lockdown in a European region. *Eur. J. Pediatr*. **180**, 1997–2002 (2021). 10.1007/s00431-021-03984-6 PMC7880019 33580293

[19] Pasternak, B. et al. Preterm birth and stillbirth during the COVID-19 pandemic in Sweden: a nationwide cohort study. *Ann. Intern. Med.*10.7326/m20-6367 (2021). 33428442 10.7326/M20-6367 PMC7808327

[20] Riley, T., Nethery, E., Chung, E. K. & Souter, V. Impact of the COVID-19 pandemic on perinatal care and outcomes in the United States: an interrupted time series analysis. *Birth*10.1111/birt.12606 (2021). 34957595 10.1111/birt.12606

[21] Sun, S., Savitz, D. A. & Wellenius, G. A. Changes in adverse pregnancy outcomes associated with the COVID-19 pandemic in the United States. *JAMA Netw. Open* **4**, e2129560 (2021). 34652449 10.1001/jamanetworkopen.2021.29560 PMC8520131

[22] Liu, S. et al. Pregnancy outcomes during the COVID-19 pandemic in Canada, March to August 2020. *J. Obstet. Gynaecol. Can.* **43**, 1406–1415 (2021). 34332116 10.1016/j.jogc.2021.06.014

[23] Khalil, A. et al. Change in the incidence of stillbirth and preterm delivery during the COVID-19 pandemic. *JAMA*10.1001/jama.2020.12746 (2020). 32648892 10.1001/jama.2020.12746 PMC7435343

[24] Okeke, E. N., Abubakar, I. S. & De Guttry, R. In Nigeria, stillbirths and newborn deaths increased during the COVID-19 pandemic. *Health Aff.*10.1377/hlthaff.2021.00659 (2021). 10.1377/hlthaff.2021.00659 PMC12501791 34669501

[25] Vaccaro, C., Mahmoud, F., Aboulatta, L., Aloud, B. & Eltonsy, S. The impact of COVID-19 first wave national lockdowns on perinatal outcomes: a rapid review and meta-analysis. *BMC Pregnancy Childbirth* **21**, 676 (2021). 34615505 10.1186/s12884-021-04156-y PMC8532086

[26] Yang, J. et al. COVID-19 pandemic and population-level pregnancy and neonatal outcomes: a living systematic review and meta-analysis. *Acta Obstet. Gynecol. Scand.* **100**, 1756–1770 (2021). 34096034 10.1111/aogs.14206 PMC8222877

[27] Chmielewska, B. et al. Effects of the COVID-19 pandemic on maternal and perinatal outcomes: a systematic review and meta-analysis. *Lancet Glob. Health* **9**, e759–e772 (2021). 33811827 10.1016/S2214-109X(21)00079-6 PMC8012052

[28] Ochoa, L. B., Brockway, M., Stock, S. J. & Been, J. V. COVID-19 and maternal and perinatal outcomes. *Lancet Glob. Health* **9**, e1063–e1064 (2021). 34297958 10.1016/S2214-109X(21)00295-3 PMC8293948

[29] Chiesa, V., Antony, G., Wismar, M. & Rechel, B. COVID-19 pandemic: health impact of staying at home, social distancing and ‘lockdown’ measures-a systematic review of systematic reviews. *J. Public Health* **43**, e462–e481 (2021). 10.1093/pubmed/fdab102 PMC8083256 33855434

[30] Goldenberg, R. L., Culhane, J. F., Iams, J. D. & Romero, R. Epidemiology and causes of preterm birth. *Lancet* **371**, 75–84 (2008). 18177778 10.1016/S0140-6736(08)60074-4 PMC7134569

[31] Todd, I. M. F., Miller, J. E., Rowe, S. L., Burgner, D. P. & Sullivan, S. G. Changes in infection-related hospitalizations in children following pandemic restrictions: an interrupted time-series analysis of total population data. *Int. J. Epidemiol.* **50**, 1435–1443 (2021). 34056664 10.1093/ije/dyab101 PMC8195105

[32] Jones, N. How COVID-19 is changing the cold and flu season. *Nature* **588**, 388–390 (2020). 33324005 10.1038/d41586-020-03519-3

[33] Stieb, D. M., Chen, L., Eshoul, M. & Judek, S. Ambient air pollution, birth weight and preterm birth: a systematic review and meta-analysis. *Environ. Res.* **117**, 100–111 (2012). 22726801 10.1016/j.envres.2012.05.007

[34] Ju, L. et al. Maternal air pollution exposure increases the risk of preterm birth: evidence from the meta-analysis of cohort studies. *Environ. Res.* **202**, 111654 (2021). 34252430 10.1016/j.envres.2021.111654

[35] Venter, Z. S., Aunan, K., Chowdhury, S. & Lelieveld, J. COVID-19 lockdowns cause global air pollution declines. *Proc. Natl Acad. Sci. USA* **117**, 18984–18990 (2020). 32723816 10.1073/pnas.2006853117 PMC7430997

[36] Sarmadi, M., Rahimi, S., Rezaei, M., Sanaei, D. & Dianatinasab, M. Air quality index variation before and after the onset of COVID-19 pandemic: a comprehensive study on 87 capital, industrial and polluted cities of the world. *Environ. Sci. Eur.* **33**, 134 (2021). 34900511 10.1186/s12302-021-00575-y PMC8645297

[37] Kotlar, B., Gerson, E., Petrillo, S., Langer, A. & Tiemeier, H. The impact of the COVID-19 pandemic on maternal and perinatal health: a scoping review. *Reprod. Health* **18**, 10 (2021). 33461593 10.1186/s12978-021-01070-6 PMC7812564

[38] Hale, T., et al. Oxford COVID-19 Government Response Tracker. *Blavatnik School of Government* www.bsg.ox.ac.uk/covidtracker (2020).

[39] Ashorn, P. et al. The Lancet Small Vulnerable Newborn Series: science for a healthy start. *Lancet* **396**, 743–745 (2020). 32919498 10.1016/S0140-6736(20)31906-1

[40] Kramer, M. S., Zhang, X. & Platt, R. W. Analyzing risks of adverse pregnancy outcomes. *Am. J. Epidemiol.* **179**, 361–367 (2014). 24287468 10.1093/aje/kwt285

[41] Ananth, C. V. & Vintzileos, A. M. Epidemiology of preterm birth and its clinical subtypes. *J. Matern. Fetal Neonatal Med.* **19**, 773–782 (2006). 17190687 10.1080/14767050600965882

[42] Cuestas, E. et al. Association between COVID-19 mandatory lockdown and decreased incidence of preterm births and neonatal mortality. *J. Perinatol.* **41**, 2566–2569 (2021). 34050246 10.1038/s41372-021-01116-4 PMC8162487

[43] Khalil, A. et al. Change in obstetric attendance and activities during the COVID-19 pandemic. *Lancet Infect. Dis.*10.1016/s1473-3099(20)30779-9 (2020). 33031754 10.1016/S1473-3099(20)30779-9 PMC7535627

[44] Allotey, J. et al. Clinical manifestations, risk factors, and maternal and perinatal outcomes of coronavirus disease 2019 in pregnancy: living systematic review and meta-analysis. *Brit. Med. J.* **370**, m3320 (2020). 32873575 10.1136/bmj.m3320 PMC7459193

[45] Villar, J. et al. Maternal and neonatal morbidity and mortality among pregnant women with and without COVID-19 infection: The INTERCOVID Multinational Cohort Study. *JAMA Pediatr.* **175**, 817–826 (2021). 33885740 10.1001/jamapediatrics.2021.1050 PMC8063132

[46] Stock, S. J. et al. SARS-CoV-2 infection and COVID-19 vaccination rates in pregnant women in Scotland. *Nat. Med.*10.1038/s41591-021-01666-2 (2022). 35027756 10.1038/s41591-021-01666-2 PMC8938271

[47] Walani, S. R. Global burden of preterm birth. *Int. J. Gynaecol. Obstet.* **150**, 31–33 (2020). 32524596 10.1002/ijgo.13195

[48] von Wissmann, B. et al. Informing prevention of stillbirth and preterm birth in Malawi: development of a minimum dataset for health facilities participating in the DIPLOMATIC collaboration. *BMJ Open* **10**, e038859 (2020). 10.1136/bmjopen-2020-038859 PMC7689106 33234630

[49] World Health Organization & Others. Every newborn: an action plan to end preventable deaths. *WHO, UNICEF* https://apps.who.int/iris/bitstream/handle/10665/127938/9789241507448_eng.pdf (2014).

[50] Brabin, P. et al. The International Stillbirth Alliance: connecting for life. *Lancet* **377**, 1313 (2011). 21497691 10.1016/S0140-6736(11)60530-8

[51] Frøen, J. F. et al. Stillbirths: progress and unfinished business. *Lancet* **387**, 574–586 (2016). 26794077 10.1016/S0140-6736(15)00818-1

[52] Homer, C. S. E. et al. Counting stillbirths and COVID 19-there has never been a more urgent time. *Lancet Glob. Health* **9**, e10–e11 (2021). 33212029 10.1016/S2214-109X(20)30456-3 PMC10011432

[53] Lee, S. J., Steer, P. J. & Filippi, V. Seasonal patterns and preterm birth: a systematic review of the literature and an analysis in a London-based cohort. *BJOG* **113**, 1280–1288 (2006). 17120349 10.1111/j.1471-0528.2006.01055.x

[54] Frøen, J. F. et al. eRegistries: Electronic registries for maternal and child health. *BMC Pregnancy Childbirth* **16**, 11 (2016). 26791790 10.1186/s12884-016-0801-7 PMC4721069

[55] Stock, S. J. et al. The international Perinatal Outcomes in the Pandemic (iPOP) study: protocol. *Wellcome Open Res.* **6**, 21 (2021). 34722933 10.12688/wellcomeopenres.16507.1 PMC8524299

[56] von Elm, E. et al. The Strengthening the Reporting of Observational Studies in Epidemiology (STROBE) statement: guidelines for reporting observational studies. *Bull. World Health Organ.* **85**, 867–872 (2007). 18038077 10.2471/BLT.07.045120 PMC2636253

[57] Jones, K. H., Ford, D. V., Thompson, S. & Lyons, R. A. A Profile of the SAIL Databank on the UK Secure Research Platform. *Int. J. Popul. Data Sci.* **4**, 1134 (2019). 34095541 10.23889/ijpds.v4i2.1134 PMC8142954

[58] The World Bank - DataBank *The World Bank* https://databank.worldbank.org/home.aspx (2022).

[59] Lawn, J. E. et al. Global report on preterm birth and stillbirth (1 of 7): definitions, description of the burden and opportunities to improve data. *BMC Pregnancy Childbirth* **10****Suppl 1**, S1 (2010). 10.1186/1471-2393-10-S1-S1 PMC2841772 20233382

[60] World Health Organization *Neonatal and perinatal mortality: country, regional and global estimates* (World Health Organization, 2006).

[61] Quinn, J.-A. et al. Preterm birth: case definition & guidelines for data collection, analysis, and presentation of immunisation safety data. *Vaccine* **34**, 6047–6056 (2016). 27743648 10.1016/j.vaccine.2016.03.045 PMC5139808

[62] Bernal, J. L., Cummins, S. & Gasparrini, A. Interrupted time series regression for the evaluation of public health interventions: a tutorial. *Int. J. Epidemiol.* **46**, 348–355 (2017). 27283160 10.1093/ije/dyw098 PMC5407170

[63] Akaike, H. in *Selected Papers of Hirotugu Akaike* (eds Parzen, E. et al.) 199–213 (Springer, 1998).

[64] DerSimonian, R. & Laird, N. Meta-analysis in clinical trials. *Control. Clin. Trials* **7**, 177–188 (1986). 3802833 10.1016/0197-2456(86)90046-2

[65] Konstantopoulos, S. Fixed effects and variance components estimation in three-level meta-analysis. *Res. Synth. Methods* **2**, 61–76 (2011). 10.1002/jrsm.35 26061600

[66] Higgins, J. P. T., Thompson, S. G., Deeks, J. J. & Altman, D. G. Measuring inconsistency in meta-analyses. *Brit. Med. J.* **327**, 557–560 (2003). 12958120 10.1136/bmj.327.7414.557 PMC192859

