# Supplementary material for: Changes in preterm birth and stillbirth during COVID-19 lockdowns in 26 countries
Source: Nat Hum Behav. 2023 Feb 27;7(4):529–44. doi: 10.1038/s41562-023-01522-y (PMC10129868; doi:10.1038/s41562-023-01522-y)
Supplement: Supplementary file 1 — Supplementary methods, discussion, Figs. 1–44, Tables 1–6 and references. [file 41562_2023_1522_MOESM1_ESM.pdf]

nature human behaviour

Article

https://doi.org/10.1038/s41562-023-01522-y

Changes in preterm birth and stillbirth during COVID-19 lockdowns in 26 countries

In the format provided by the authors and unedited

Table of Contents

Supplementary Methods 2
Supplementary Discussion 5
Supplementary Figures 7
Supplementary Tables 51
Supplementary References 60

Supplementary Methods

Data preparation and management

Suppressed data: For the few datasets where suppressed values were present due to small numbers (<5), these were inputted using two different methods depending on how data providers suppressed the data: (1) for datasets where the total number of births did not equal the total number of non-suppressed cells, we divided the number of non-allocated births by the number of suppressed cells and (2) for datasets where the total number of births equalled the total number of non-suppressed cells, we inputted the suppressed cell as the midpoint between 1 and the threshold for suppression (usually <5 births) and recalculated the total number of births.

Missing and outlier data: The distribution of the number of births with missing information on gestational age was investigated to determine if these data were missing at random with respect to lockdown. If there was no evidence to suggest that data missing was not at random and if the percentage of births missing information on gestational age did not change between the lockdown and pre-lockdown periods, then we assumed that these were missing gestational age completely at random and re-allocated these births proportionally across the gestational age groups. Where data on births were completely missing for a given month, linear interpolation of the outcome rates was performed using data from the 6 nearest surrounding time-points for the population-based data. For the non-population-based data, where there were higher levels of missing data for consecutive months in some of the datasets, we did not

input these values and only modelled using the observed data. We graphed the preterm and stillbirth rates for each month for each dataset to check that the fell within plausible ranges; all plots were reviewed by the statistical analysis team (including clinicians, statisticians and epidemiologists) and where implausible rates were identified, we followed-up with the data provider to check if there had been a data entry error. Where the rates could not be corrected, the implausible data points were treated as missing for analysis.

Bias in capture of births in lockdown: Given the early stage of the pandemic, we would not expect to see any changes in the number of births being observed in our data sources compared to pre-lockdown unless driven by a bias in which women were giving birth in different locations and not being recorded, or due to changes in recording practices. To assess this, we forecasted the expected total numbers of births using a Poisson time series, based on pre-lockdown seasonal and yearly trends, and compared the observed number of births to expected number of births. We calculated the percentage change in the total number of births in the lockdown period by dividing the observed total number of births by the expected number of births. Any population-based datasets where there was a relative change of 10% or more in the number of observed compared to expected births following lockdown were excluded from the population-based analysis, and analyzed as a non-population-based dataset.

Data Management: Data were stored and analyzed in the UK Secure Anonymized Information Linkage (SAIL) Databank[1,2], Swansea Wales, in compliance with the European General Data Protection Regulation guidelines, adhering to the global gold standard of data governance. All data contributors completed a Data Contribution Agreement (DCA) between their institution

and SAIL and were provided with a secure link to upload data directly to the SAIL repository.

To ensure outputs were confidential and safe, all statistical outputs were checked using Statistical Disclosure Control (SDC) procedures before being exported out of the virtual environment. We used SDC guiding principles from the Handbook on SDC for Outputs by the UK Data Service[3]. This prevented the identity of a birth from being revealed or inferred from outputs.

Supplementary Discussion

Patient Partner Interpretation

Behind every statistic, there is a story of a baby and a family. Patient organizations from around the globe were raising awareness about inequalities in the area of maternal and newborn health long before the COVID-19 pandemic. Disparities have existed between countries in the delivery of prenatal care for many years; however, the lack of robust data collection strategies and standardized birth registries have hampered efforts to understand these disparities and gain insight towards the underlying causes of preterm birth. As a patient community, we were optimistic that the iPOP Study findings might help us identify reasons why rates of prematurity and stillbirths may have declined in some countries early in the pandemic and that these ‘reasons’ might be leveraged to help reduce the global preterm birth and stillbirth rates. We perceive two major learnings from the iPOP Study: one related to the study results and another related to the challenges faced by the researchers.

The iPOP Study results revealed small differences in preterm and stillbirth rates during the COVID-19 pandemic, and while the scope of this paper did not identify a reason, we feel it may be due to the impact on access to care. The experience of patient organizations working with families who experience preterm birth indicate that because of pandemic enforced changes to maternal and neonatal care, the patient experience has been dramatically altered[55]. With access to existing care pathways and evidence-based family-centered care severely disrupted, patient organizations have reported increasing numbers of families seeking

alternative sources of support and resources[4]. Our experience leads us to believe that the iPOP Study results are likely related to the significant shift in maternal and newborn care pathways around the globe.

The iPOP Study researchers faced many challenges related to data collection and quality. They had access to limited numbers of globally distributed data sets and obtaining comparable data, especially from LMICs, proved very difficult. These challenges lead us to conclude that maternal and newborn health is still not prioritized as a topic warranting immediate and urgent attention in numerous health systems around the world. GLANCE, the Global Alliance for Newborn Care was launched in 2019 by the European Foundation for the Care of Newborn Infants (EFCNI). Patient organizations from 15 countries contributed towards a Call to Action, advocating for the development of initiatives aimed at improving newborn and maternal health worldwide. Up-to-date, reliable data gathered through standardized methodologies is the cornerstone upon which future research and quality care initiatives must be built and as a collective voice. As such, we are calling for researchers and health providers to learn from the iPOP Study and the pandemic as a whole, to address the deficit in reliable and consistent global maternal and newborn health data.

Supplementary Figures

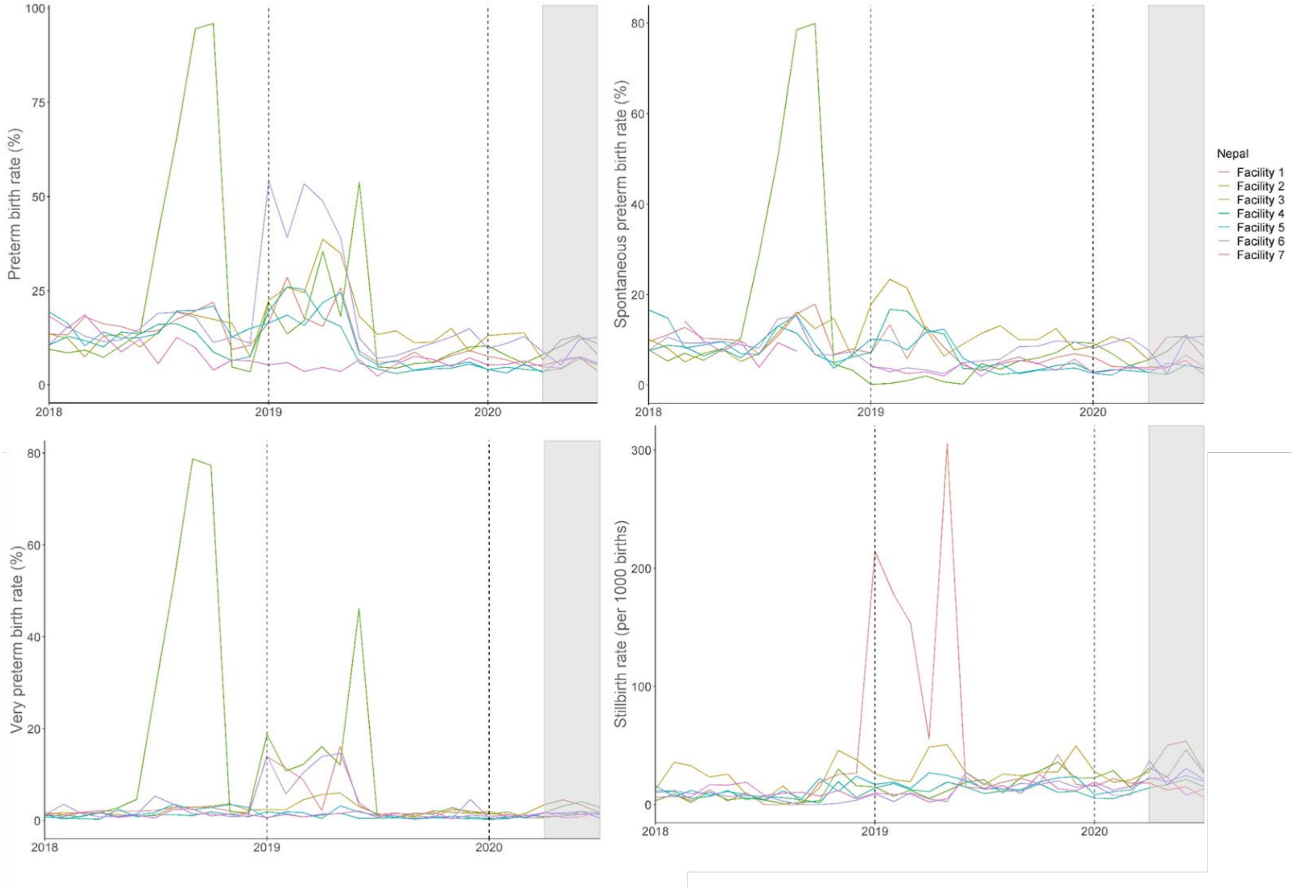

Supplementary Figure 1: Preterm birth rates, stillbirth rates, very preterm birth rates and spontaneous preterm birth rates among all births 22 weeks onwards over time in non-population-based datasets from ***Nepal (excluded)****, stratified by facility.*

Lockdown period shown in shaded grey.

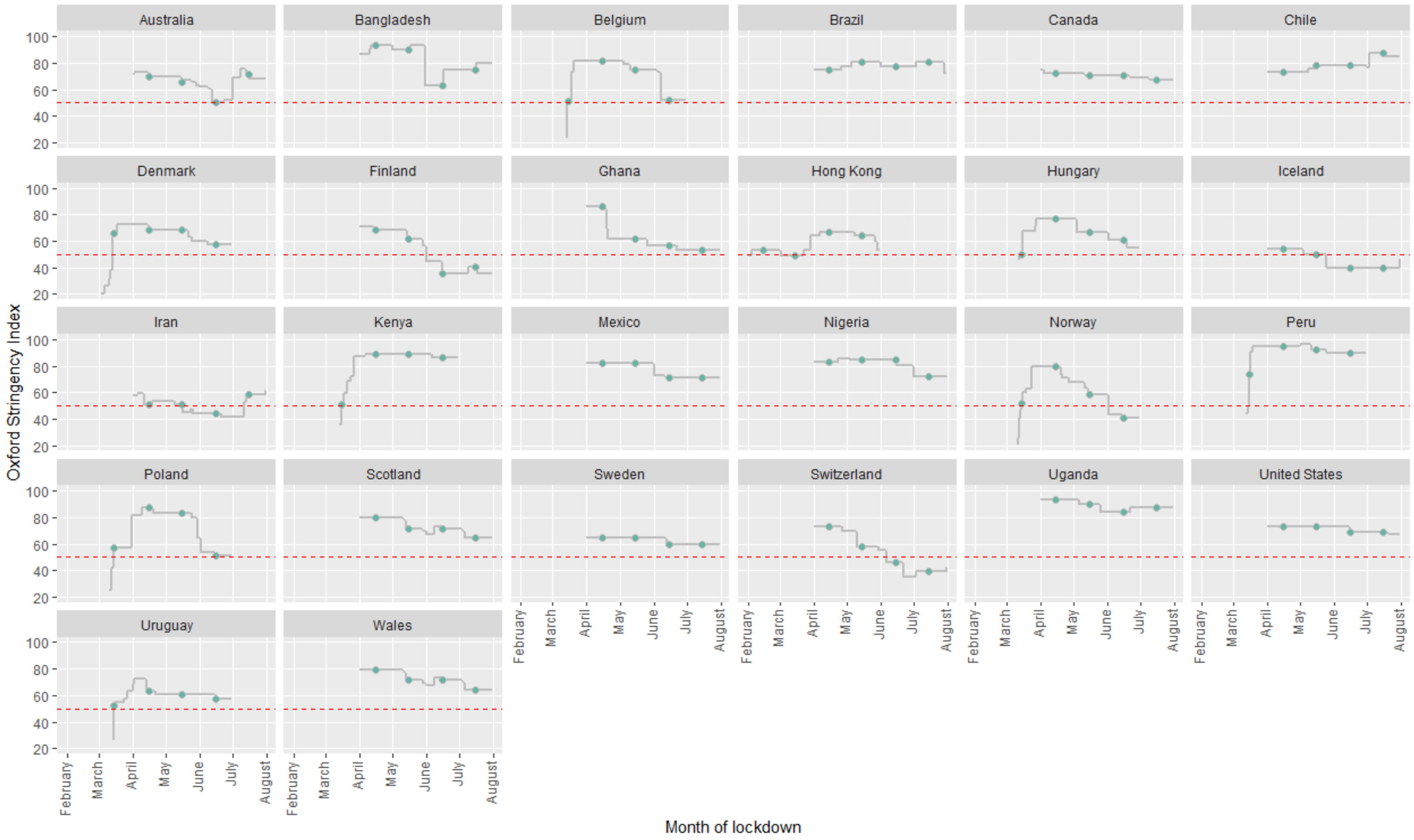

Supplementary Figure 2: ***Change in lockdown stringency over study period among countries included in the iPOP Study****. Change in Oxford lockdown stringency index over lockdown study period, stratified by country.*

Dashed red line shows the stringency index of 50.

Preterm and stillbirth rates over time in non-population-based datasets

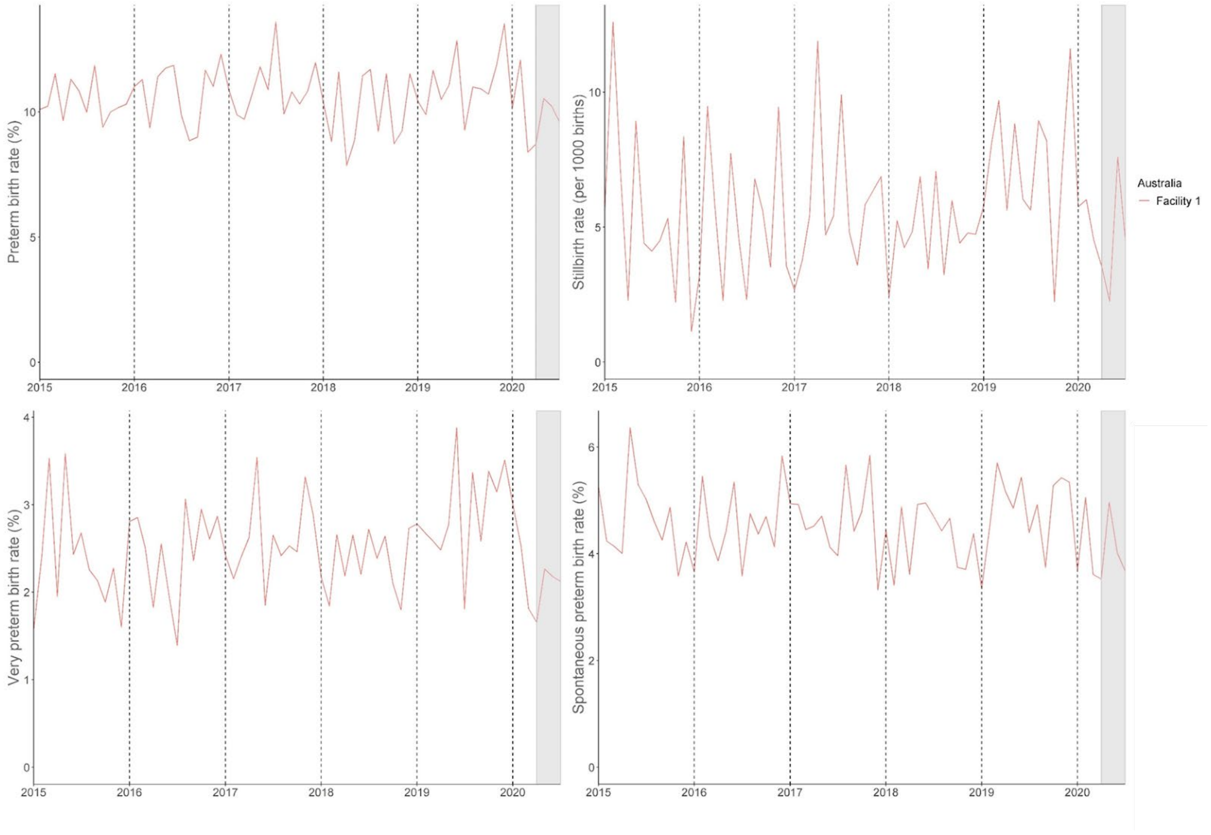

Supplementary Figure 3: Preterm birth rates, stillbirth rates, very preterm birth rates and spontaneous preterm birth rates among all births 22 weeks onwards over time in a non-population-based dataset from ***Queensland, Australia.***

Lockdown period shown in shaded grey.

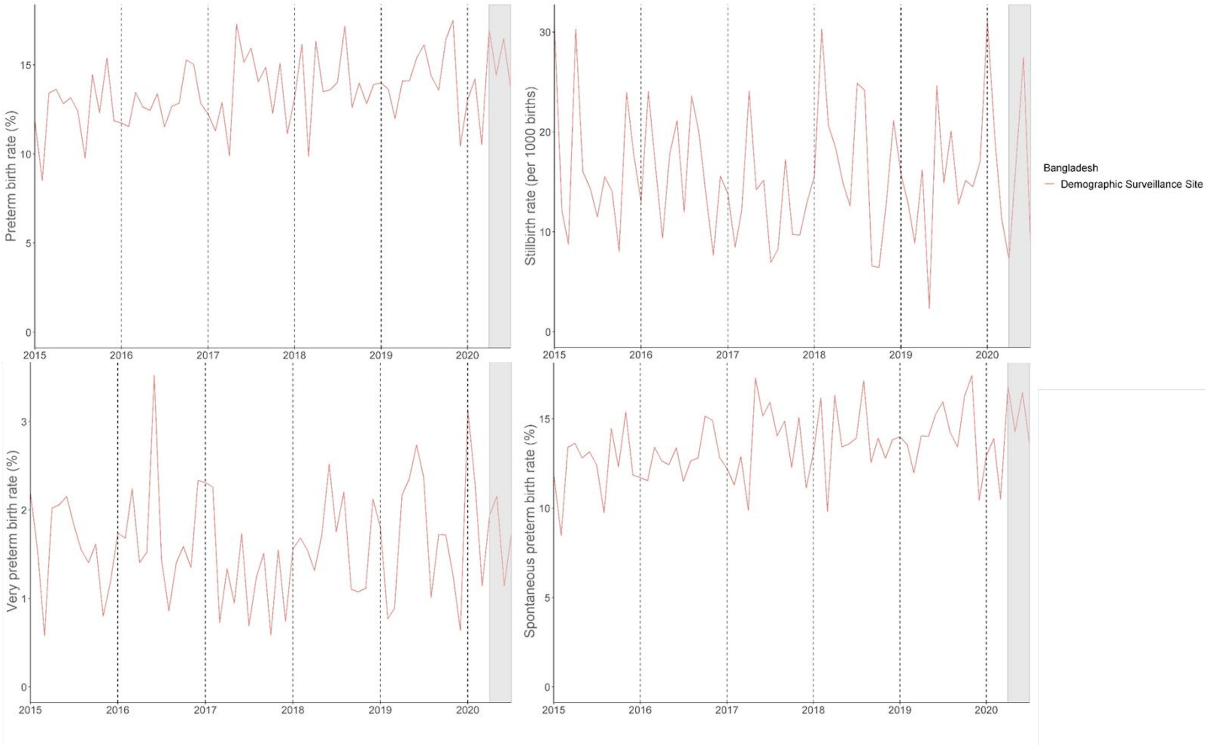

*Supplementary Figure 4: Preterm birth rates, very preterm birth rates and spontaneous preterm birth rates among all births 22 weeks onwards over time in a non-population-based dataset from **Matlab, Bangladesh**.*

Lockdown period shown in shaded grey.

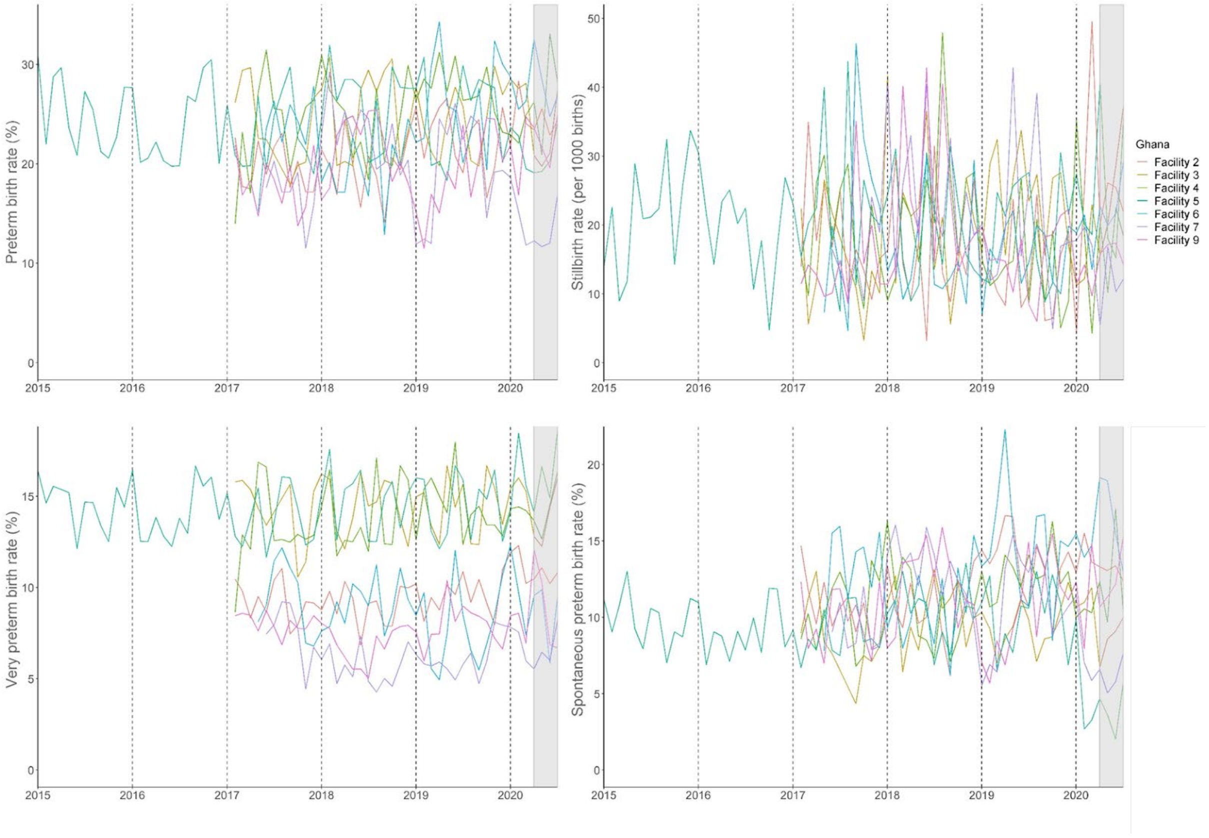

Supplementary Figure 5: Preterm birth rates, stillbirth rates, very preterm birth rates and spontaneous preterm birth rates among all births 22 weeks onwards over time in non-population-based datasets from ***Ghana****, stratified by facility.*

Lockdown period shown in shaded grey.

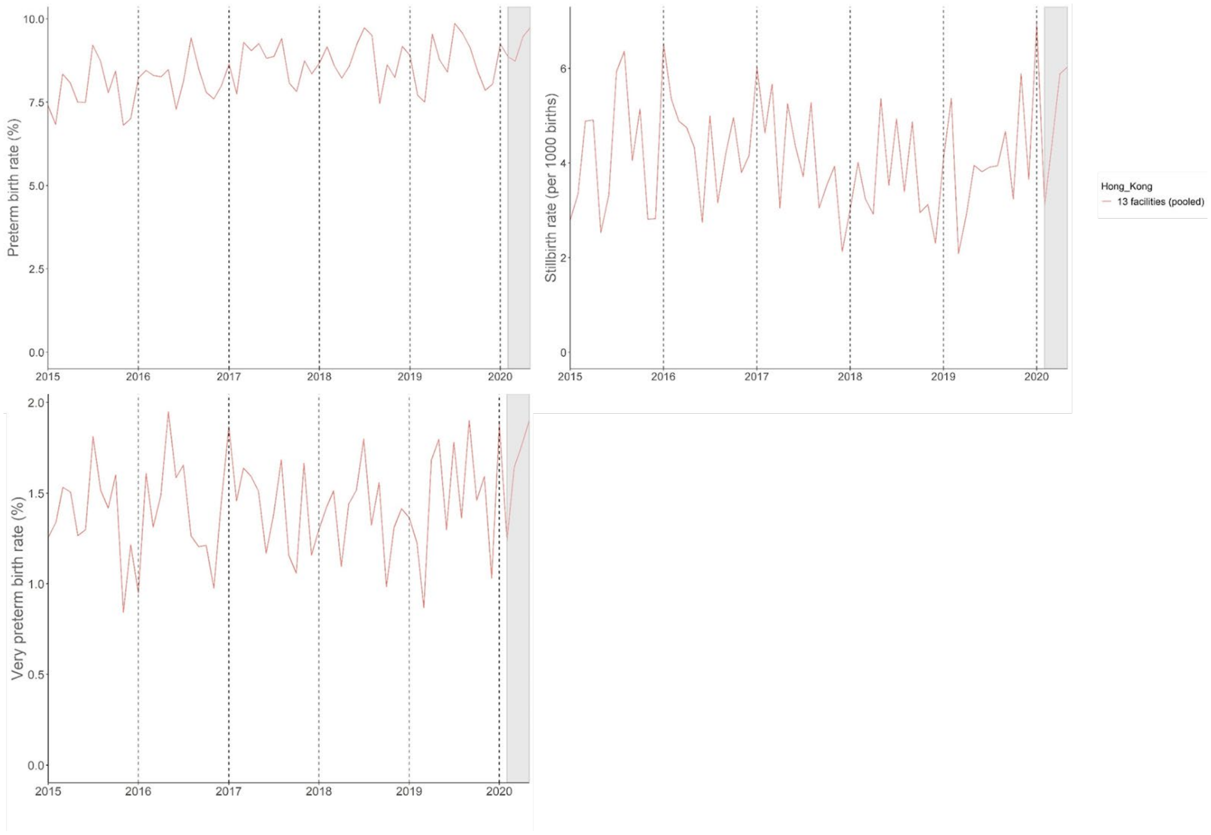

Supplementary Figure 6: Preterm birth rates, stillbirth rate and very preterm birth rates among all births 22 weeks onwards over time in a non-population-based dataset from ***Hong Kong****.*

Lockdown period shown in shaded grey.

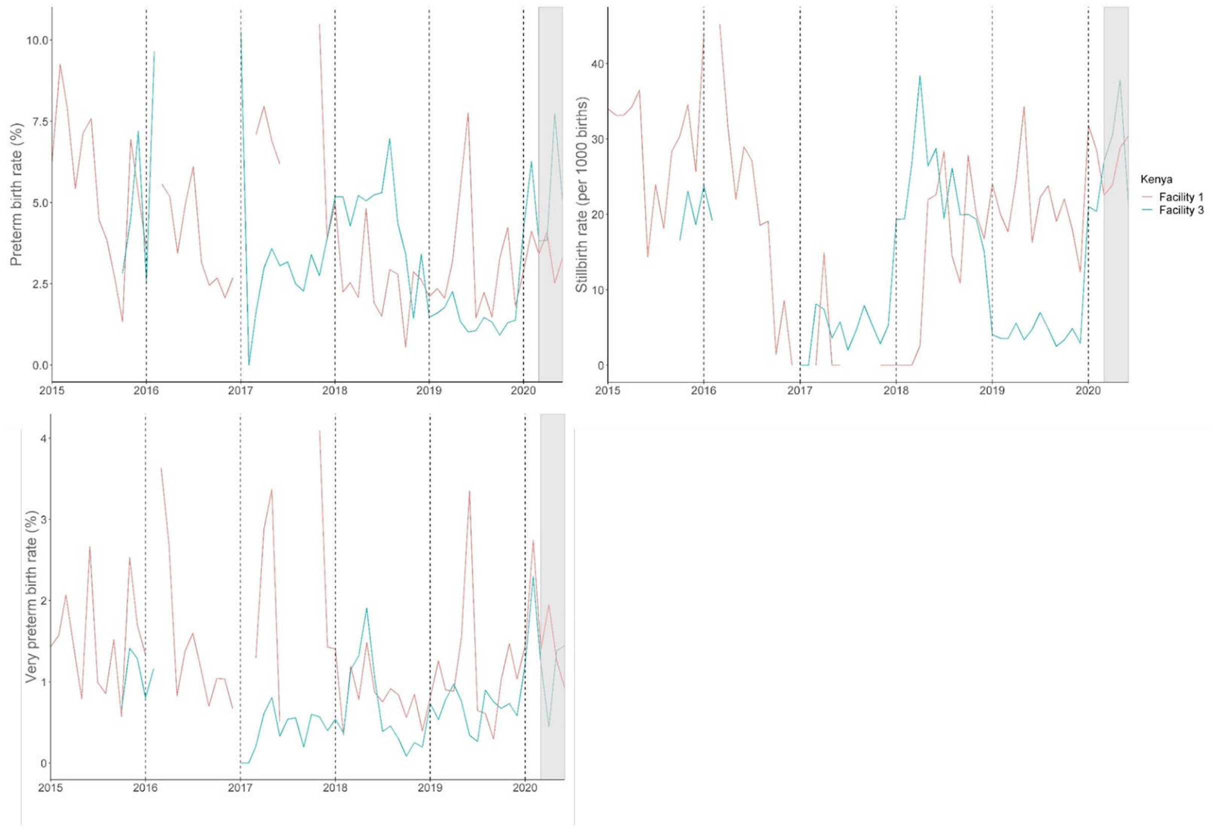

Supplementary Figure 7: Preterm birth rates, stillbirth rate and very preterm birth rates among all births 22 weeks onwards over time in non-population-based datasets from ***Kenya****, stratified by facility.*

Lockdown period shown in shaded grey.

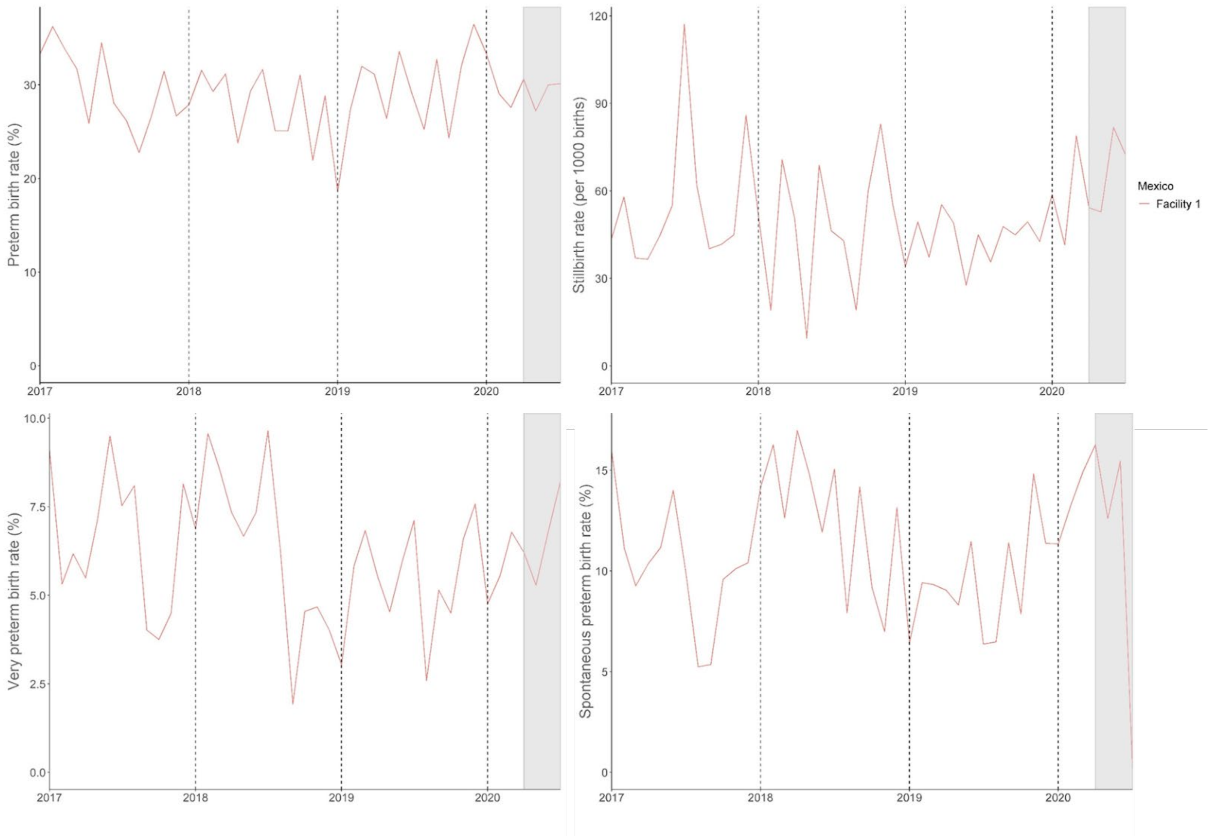

*Supplementary Figure 8: Preterm birth rates, stillbirth rates, very preterm birth rates and spontaneous preterm birth rates among all births 22 weeks onwards over time in a non-population-based dataset from **Mexico**.*

Lockdown period shown in shaded grey.

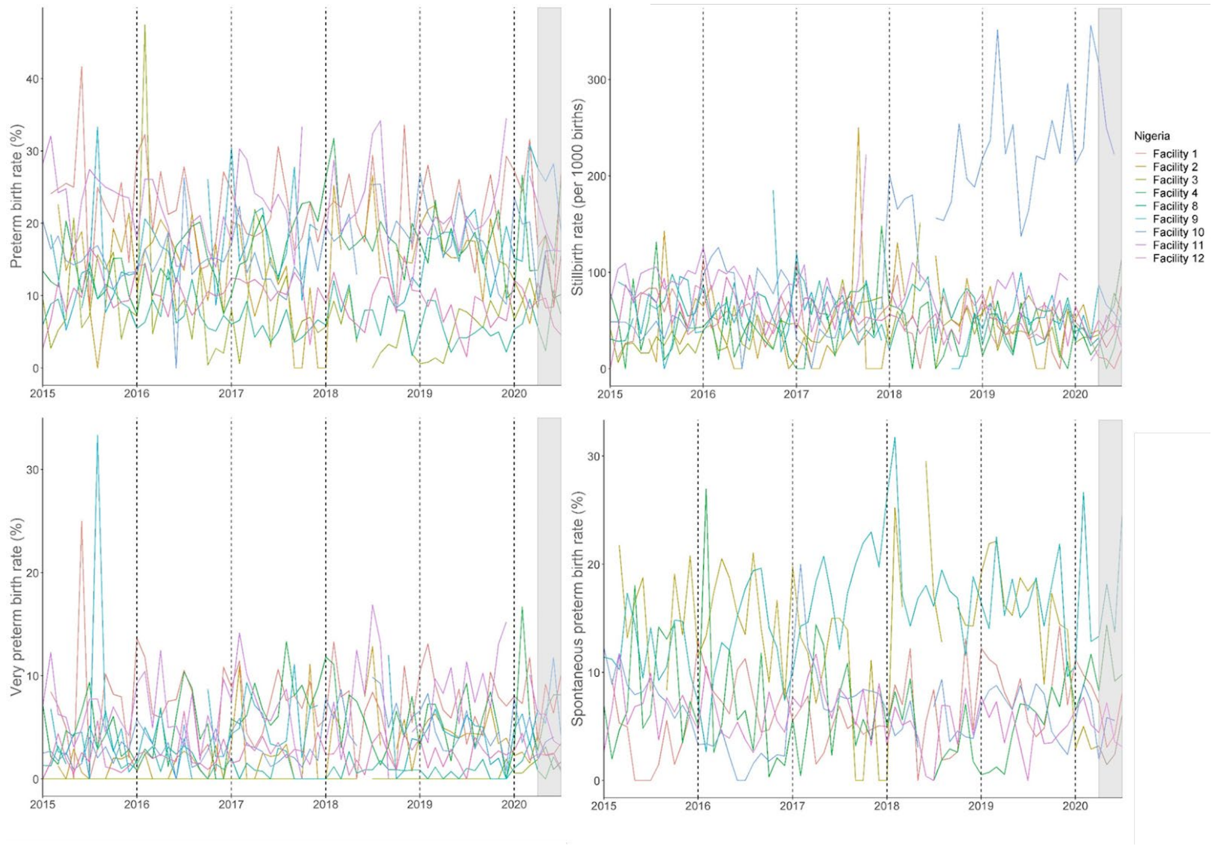

*Supplementary Figure 9: Preterm birth rates, stillbirth rates, very preterm birth rates and spontaneous preterm birth rates among all births 22 weeks onwards over time in non-population-based datasets from **Nigeria**, stratified by facility.*

Lockdown period shown in shaded grey.

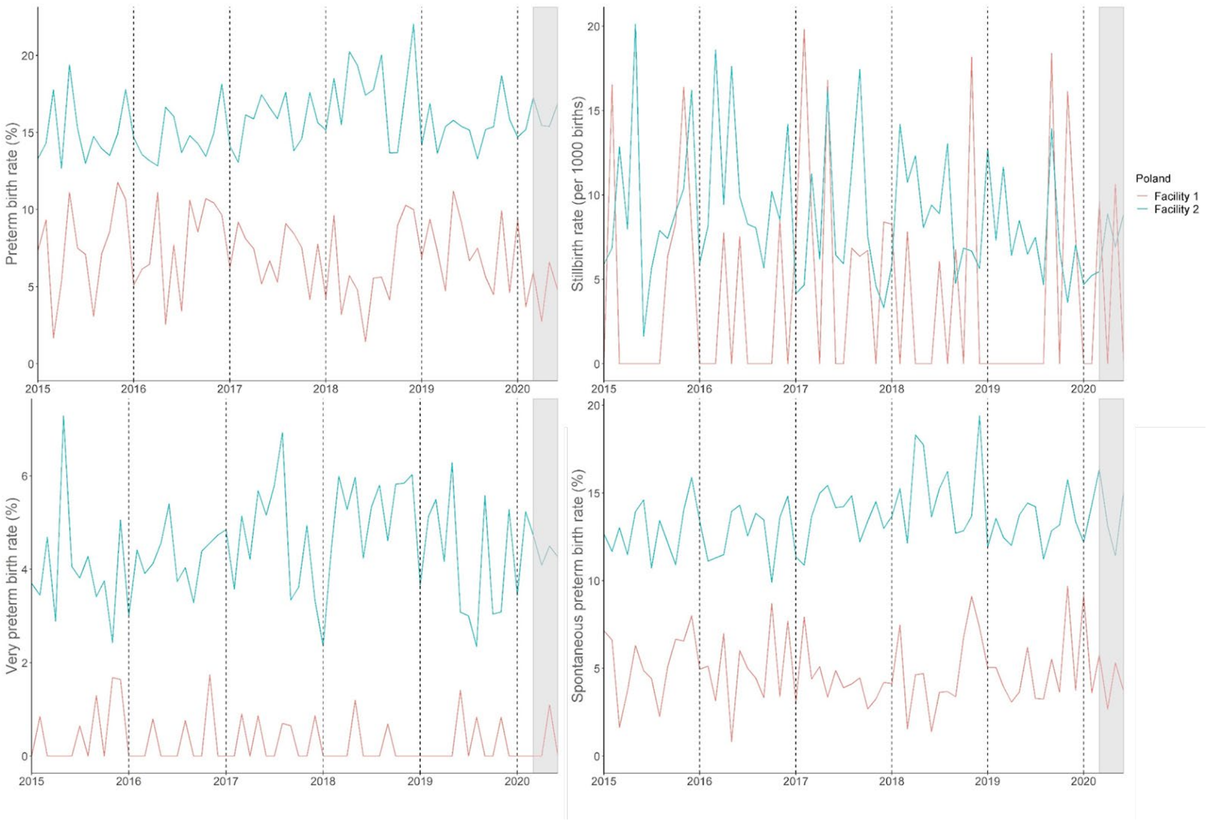

*Supplementary Figure 10: Preterm birth rates, stillbirth rates, very preterm birth rates and spontaneous preterm birth rates among all births 22 weeks onwards over time in non-population-based datasets from **Poland**, stratified by facility.*

Lockdown period shown in shaded grey.

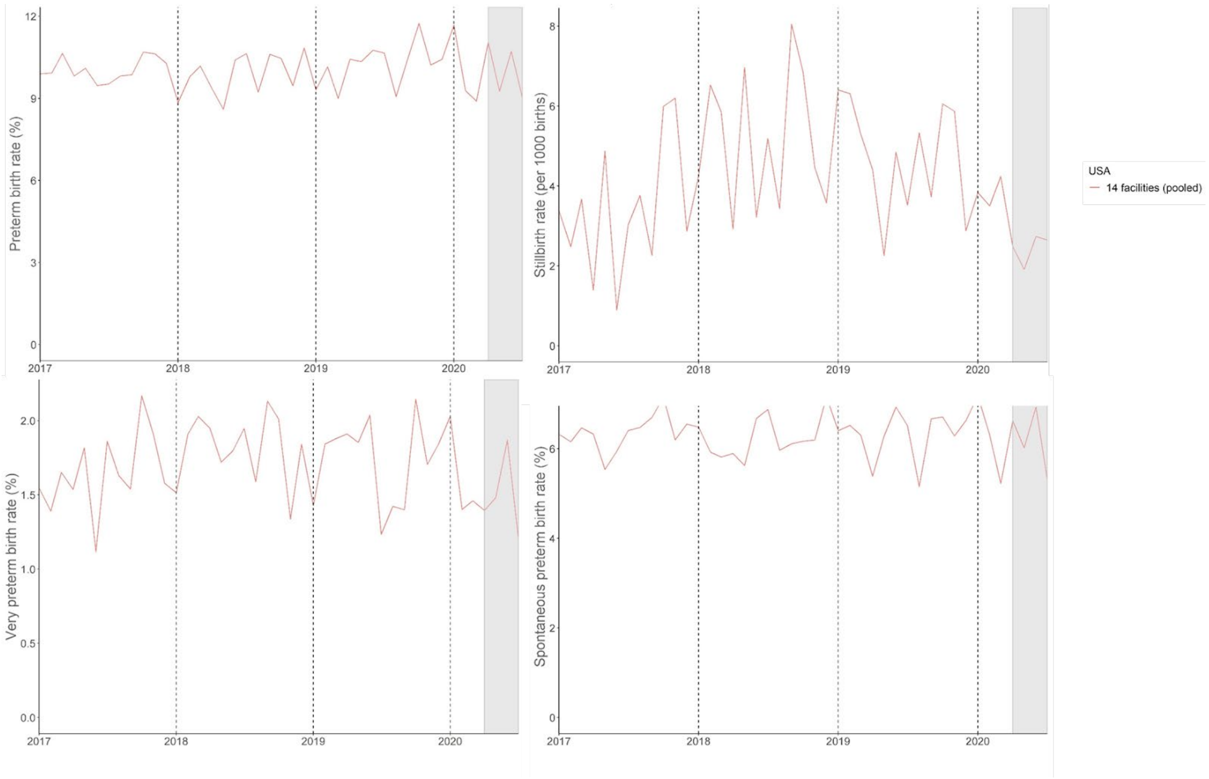

*Supplementary Figure 11: Preterm birth rates, stillbirth rates, very preterm birth rates and spontaneous preterm birth rates among all births 22 weeks onwards over time in a non-population-based dataset from **Washington State, USA**.*

Lockdown period shown in shaded grey.

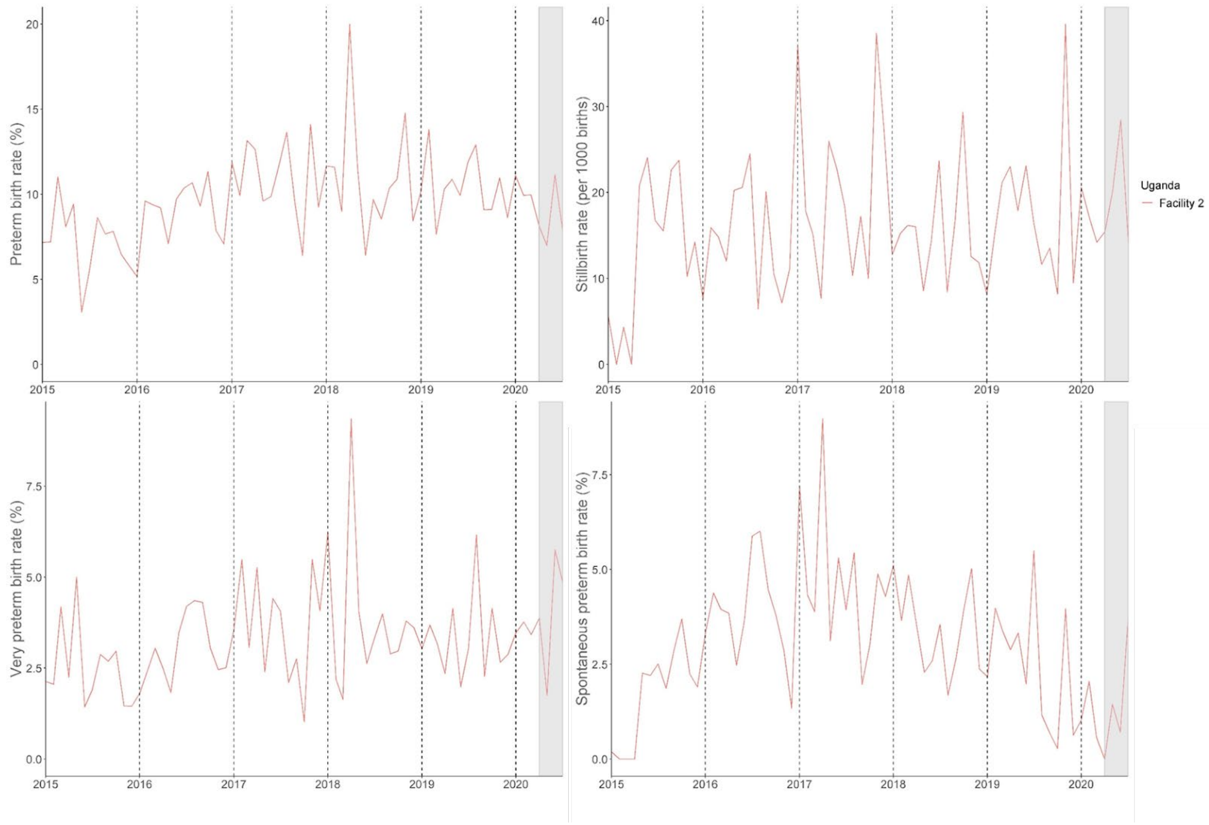

Supplementary Figure 12: Preterm birth rates, stillbirth rates, very preterm birth rates and spontaneous preterm birth rates among all births 22 weeks onwards over time in non-population-based datasets from ***Uganda****.*

Lockdown period shown in shaded grey.

Association between lockdown and preterm birth rates, by time since lockdown

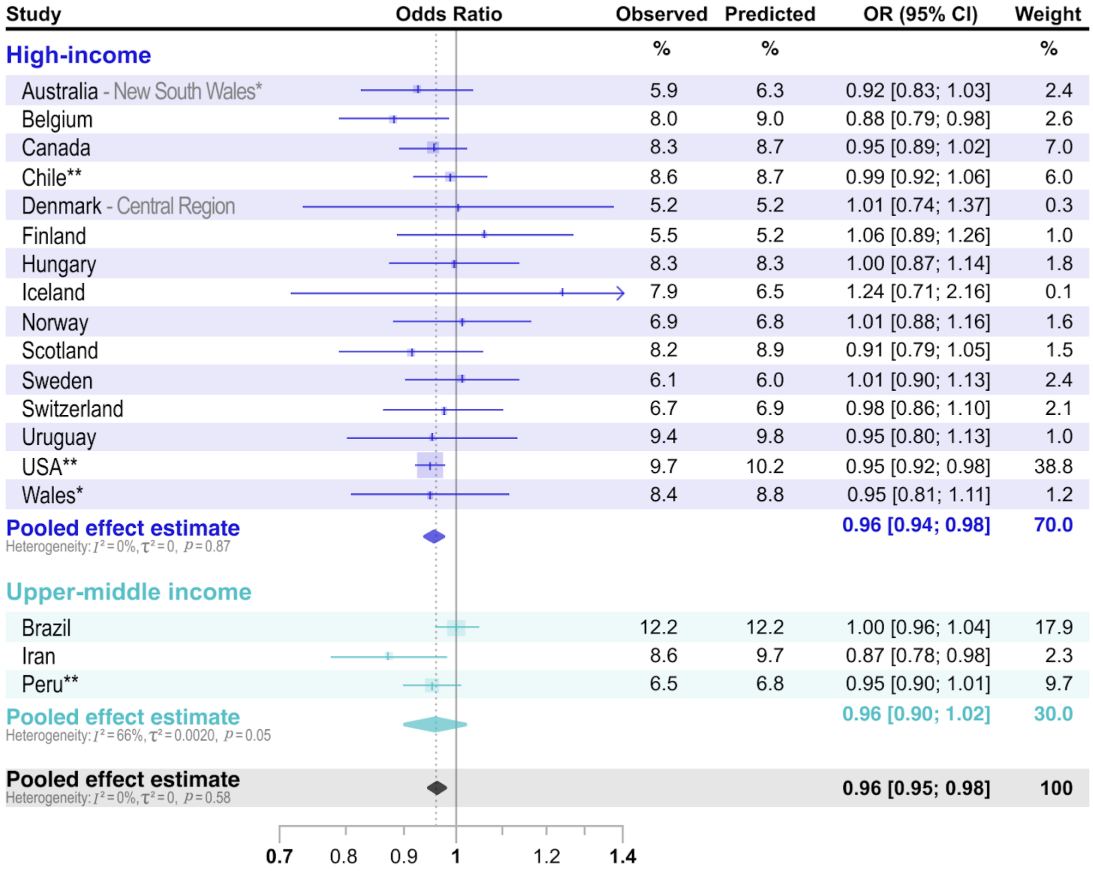

Supplementary Figure 13: Individual and pooled ***population-based estimates*** *of the association between lockdown and the odds of* ***preterm birth*** *among all births 22 weeks onwards, in the* ***first month*** *of lockdown. Individual country odds ratios (represented by boxes on plot) were calculated by comparing the observed odds of preterm birth in the first month of lockdown to the forecasted odds of preterm birth in the first month of lockdown from an interrupted time series model that was fitted to pre-lockdown data. Horizontal lines surrounding each box on the forest plot are 95% confidence intervals. Arrows indicate upper and or lower bounds of the confidence interval that are outside the x-axis limits. *Births from 24 weeks onwards; **Live births only*

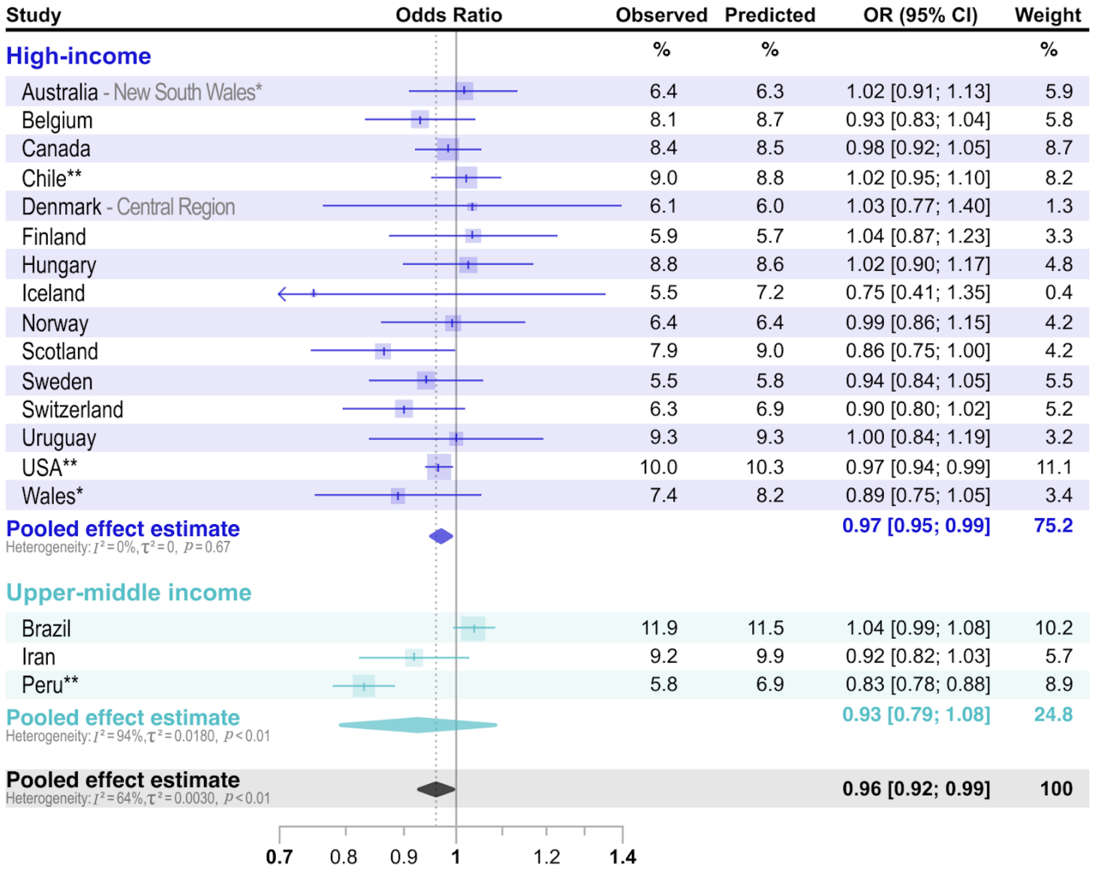

*Supplementary Figure 14: Individual and pooled **population-based estimates** of the association between lockdown and the odds of **preterm birth** among all births 22 weeks onwards, in the **second month** of lockdown. Individual country odds ratios (represented by boxes on plot) were calculated by comparing the observed odds of preterm birth in the second month of lockdown to the forecasted odds of preterm birth in the second month of lockdown from an interrupted time series model that was fitted to pre-lockdown data. Horizontal lines surrounding each box on the forest plot are 95% confidence intervals. Arrows indicate upper and or lower bounds of the confidence interval that are outside the x-axis limits. *Births from 24 weeks onwards; **Live births only*

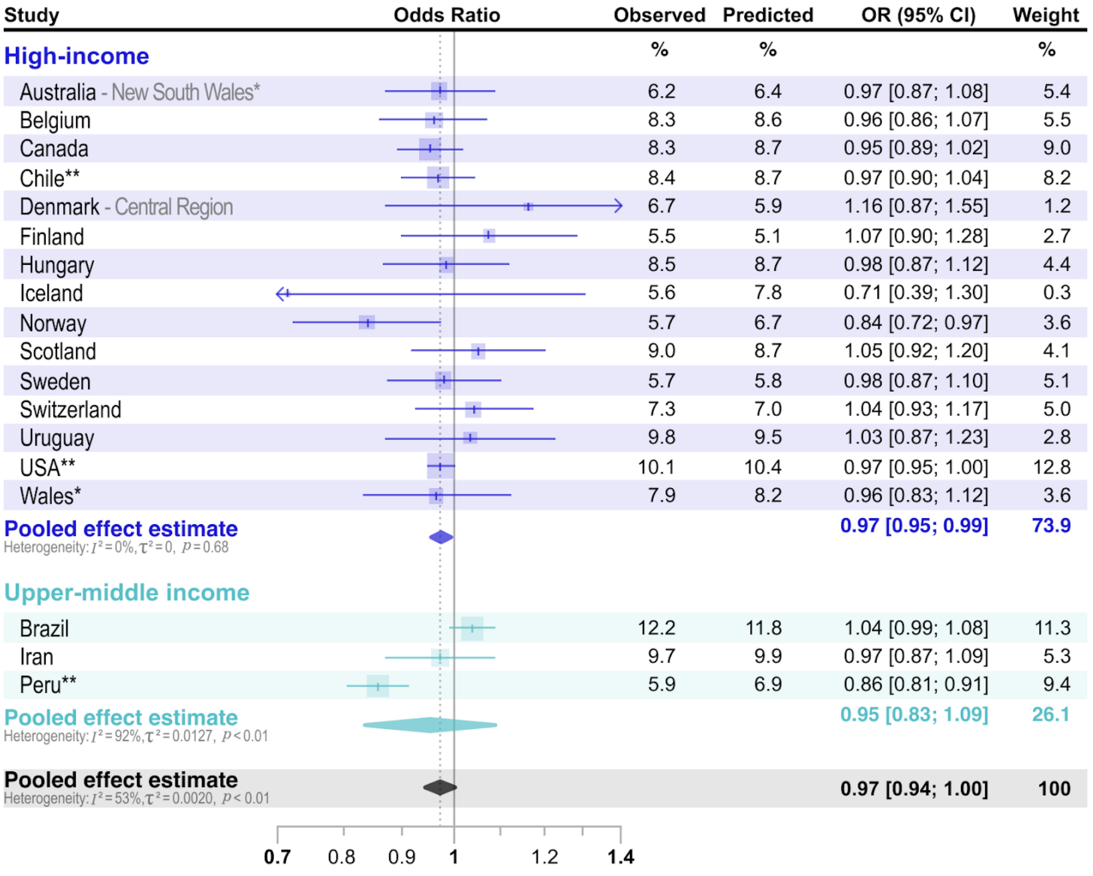

Supplementary Figure 15: Individual and pooled ***population-based estimates*** *of the association between lockdown and the odds of* ***preterm birth*** *among all births 22 weeks onwards, in the* ***third month*** *of lockdown. Individual country odds ratios (represented by boxes on plot) were calculated by comparing the observed odds of preterm birth in the third month of lockdown to the forecasted odds of preterm birth in the third month of lockdown from an interrupted time series model that was fitted to pre-lockdown data. Horizontal lines surrounding each box on the forest plot are 95% confidence intervals. Arrows indicate upper and or lower bounds of the confidence interval that are outside the x-axis limits. *Births from 24 weeks onwards; **Live births only*

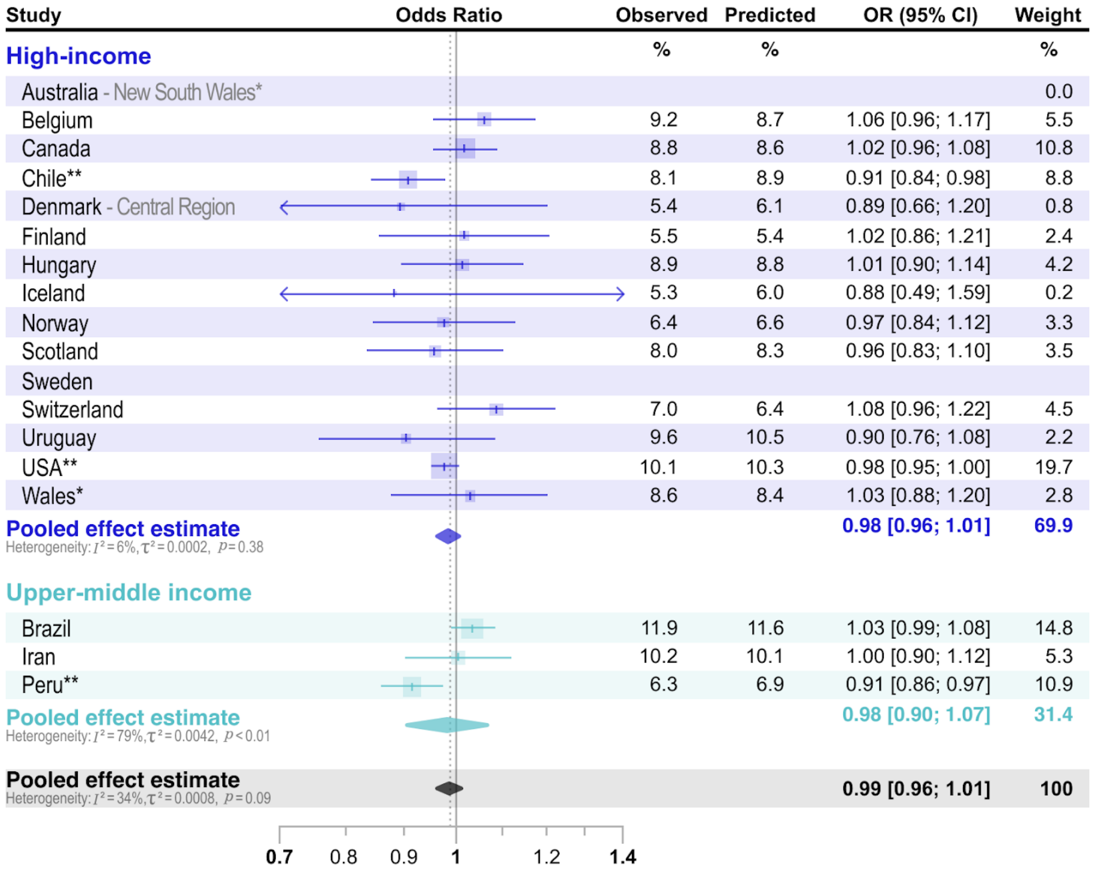

*Supplementary Figure 16: Individual and pooled **population-based estimates** of the association between lockdown and the odds of **preterm birth** among all births 22 weeks onwards, in the **fourth month** of lockdown. Individual country odds ratios (represented by boxes on plot) were calculated by comparing the observed odds of preterm birth in the fourth month of lockdown to the forecasted odds of preterm birth in the fourth month of lockdown from an interrupted time series model that was fitted to pre-lockdown data. Horizontal lines surrounding each box on the forest plot are 95% confidence intervals. Arrows indicate upper and or lower bounds of the confidence interval that are outside the x-axis limits. *Births from 24 weeks onwards; **Live births only*

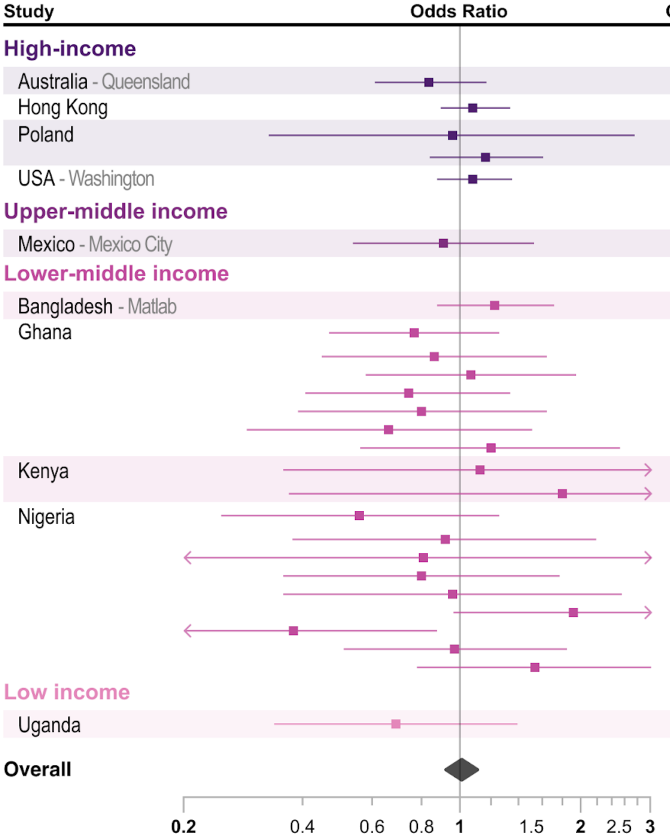

Study	Observed %	Predicted %	OR (95% CI)	Weight %
**High-income**				
Australia - Queensland	8.8	10.2	0.84 (0.61-1.16)	8.4
Hong Kong	8.9	8.2	1.09 (0.90-1.33)	22.8
Poland	6.2	5.5	0.95 (0.33-2.72)	0.8
	17.3	15.2	1.16 (0.84-1.60)	8.2
USA - Washington	11.1	10.3	1.09 (0.88-1.34)	18.9
**Upper-middle income**				
Mexico - Mexico City	30.8	32.8	0.91 (0.54-1.52)	3.2
**Lower-middle income**				
Bangladesh - Matlab	17.1	14.2	1.23 (0.88-1.72)	7.8
Ghana	23.5	28.0	0.77 (0.47-1.25)	3.7
	21.0	23.6	0.86 (0.45-1.64)	2.1
	26.4	25.1	1.06 (0.58-1.95)	2.4
	19.3	24.3	0.74 (0.41-1.33)	2.4
	32.6	35.8	0.80 (0.39-1.64)	1.7
	12.4	19.0	0.66 (0.29-1.51)	1.3
	23.7	20.4	1.19 (0.56-2.51)	1.5
Kenya	3.5	3.4	1.11 (0.36-3.36)	0.7
	3.8	1.5	1.80 (0.37-8.73)	0.4
Nigeria	17.0	26.2	0.56 (0.25-1.25)	1.3
	12.1	12.3	0.91 (0.38-2.18)	1.1
	8.4	8.3	0.80 (0.14-4.63)	0.3
	13.9	16.9	0.80 (0.36-1.77)	1.4
	5.8	5.6	0.95 (0.36-2.53)	0.9
	27.8	16.2	1.91 (0.97-3.76)	1.9
	9.2	21.1	0.38 (0.17-0.87)	1.3
	22.8	23.7	0.97 (0.51-1.84)	2.1
	9.4	5.4	1.53 (0.78-3.00)	1.9
**Low income**				
Uganda	8.2	11.5	0.69 (0.34-1.39)	1.8
**Overall**			**1.01 (0.92-1.11)**	**100**

Supplementary Figure 17: Individual and pooled non-population-based estimates of the association between lockdown and the odds of preterm birth among all births 22 weeks onwards, in the first month of lockdown. Individual dataset odds ratios (represented by boxes on plot) were calculated by comparing the observed odds of preterm birth in the first month of lockdown to the forecasted odds of preterm birth in the first month of lockdown from a linear regression model model that was fitted to pre-lockdown data. Horizontal lines surrounding each box on the forest plot are 95% confidence intervals. Arrows indicate upper and or lower bounds of the confidence interval that are outside the x-axis limits.

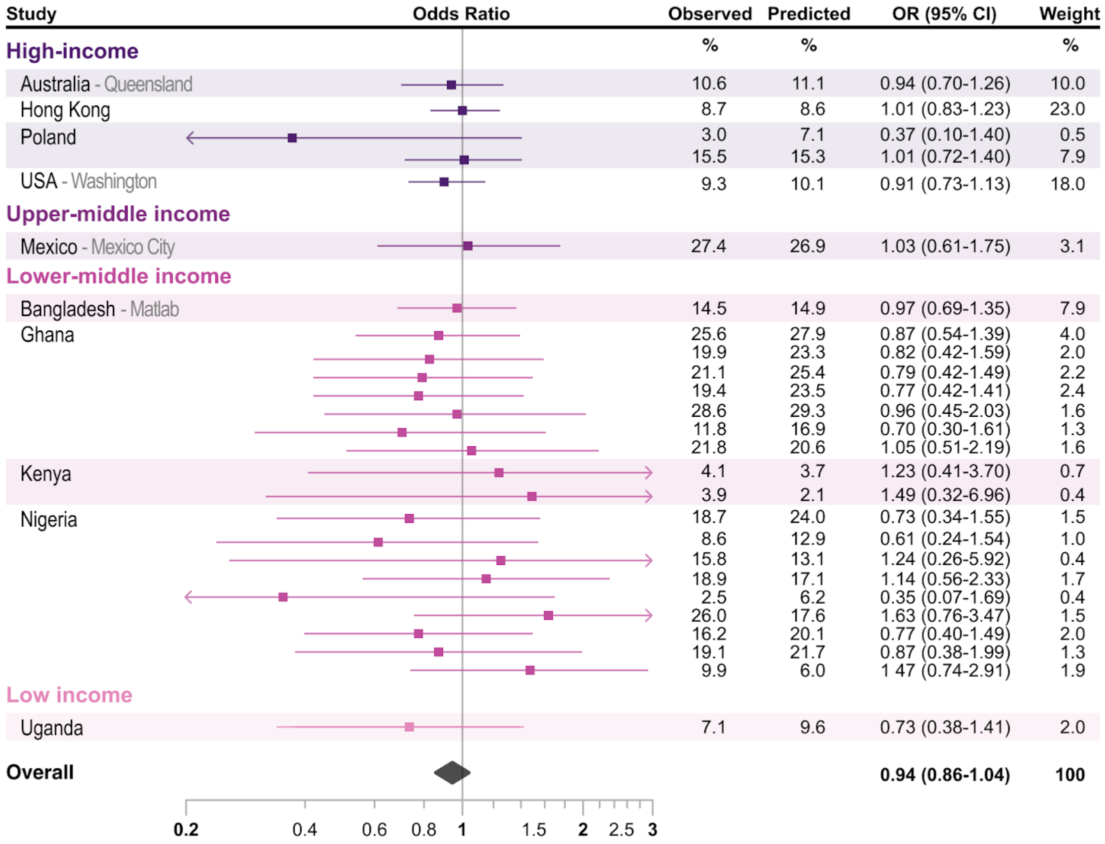

Supplementary Figure 18: Individual and pooled ***non-population-based estimates*** *of the association between lockdown and the odds of* ***preterm birth*** *among all births 22 weeks onwards, in the* ***second month*** *of lockdown. Individual dataset odds ratios (represented by boxes on plot) were calculated by comparing the observed odds of preterm birth in the second month of lockdown to the forecasted odds of preterm birth in the second month of lockdown from a linear regression model model that was fitted to pre-lockdown data. Horizontal lines surrounding each box on the forest plot are 95% confidence intervals. Arrows indicate upper and or lower bounds of the confidence interval that are outside the x-axis limits.*

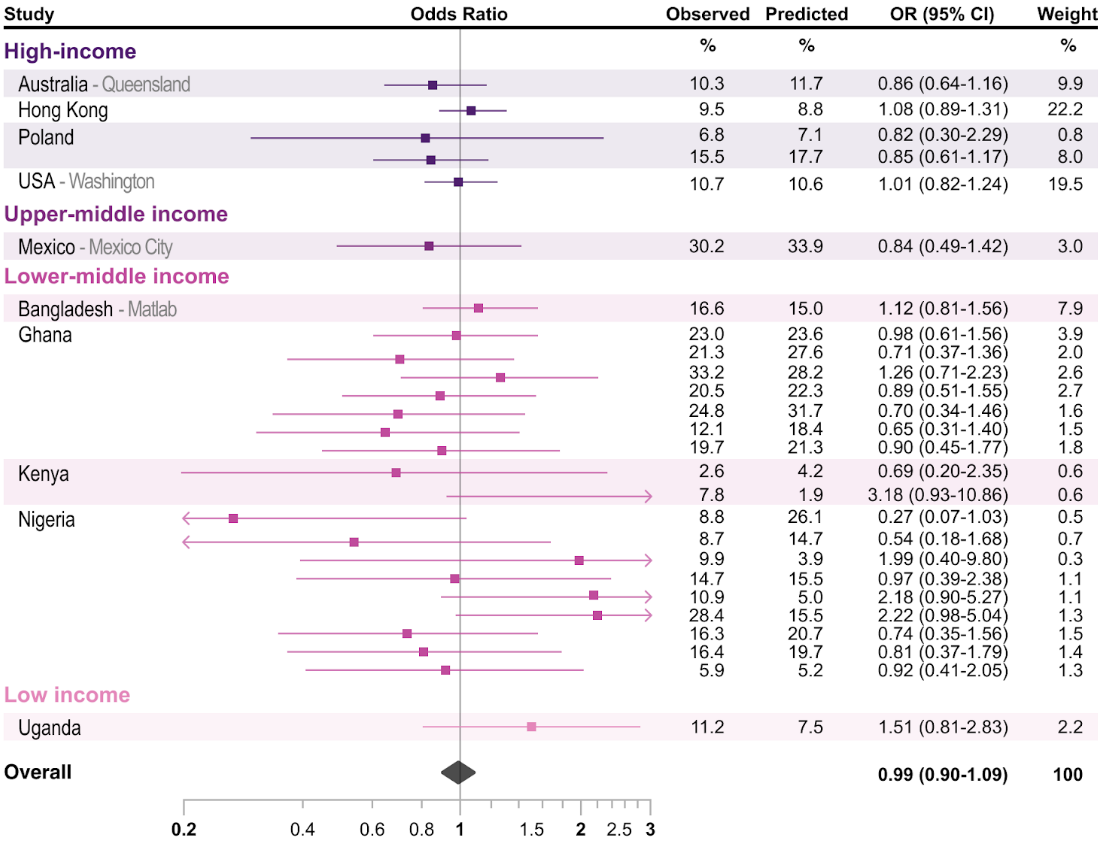

*Supplementary Figure 19: Individual and pooled **non-population-based estimates** of the association between lockdown and the odds of **preterm birth** among all births 22 weeks onwards, in the **third month** of lockdown. Individual dataset odds ratios (represented by boxes on plot) were calculated by comparing the observed odds of preterm birth in the third month of lockdown to the forecasted odds of preterm birth in the third month of lockdown from a linear regression model model that was fitted to pre-lockdown data. Horizontal lines surrounding each box on the forest plot are 95% confidence intervals. Arrows indicate upper and or lower bounds of the confidence interval that are outside the x-axis limits.*

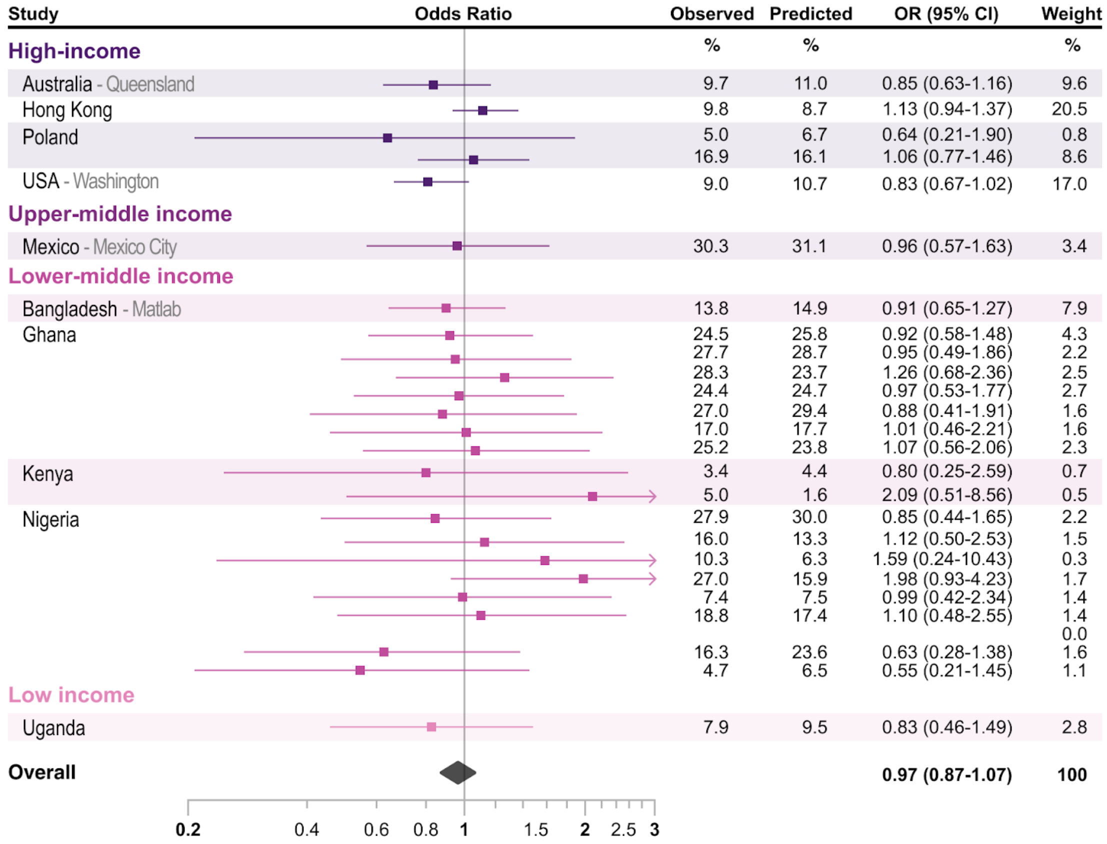

Supplementary Figure 20: Individual and pooled ***non-population-based estimates*** *of the association between lockdown and the odds of* ***<u>preterm birth</u>*** *among all births 22 weeks onwards, in the* ***fourth month*** *of lockdown. Individual dataset odds ratios (represented by boxes on plot) were calculated by comparing the observed odds of preterm birth in the fourth month of lockdown to the forecasted odds of preterm birth in the fourth month of lockdown from a linear regression model model that was fitted to pre-lockdown data. Horizontal lines surrounding each box on the forest plot are 95% confidence intervals. Arrows indicate upper and or lower bounds of the confidence interval that are outside the x-axis limits. .*

Association between lockdown and <u>very preterm birth</u> rates, by time since

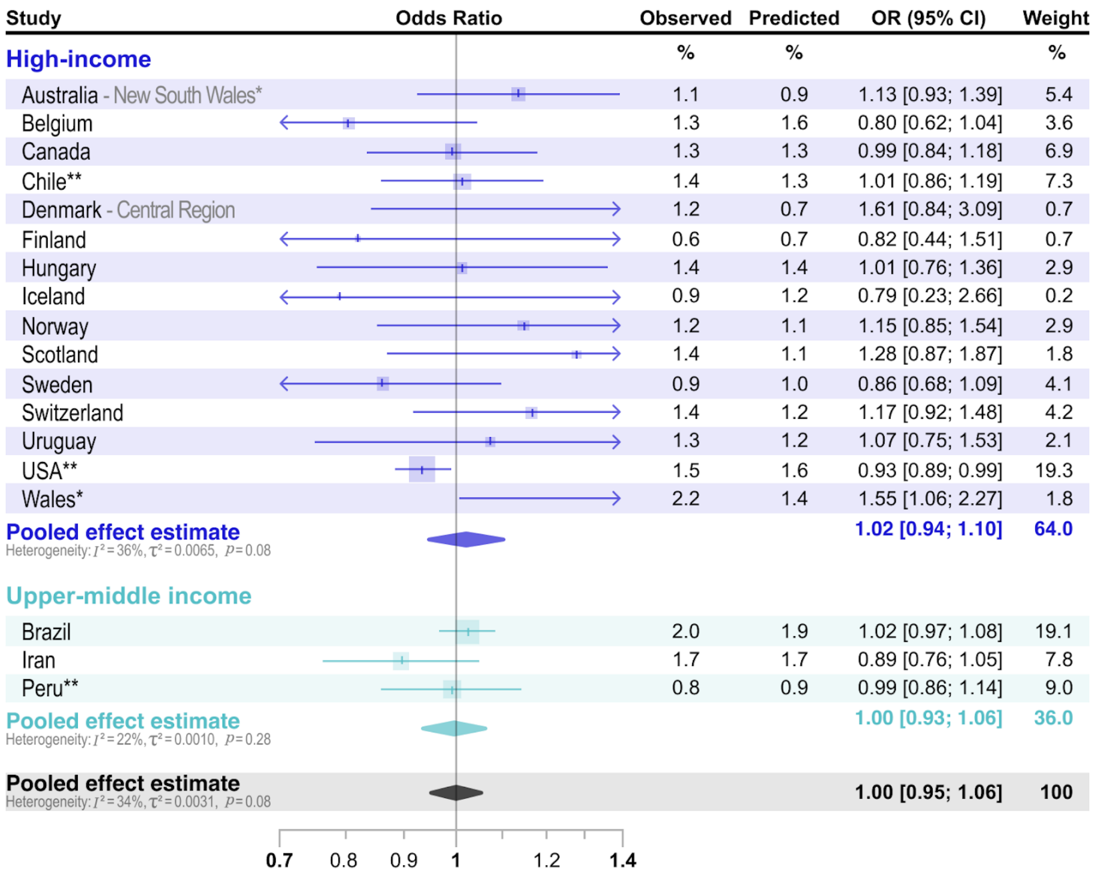

*Supplementary Figure 21: Individual and pooled **population-based estimates** of the association between lockdown and the odds of <u>**very preterm birth**</u> among all births 22 weeks onwards, in the **first month** of lockdown. Individual country odds ratios (represented by boxes on plot) were calculated by comparing the observed odds of very preterm birth in the first month of lockdown to the forecasted odds of very preterm birth in the first month of lockdown from an interrupted time series model that was fitted to pre-lockdown data. Horizontal lines surrounding each box on the forest plot are 95% confidence intervals. Arrows indicate upper and or lower bounds of the confidence interval that are outside the x-axis limits. *Births from 24 weeks onwards; **Live births only*

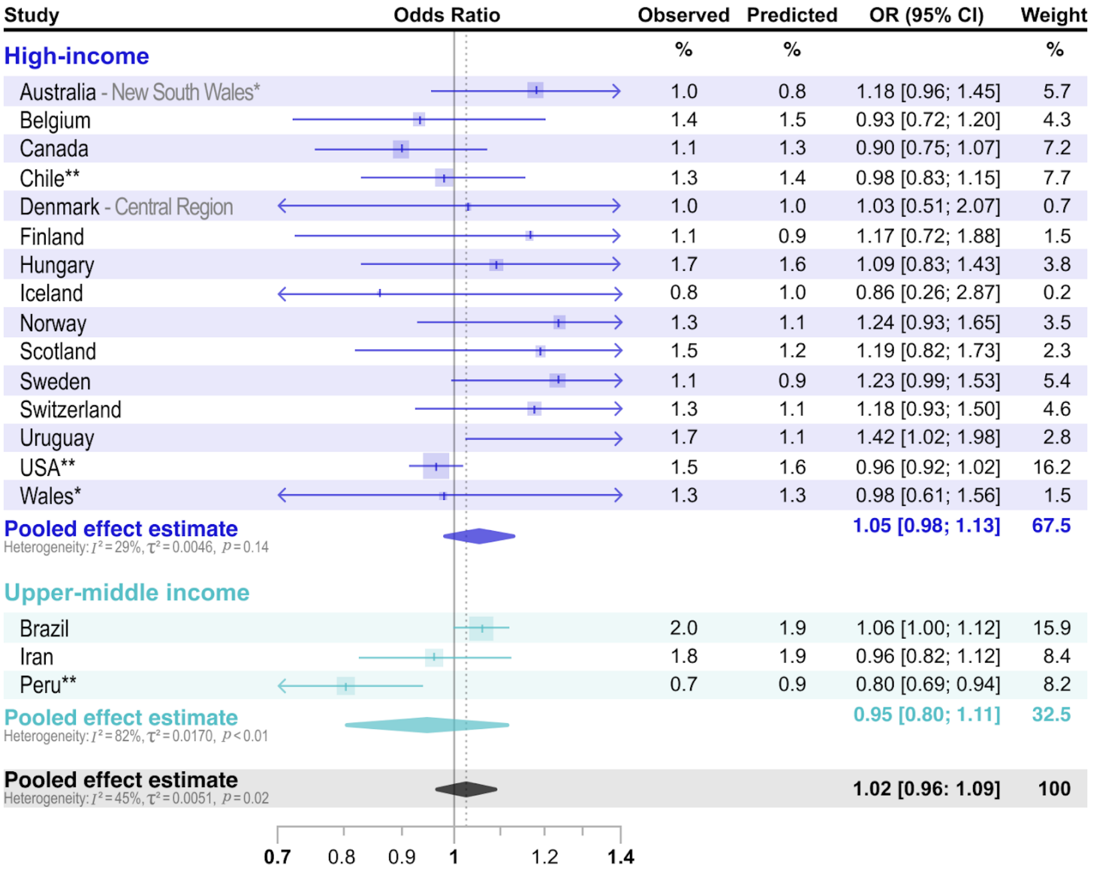

*Supplementary Figure 22: Individual and pooled **population-based estimates** of the association between lockdown and the odds of **very preterm birth** among all births 22 weeks onwards, in the **second month** of lockdown. Individual country odds ratios (represented by boxes on plot) were calculated by comparing the observed odds of very preterm birth in the second month of lockdown to the forecasted odds of very preterm birth in the second month of lockdown from an interrupted time series model that was fitted to pre-lockdown data. Horizontal lines surrounding each box on the forest plot are 95% confidence intervals. Arrows indicate upper and or lower bounds of the confidence interval that are outside the x-axis limits. *Births from 24 weeks onwards; **Live births only*

Study	Observed %	Predicted %	OR (95% CI)	Weight %
**High-income**				
Australia - New South Wales*	0.9	0.9	0.98 [0.78; 1.22]	5.5
Belgium	1.6	1.5	1.04 [0.82; 1.33]	4.8
Canada	1.1	1.3	0.85 [0.71; 1.02]	7.2
Chile**	1.3	1.3	1.01 [0.85; 1.19]	7.9
Denmark - Central Region	2.0	1.0	2.09 [1.21; 3.61]	1.2
Finland	0.7	0.7	0.99 [0.55; 1.77]	1.1
Hungary	1.5	1.7	0.87 [0.65; 1.15]	3.8
Iceland	0.6	1.3	0.42 [0.11; 1.67]	0.2
Norway	0.9	1.0	0.92 [0.67; 1.26]	3.1
Scotland	1.7	0.9	1.81 [1.25; 2.61]	2.5
Sweden	0.8	09	0.88 [0.68; 1.13]	4.6
Switzerland	1.2	1.0	1.19 [0.92; 1.52]	4.6
Uruguay	1.4	1.1	1.28 [0.90; 1.82]	2.7
USA**	1.5	1.5	0.98 [0.93; 1.03]	16.3
Wales*	1.3	1.5	0.87 [0.57; 1.33]	1.9
**Pooled effect estimate** Heterogeneity: $I^2 = 51\%, \tau^2 = 0.0127, p = 0.01$			**1.02 [0.93; 1.12]**	**67.2**
**Upper-middle income**				
Brazil	1.9	1.9	1.00 [0.94; 1.06]	15.8
Iran	1.9	1.8	1.05 [0.90; 1.23]	8.4
Peru**	0.8	0.8	0.90 [0.77; 1.05]	8.6
**Pooled effect estimate** Heterogeneity: $I^2 = 13\%, \tau^2 = 0.0006, p = 0.32$			**0.99 [0.93; 1.05]**	**32.8**
**Pooled effect estimate** Heterogeneity: $I^2 = 45\%, \tau^2 = 0.0054, p = 0.02$			**1.00 [0.94; 1.06]**	**100**

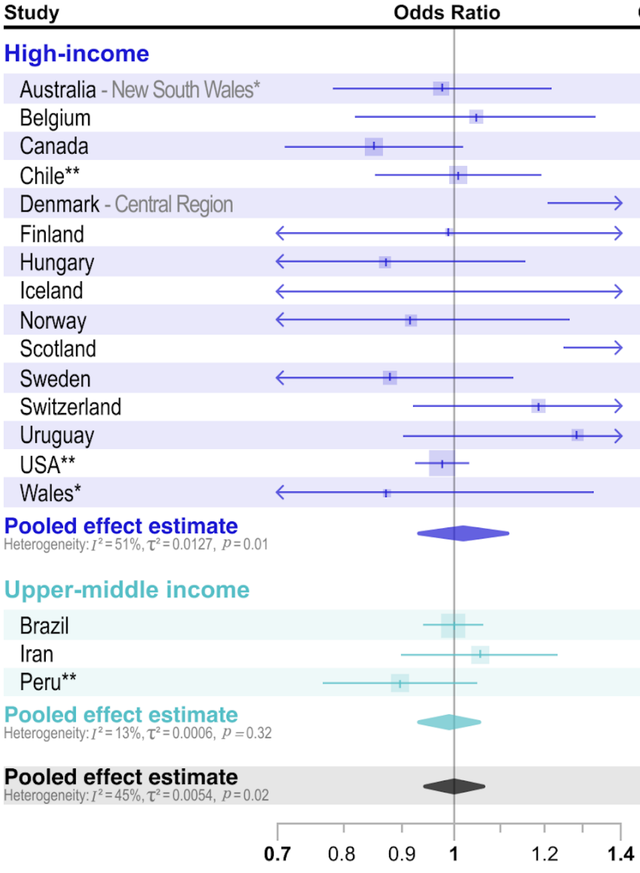

*Supplementary Figure 23: Individual and pooled **population-based estimates** of the association between lockdown and the odds of **very preterm birth** among all births 22 weeks onwards, in the **third month** of lockdown. Individual country odds ratios (represented by boxes on plot) were calculated by comparing the observed odds of very preterm birth in the third month of lockdown to the forecasted odds of very preterm birth in the third month of lockdown from an interrupted time series model that was fitted to pre-lockdown data. Horizontal lines surrounding each box on the forest plot are 95% confidence intervals. Arrows indicate upper and or lower bounds of the confidence interval that are outside the x-axis limits. *Births from 24 weeks onwards; **Live births only*

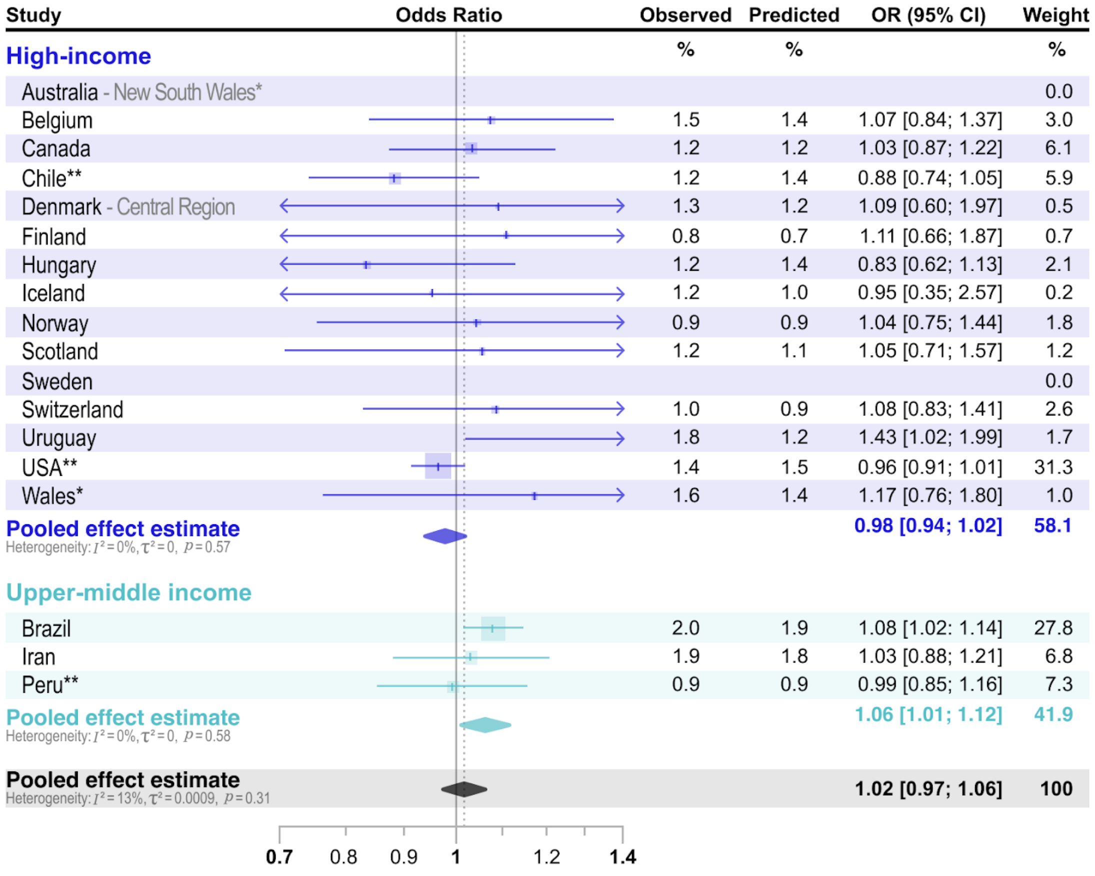

*Supplementary Figure 24: Individual and pooled **population-based estimates** of the association between lockdown and the odds of **very preterm birth** among all births 22 weeks onwards, in the **fourth month** of lockdown. Individual country odds ratios (represented by boxes on plot) were calculated by comparing the observed odds of very preterm birth in the fourth month of lockdown to the forecasted odds of very preterm birth in the fourth month of lockdown from an interrupted time series model that was fitted to pre-lockdown data. Horizontal lines surrounding each box on the forest plot are 95% confidence intervals. Arrows indicate upper and or lower bounds of the confidence interval that are outside the x-axis limits. *Births from 24 weeks onwards; **Live births only*

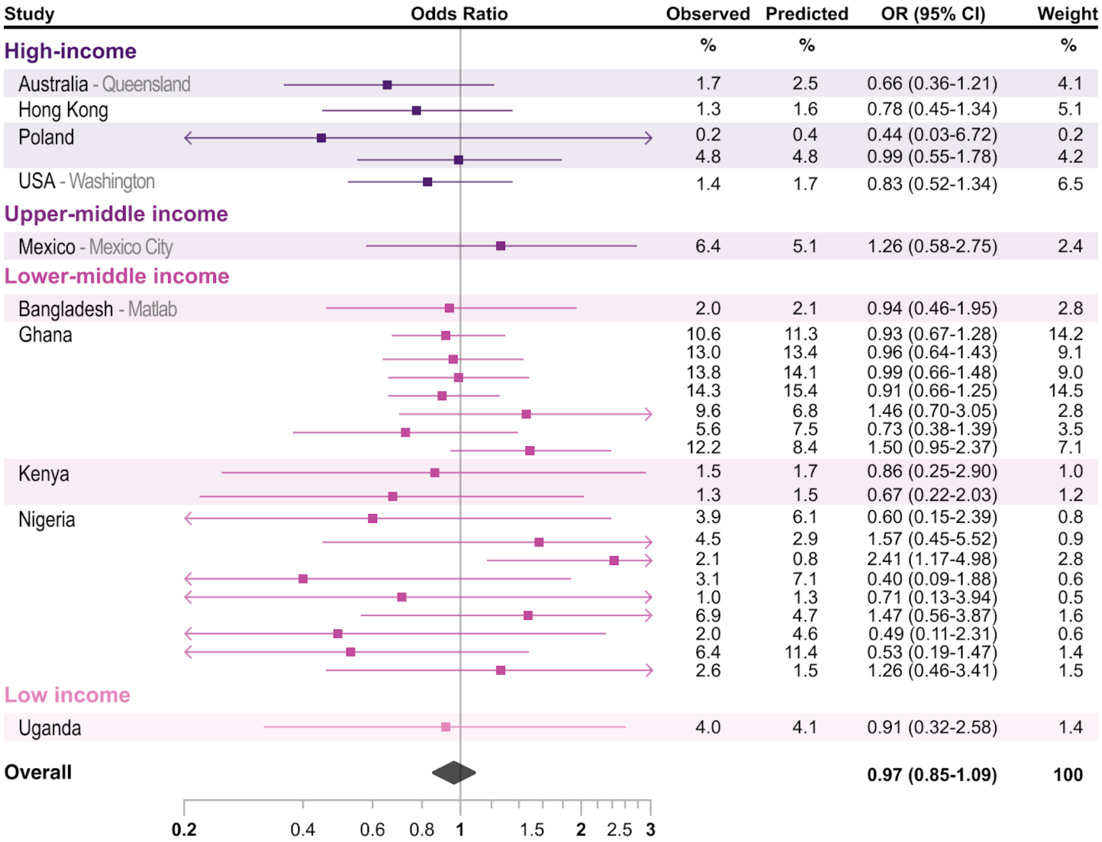

*Supplementary Figure 25: Individual and pooled **non-population-based estimates** of the association between lockdown and the odds of **very preterm birth** among all births 22 weeks onwards, in the **first month** of lockdown. Individual dataset odds ratios (represented by boxes on plot) were calculated by comparing the observed odds of very preterm birth in the first month of lockdown to the forecasted odds of very preterm birth in the first month of lockdown from a linear regression model model that was fitted to pre-lockdown data. Horizontal lines surrounding each box on the forest plot are 95% confidence intervals. Arrows indicate upper and or lower bounds of the confidence interval that are outside the x-axis limits.*

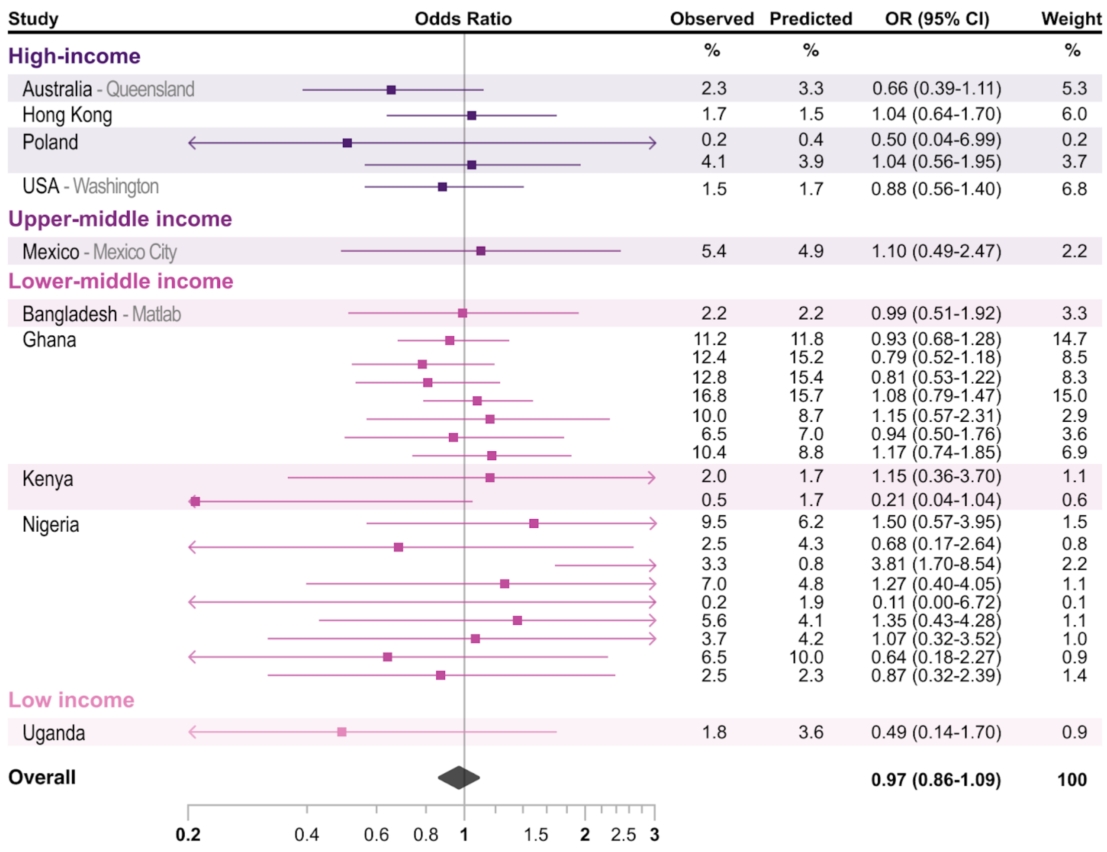

Supplementary Figure 26: Individual and pooled ***non-population-based estimates*** *of the association between lockdown and the odds of* ***very preterm birth*** *among all births 22 weeks onwards, in the* ***second month*** *of lockdown. Individual dataset odds ratios (represented by boxes on plot) were calculated by comparing the observed odds of very preterm birth in the second month of lockdown to the forecasted odds of very preterm birth in the second month of lockdown from a linear regression model model that was fitted to pre-lockdown data. Horizontal lines surrounding each box on the forest plot are 95% confidence intervals. Arrows indicate upper and or lower bounds of the confidence interval that are outside the x-axis limits.*

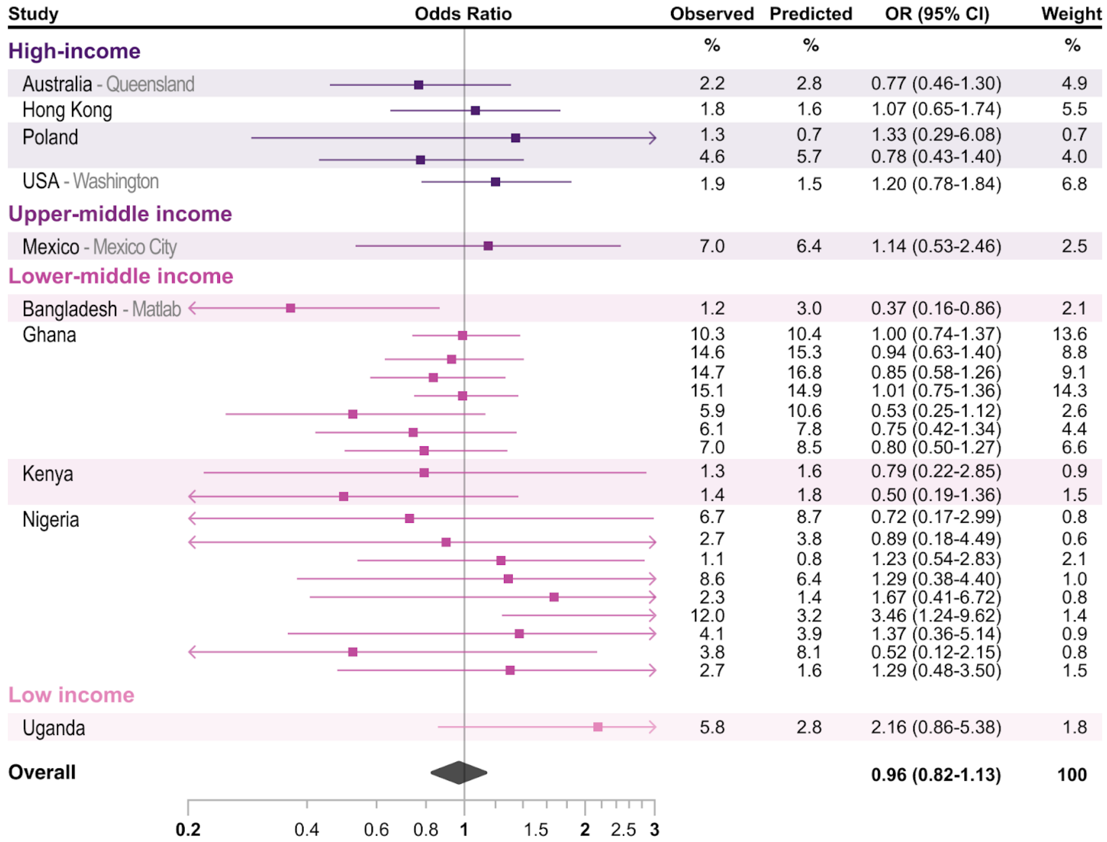

Supplementary Figure 27: Individual and pooled ***non-population-based estimates*** *of the association between lockdown and the odds of* ***very preterm birth*** *among all births 22 weeks onwards, in the* ***third month*** *of lockdown. Individual dataset odds ratios (represented by boxes on plot) were calculated by comparing the observed odds of very preterm birth in the third month of lockdown to the forecasted odds of very preterm birth in the third month of lockdown from a linear regression model model that was fitted to pre-lockdown data. Horizontal lines surrounding each box on the forest plot are 95% confidence intervals. Arrows indicate upper and or lower bounds of the confidence interval that are outside the x-axis limits.*

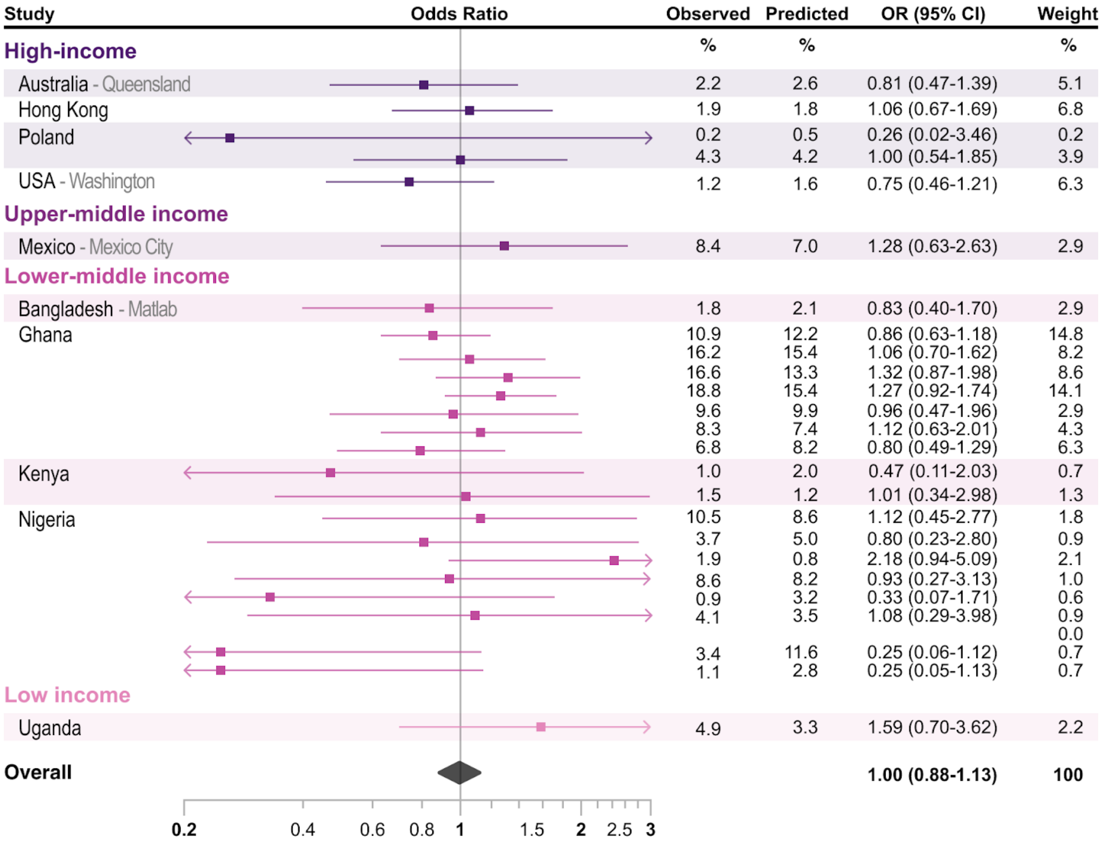

*Supplementary Figure 28: Individual and pooled **non-population-based estimates** of the association between lockdown and the odds of **very preterm birth** among all births 22 weeks onwards, in the **fourth month** of lockdown. Individual dataset odds ratios (represented by boxes on plot) were calculated by comparing the observed odds of very preterm birth in the fourth month of lockdown to the forecasted odds of very preterm birth in the fourth month of lockdown from a linear regression model model that was fitted to pre-lockdown data. Horizontal lines surrounding each box on the forest plot are 95% confidence intervals. Arrows indicate upper and or lower bounds of the confidence interval that are outside the x-axis limits.*

Association between lockdown and <u>spontaneous preterm birth</u> rates, by time since lockdown

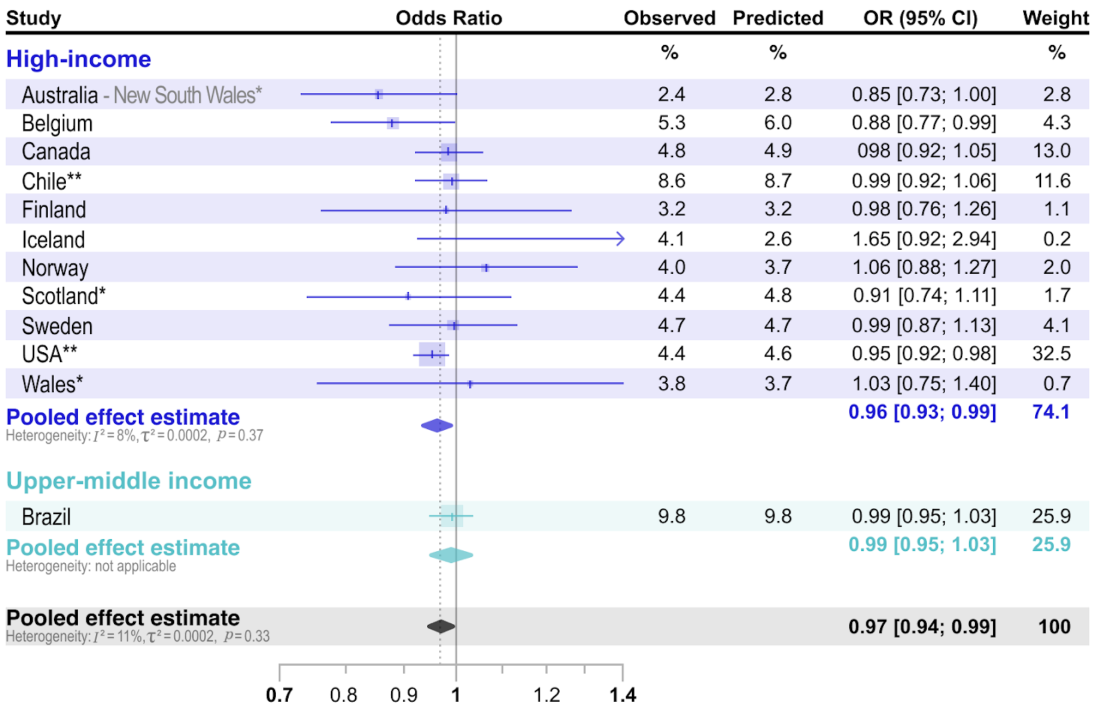

*Supplementary Figure 29: Individual and pooled **population-based estimates** of the association between lockdown and the odds of **<u>spontaneous preterm birth</u>** among all births 22 weeks onwards, in the **first month** of lockdown. Individual country odds ratios (represented by boxes on plot) were calculated by comparing the observed odds of spontaneous preterm birth in the first month of lockdown to the forecasted odds of spontaneous preterm birth in the first month of lockdown from an interrupted time series model that was fitted to pre-lockdown data. Horizontal lines surrounding each box on the forest plot are 95% confidence intervals. Arrows indicate upper and or lower bounds of the confidence interval that are outside the x-axis limits. *Restricted to births from 24 weeks onwards in New South Wales, Australia and Wales, and from 28 weeks onwards in Scotland; **Live births only*

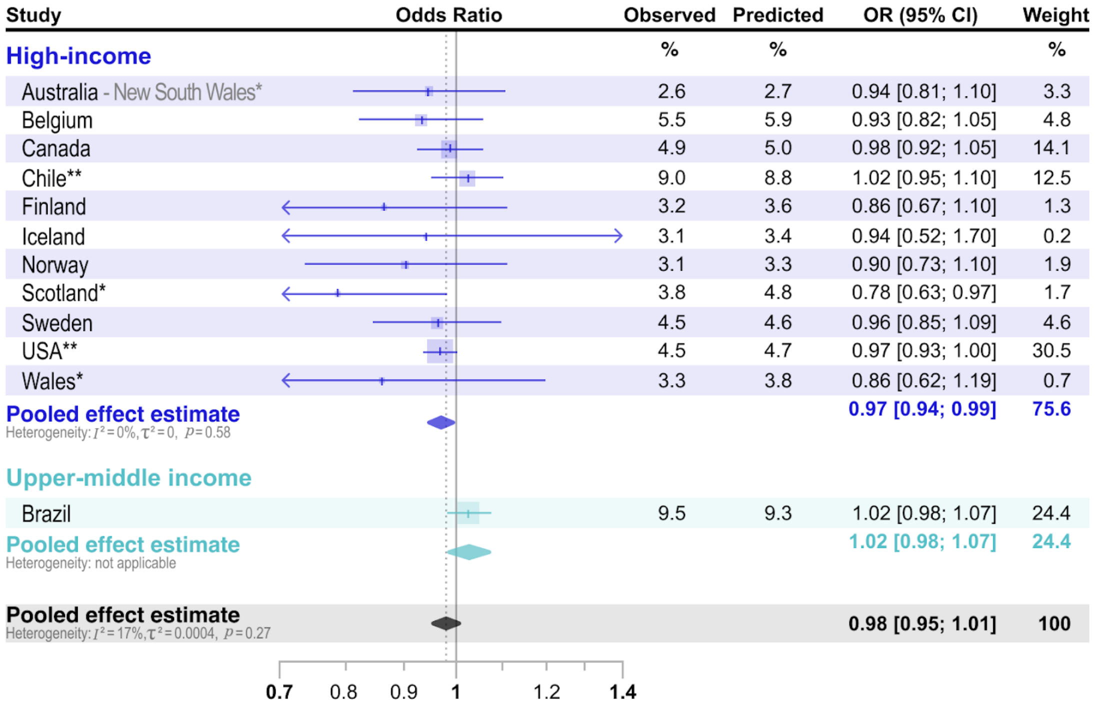

*Supplementary Figure 30: Individual and pooled **population-based estimates** of the association between lockdown and the odds of **spontaneous preterm birth** among all births 22 weeks onwards, in the **second month** of lockdown. Individual country odds ratios (represented by boxes on plot) were calculated by comparing the observed odds of spontaneous preterm birth in the second month of lockdown to the forecasted odds of spontaneous preterm birth in the second month of lockdown from an interrupted time series model that was fitted to pre-lockdown data. Horizontal lines surrounding each box on the forest plot are 95% confidence intervals. Arrows indicate upper and or lower bounds of the confidence interval that are outside the x-axis limits. *Restricted to births from 24 weeks onwards in New South Wales, Australia and Wales, and from 28 weeks onwards in Scotland; **Live births only*

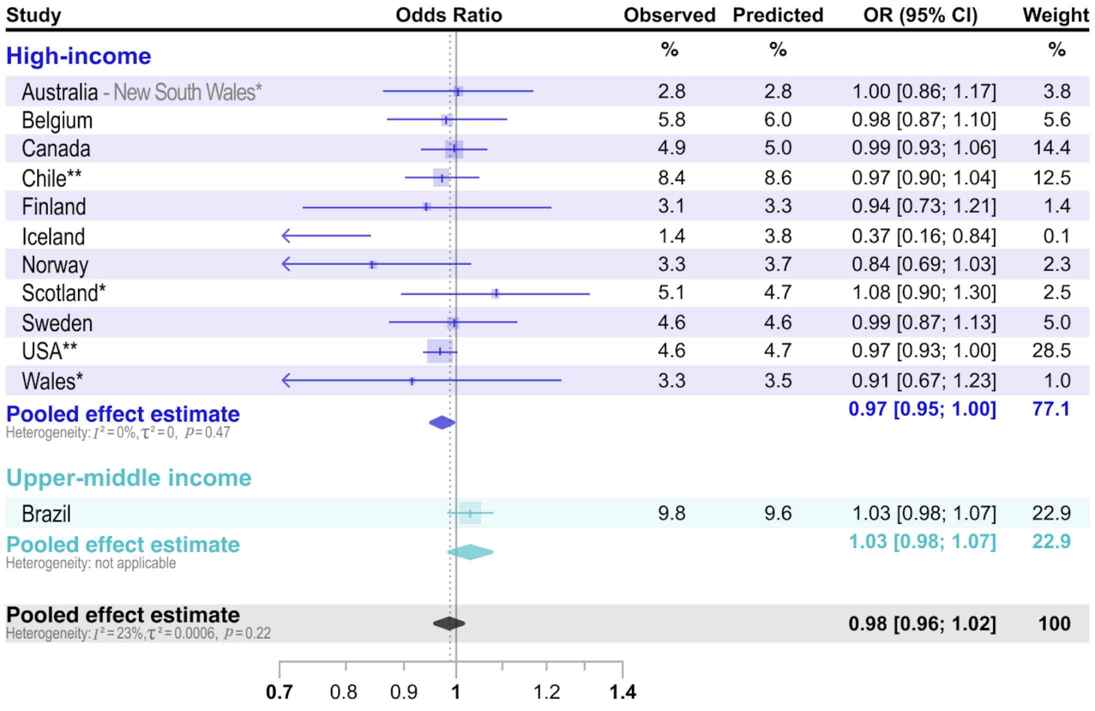

Supplementary Figure 31: Individual and pooled ***population-based estimates*** *of the association between lockdown and the odds of* ***spontaneous preterm birth*** *among all births 22 weeks onwards, in the* ***third month*** *of lockdown. Individual country odds ratios (represented by boxes on plot) were calculated by comparing the observed odds of spontaneous preterm birth in the third month of lockdown to the forecasted odds of spontaneous preterm birth in the third month of lockdown from an interrupted time series model that was fitted to pre-lockdown data. Horizontal lines surrounding each box on the forest plot are 95% confidence intervals. Arrows indicate upper and or lower bounds of the confidence interval that are outside the x-axis limits. *Restricted to births from 24 weeks onwards in New South Wales, Australia and Wales, and from 28 weeks onwards in Scotland; **Live births only*

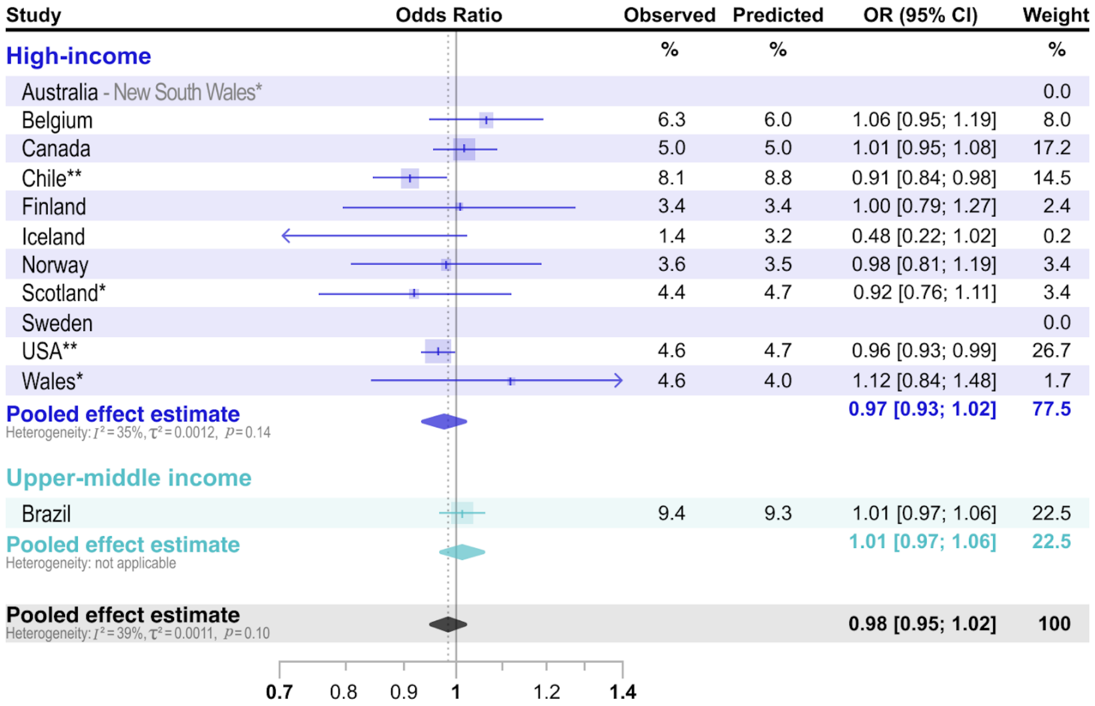

*Supplementary Figure 32: Individual and pooled **population-based estimates** of the association between lockdown and the odds of **spontaneous preterm birth** among all births 22 weeks onwards, in the **fourth month** of lockdown. Individual country odds ratios (represented by boxes on plot) were calculated by comparing the observed odds of spontaneous preterm birth in the fourth month of lockdown to the forecasted odds of spontaneous preterm birth in the fourth month of lockdown from an interrupted time series model that was fitted to pre-lockdown data. Horizontal lines surrounding each box on the forest plot are 95% confidence intervals. Arrows indicate upper and or lower bounds of the confidence interval that are outside the x-axis limits. *Restricted to births from 24 weeks onwards in New South Wales, Australia and Wales, and from 28 weeks onwards in Scotland; **Live births only*

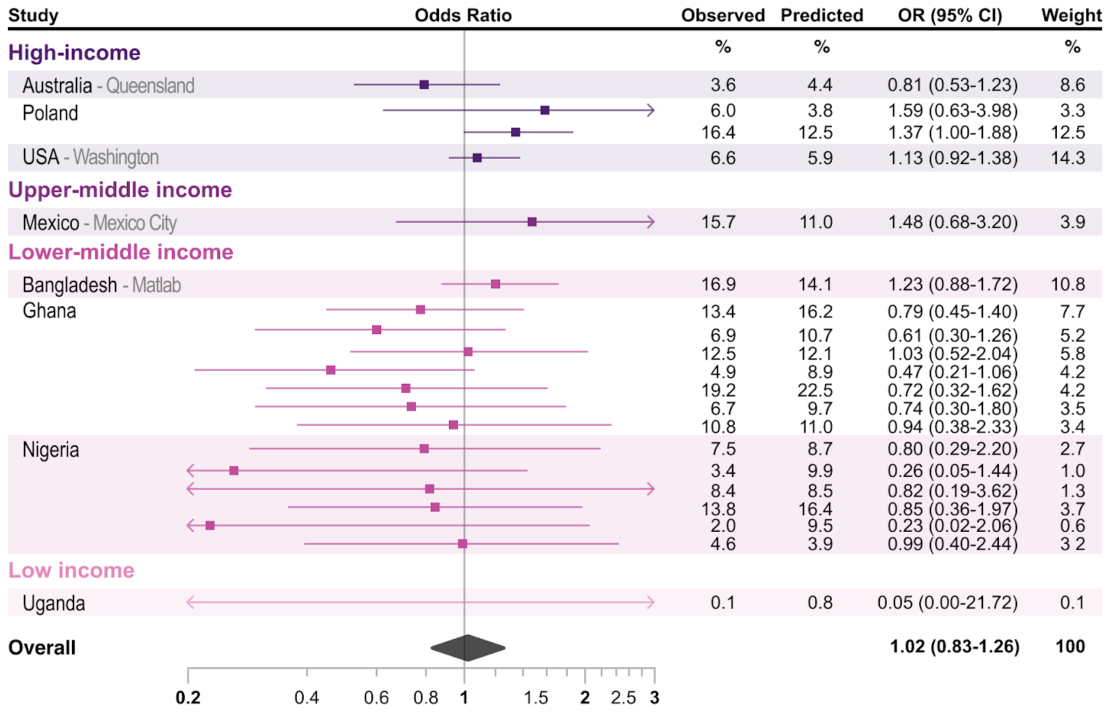

*Supplementary Figure 33: Individual and pooled **non-population-based estimates** of the association between lockdown and the odds of **spontaneous preterm birth** among all births 22 weeks onwards, in the **first month** of lockdown. Individual dataset odds ratios (represented by boxes on plot) were calculated by comparing the observed odds of spontaneous preterm birth in the first month of lockdown to the forecasted odds of spontaneous preterm birth in the first month of lockdown from a linear regression model model that was fitted to pre-lockdown data. Horizontal lines surrounding each box on the forest plot are 95% confidence intervals. Arrows indicate upper and or lower bounds of the confidence interval that are outside the x-axis limits.*

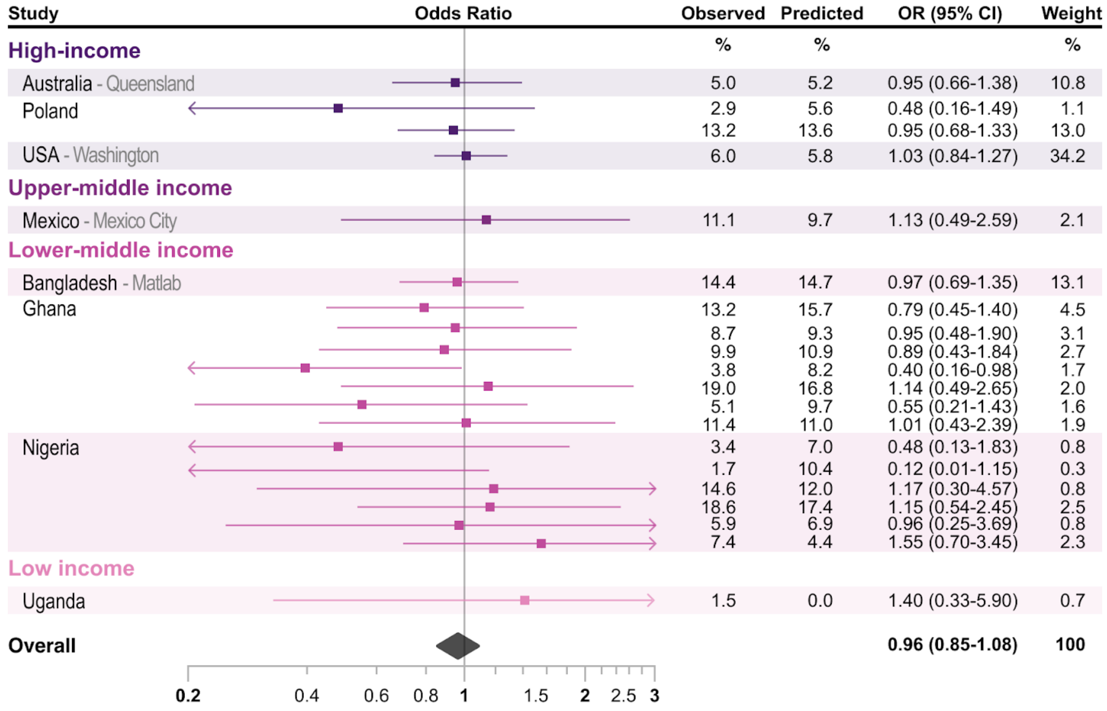

*Supplementary Figure 34: Individual and pooled **non-population-based estimates** of the association between lockdown and the odds of **spontaneous preterm birth** among all births 22 weeks onwards, in the **second month** of lockdown. Individual dataset odds ratios (represented by boxes on plot) were calculated by comparing the observed odds of spontaneous preterm birth in the second month of lockdown to the forecasted odds of spontaneous preterm birth in the second month of lockdown from a linear regression model model that was fitted to pre-lockdown data. Horizontal lines surrounding each box on the forest plot are 95% confidence intervals. Arrows indicate upper and or lower bounds of the confidence interval that are outside the x-axis limits*

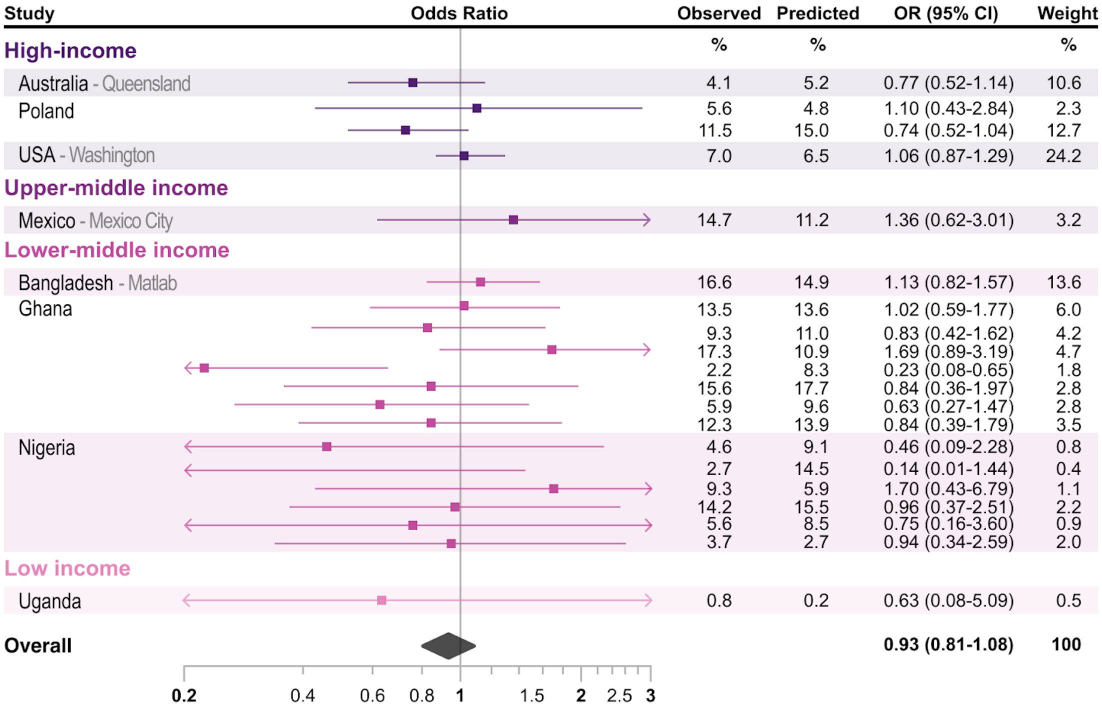

Supplementary Figure 35: Individual and pooled ***non-population-based estimates*** *of the association between lockdown and the odds of* ***spontaneous preterm birth*** *among all births 22 weeks onwards, in the* ***third month*** *of lockdown. Individual dataset odds ratios (represented by boxes on plot) were calculated by comparing the observed odds of spontaneous preterm birth in the third month of lockdown to the forecasted odds of spontaneous preterm birth in the third month of lockdown from a linear regression model model that was fitted to pre-lockdown data. Horizontal lines surrounding each box on the forest plot are 95% confidence intervals. Arrows indicate upper and or lower bounds of the confidence interval that are outside the x-axis limits*

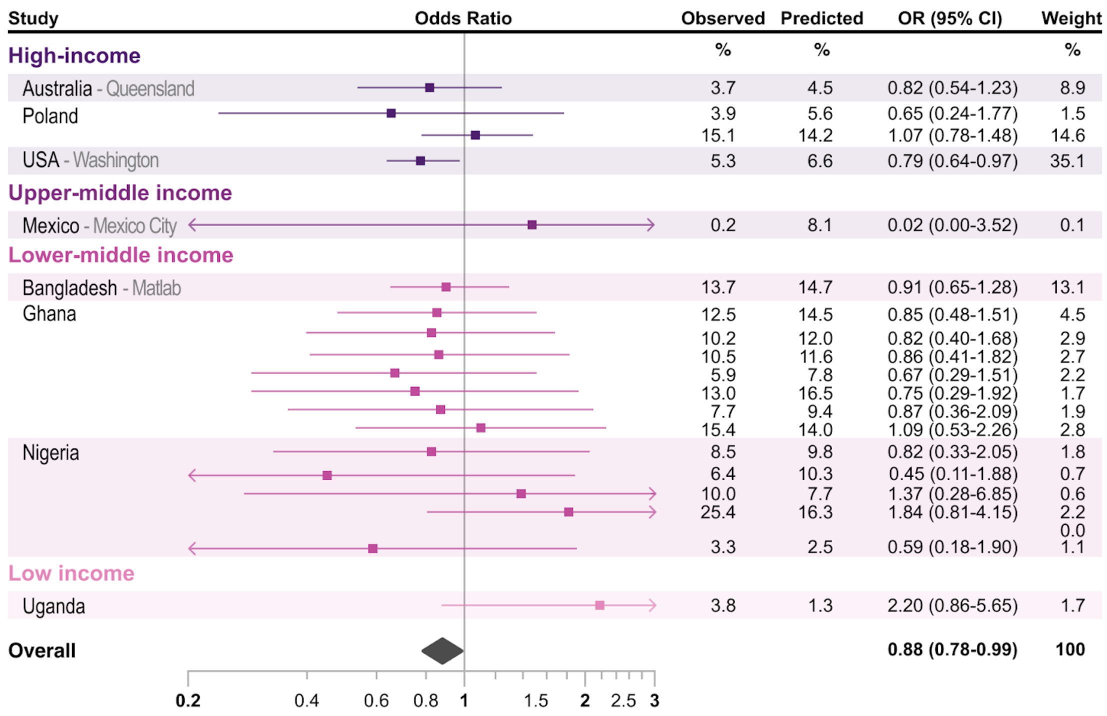

Supplementary Figure 36: Individual and pooled ***non-population-based estimates*** *of the association between lockdown and the odds of* ***spontaneous preterm birth*** *among all births 22 weeks onwards, in the* ***fourth month*** *of lockdown. Individual dataset odds ratios (represented by boxes on plot) were calculated by comparing the observed odds of spontaneous preterm birth in the fourth month of lockdown to the forecasted odds of spontaneous preterm birth in the fourth month of lockdown from a linear regression model model that was fitted to pre-lockdown data. Horizontal lines surrounding each box on the forest plot are 95% confidence intervals. Arrows indicate upper and or lower bounds of the confidence interval that are outside the x-axis limits*

Association between lockdown and stillbirth rates, by time since lockdown

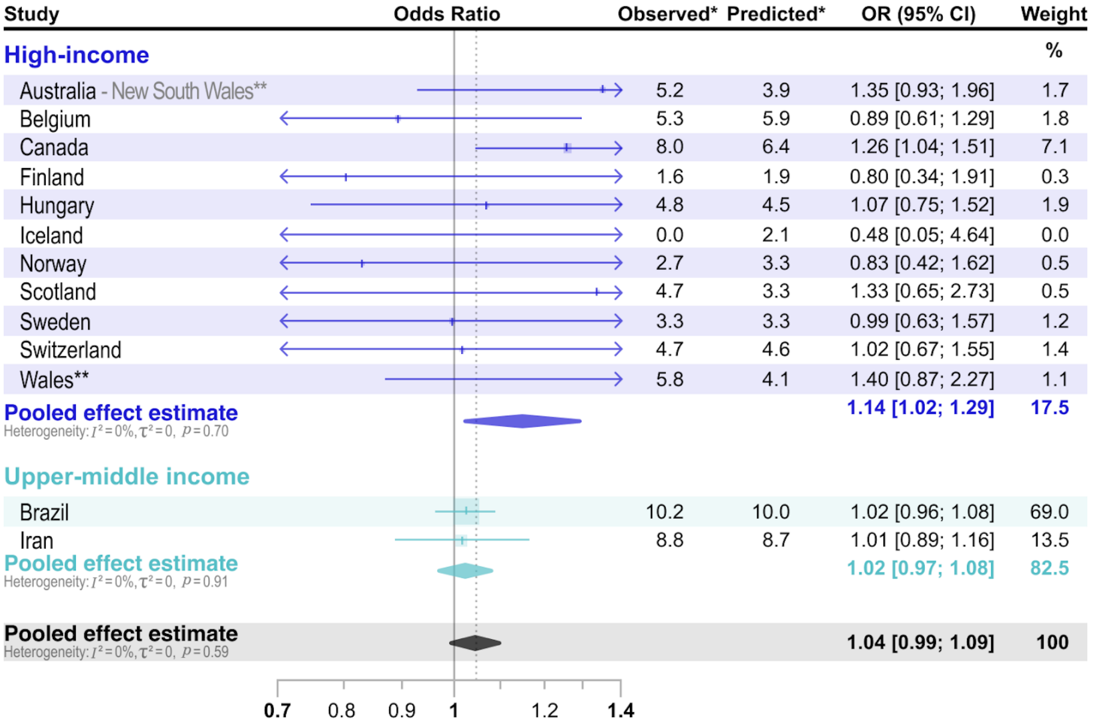

*Supplementary Figure 37: Individual and pooled **population-based estimates** of the association between lockdown and the odds of **stillbirth** among all births 22 weeks onwards, in the **first month** of lockdown. Individual country odds ratios (represented by boxes on plot) were calculated by comparing the observed odds of stillbirth in the first month of lockdown to the forecasted odds of stillbirth in the first month of lockdown from an interrupted time series model that was fitted to pre-lockdown data. Horizontal lines surrounding each box on the forest plot are 95% confidence intervals. Arrows indicate upper and or lower bounds of the confidence interval that are outside the x-axis limits. *Per 1000 births;**Restricted to births to births from 24 weeks onwards*

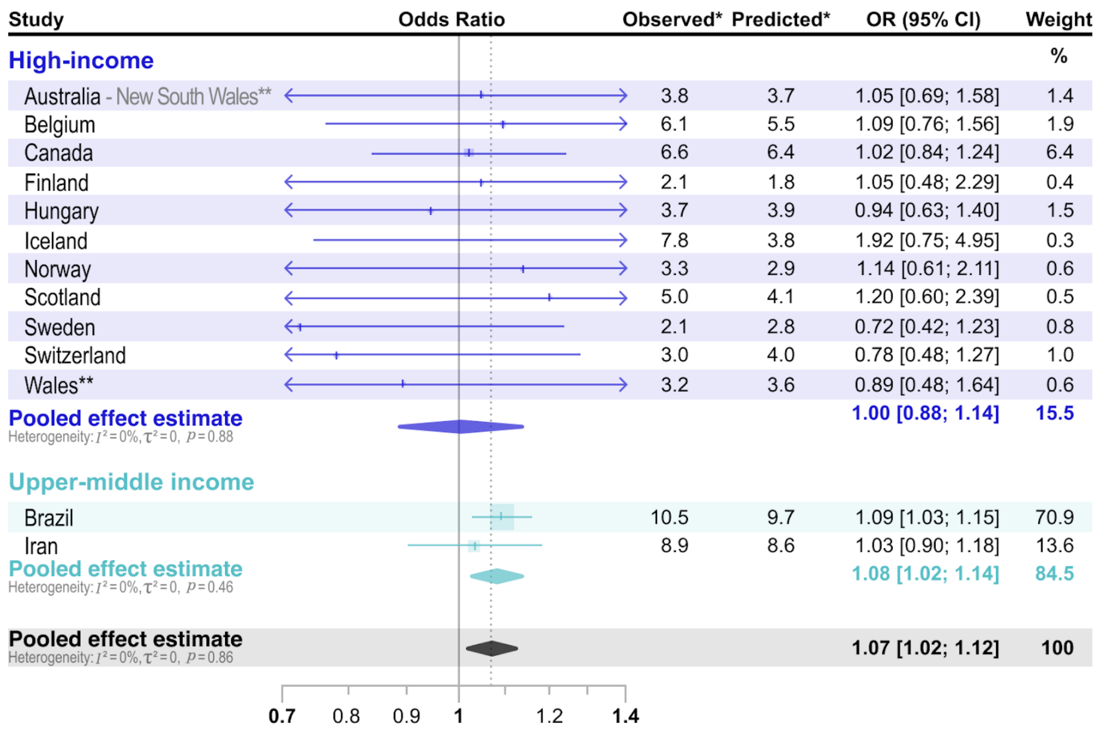

*Supplementary Figure 38: Individual and pooled **population-based estimates** of the association between lockdown and the odds of **stillbirth** among all births 22 weeks onwards, in the **second month** of lockdown. Individual country odds ratios (represented by boxes on plot) were calculated by comparing the observed odds of stillbirth in the second month of lockdown to the forecasted odds of stillbirth in the second month of lockdown from an interrupted time series model that was fitted to pre-lockdown data. Horizontal lines surrounding each box on the forest plot are 95% confidence intervals. Arrows indicate upper and or lower bounds of the confidence interval that are outside the x-axis limits. *Per 1000 births;**Restricted to births to births from 24 weeks onwards*

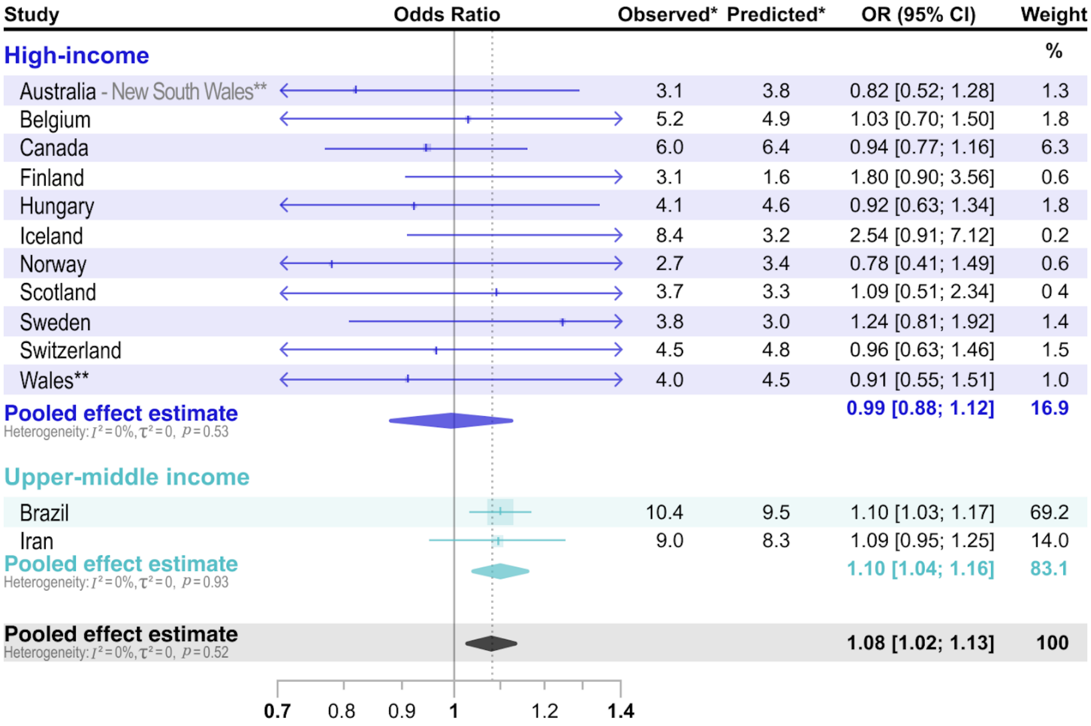

*Supplementary Figure 39: Individual and pooled **population-based estimates** of the association between lockdown and the odds of **stillbirth** among all births 22 weeks onwards, in the **third month** of lockdown. Individual country odds ratios (represented by boxes on plot) were calculated by comparing the observed odds of stillbirth in the third month of lockdown to the forecasted odds of stillbirth in the third month of lockdown from an interrupted time series model that was fitted to pre-lockdown data. Horizontal lines surrounding each box on the forest plot are 95% confidence intervals. Arrows indicate upper and or lower bounds of the confidence interval that are outside the x-axis limits. *Per 1000 births;**Restricted to births to births from 24 weeks onwards*

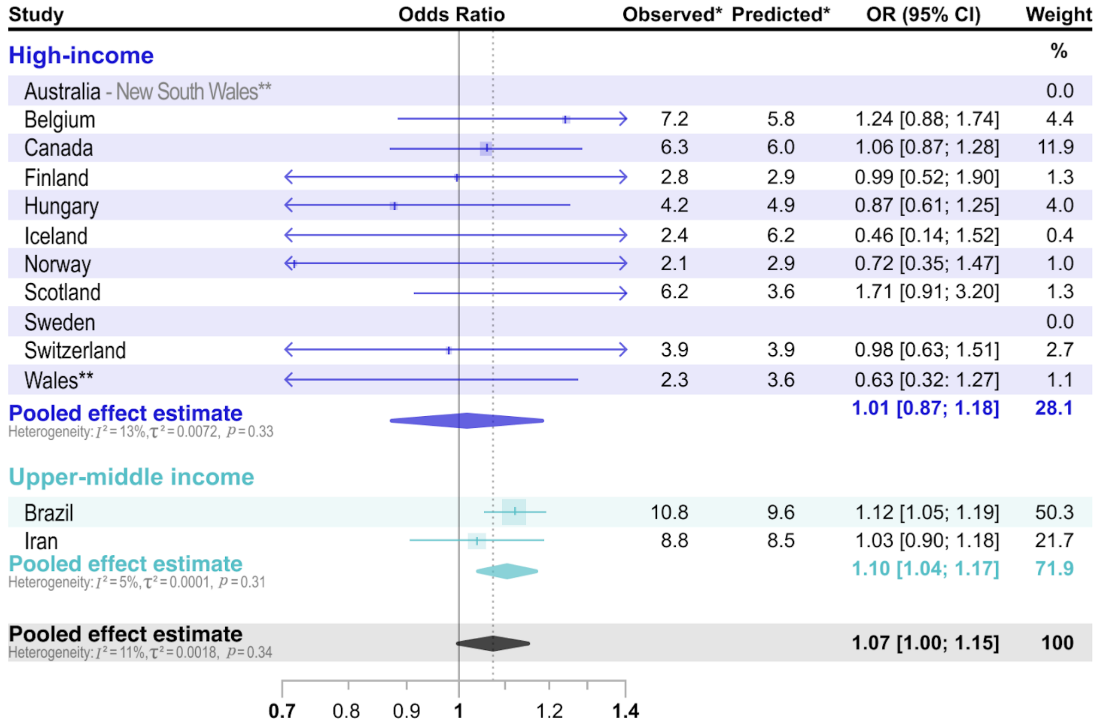

*Supplementary Figure 40: Individual and pooled **population-based estimates** of the association between lockdown and the odds of **stillbirth** among all births 22 weeks onwards, in the **fourth month** of lockdown. Individual country odds ratios (represented by boxes on plot) were calculated by comparing the observed odds of stillbirth in the fourth month of lockdown to the forecasted odds of stillbirth in the fourth month of lockdown from an interrupted time series model that was fitted to pre-lockdown data. Horizontal lines surrounding each box on the forest plot are 95% confidence intervals. Arrows indicate upper and or lower bounds of the confidence interval that are outside the x-axis limits. *Per 1000 births;**Restricted to births to births from 24 weeks onwards*

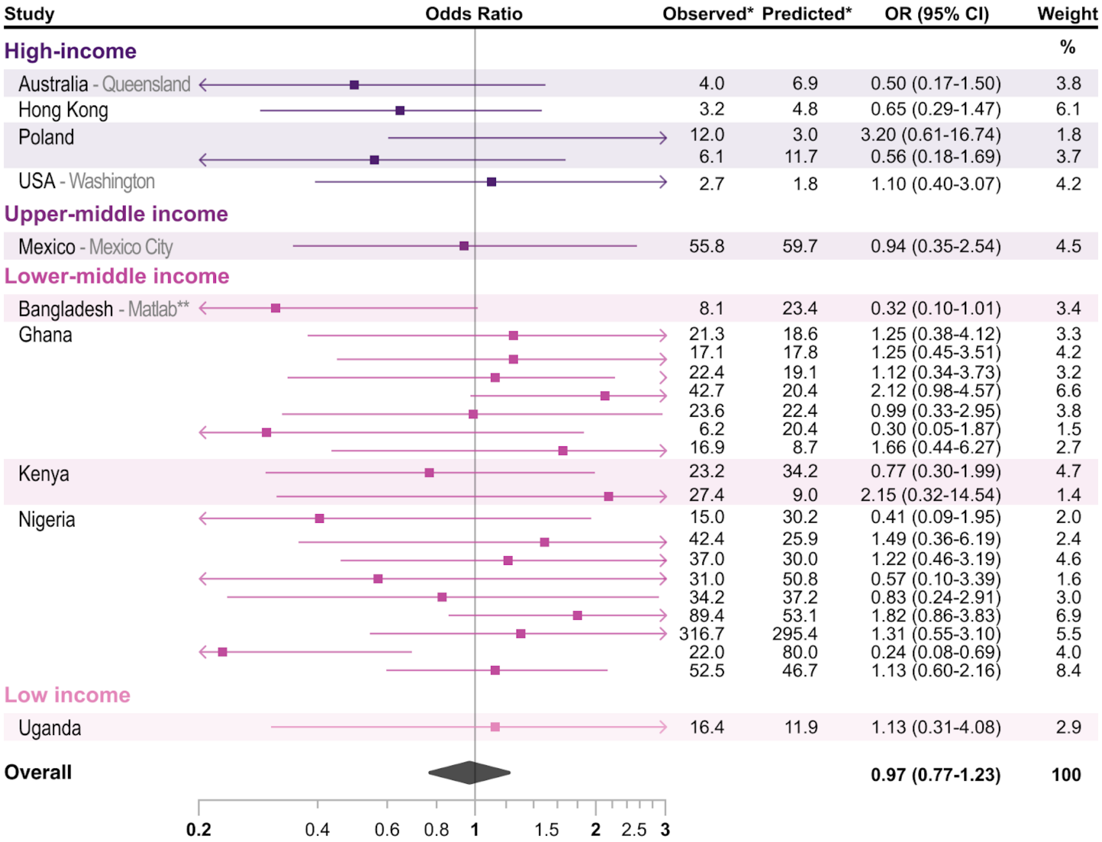

*Supplementary Figure 41: Individual and pooled **non-population-based estimates** of the association between lockdown and the odds of **stillbirth** among all births 22 weeks onwards, in the **first month** of lockdown. Individual dataset odds ratios (represented by boxes on plot) were calculated by comparing the observed odds of stillbirth in the first month of lockdown to the forecasted odds of stillbirth in the first month of lockdown from a linear regression model model that was fitted to pre-lockdown data. Horizontal lines surrounding each box on the forest plot are 95% confidence intervals. Arrows indicate upper and or lower bounds of the confidence interval that are outside the x-axis limits. *Per 1000 births;**Restricted to births from 28 weeks onwards*

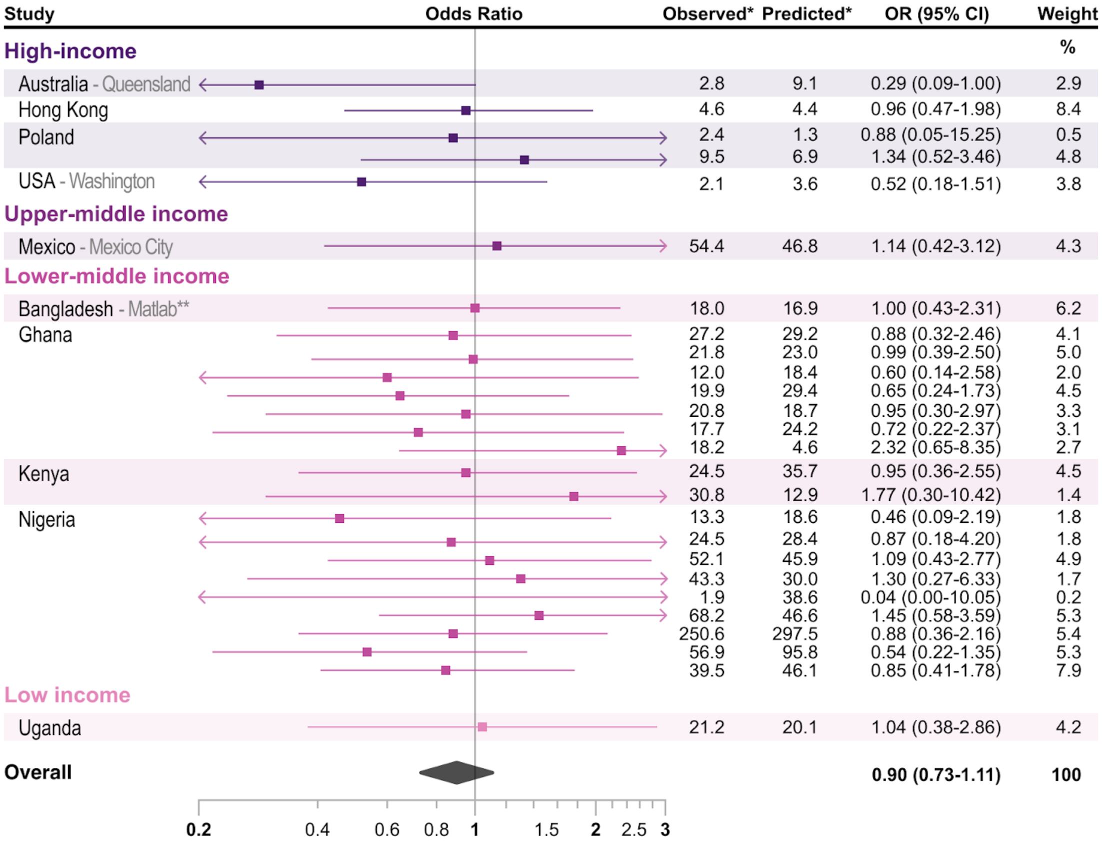

*Supplementary Figure 42: Individual and pooled **non-population-based estimates** of the association between lockdown and the odds of **stillbirth** among all births 22 weeks onwards, in the **second month** of lockdown. Individual dataset odds ratios (represented by boxes on plot) were calculated by comparing the observed odds of stillbirth in the second month of lockdown to the forecasted odds of stillbirth in the second month of lockdown from a linear regression model model that was fitted to pre-lockdown data. Horizontal lines surrounding each box on the forest plot are 95% confidence intervals. Arrows indicate upper and or lower bounds of the confidence interval that are outside the x-axis limits. *Per 1000 births;**Restricted to births from 28 weeks onwards*

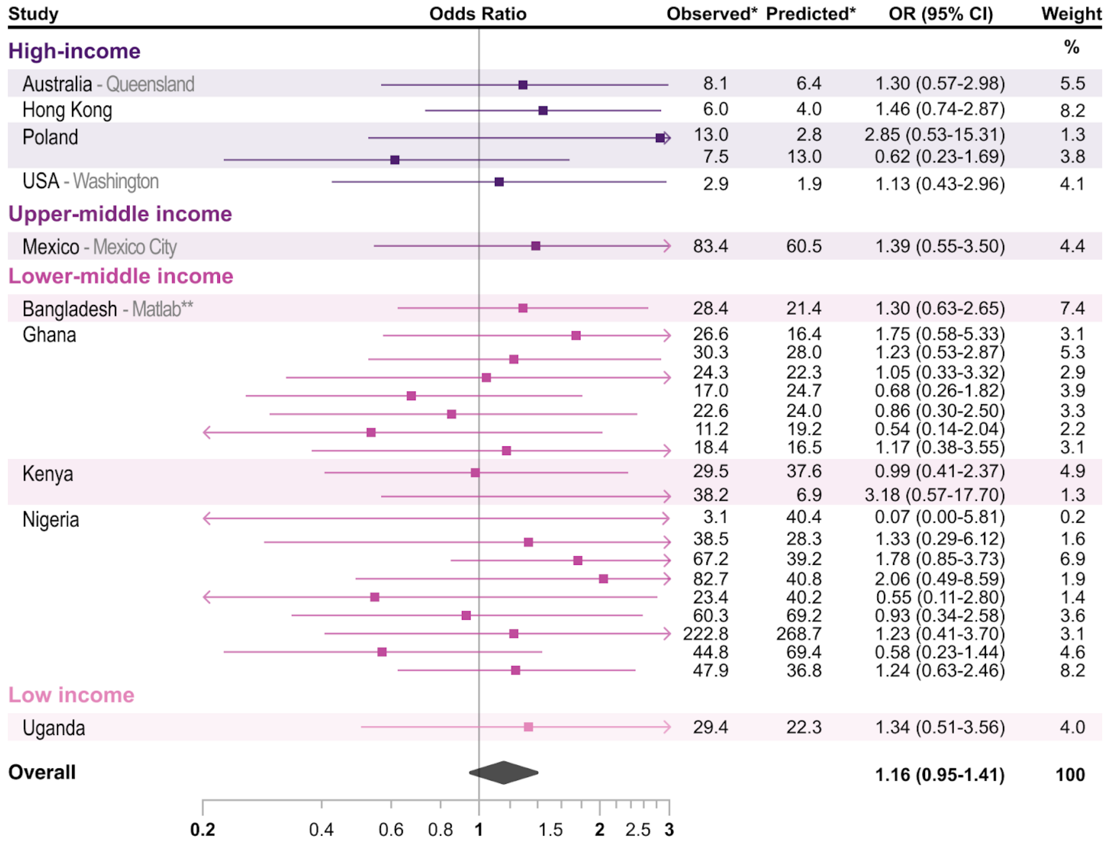

Supplementary Figure 43: Individual and pooled ***non-population-based estimates*** *of the association between lockdown and the odds of* ***stillbirth*** *among all births 22 weeks onwards, in the* ***third month*** *of lockdown. Individual dataset odds ratios (represented by boxes on plot) were calculated by comparing the observed odds of stillbirth in the third month of lockdown to the forecasted odds of stillbirth in the third month of lockdown from a linear regression model model that was fitted to pre-lockdown data. Horizontal lines surrounding each box on the forest plot are 95% confidence intervals. Arrows indicate upper and or lower bounds of the confidence interval that are outside the x-axis limits. *Per 1000 births;**Restricted to births from 28 weeks onwards*

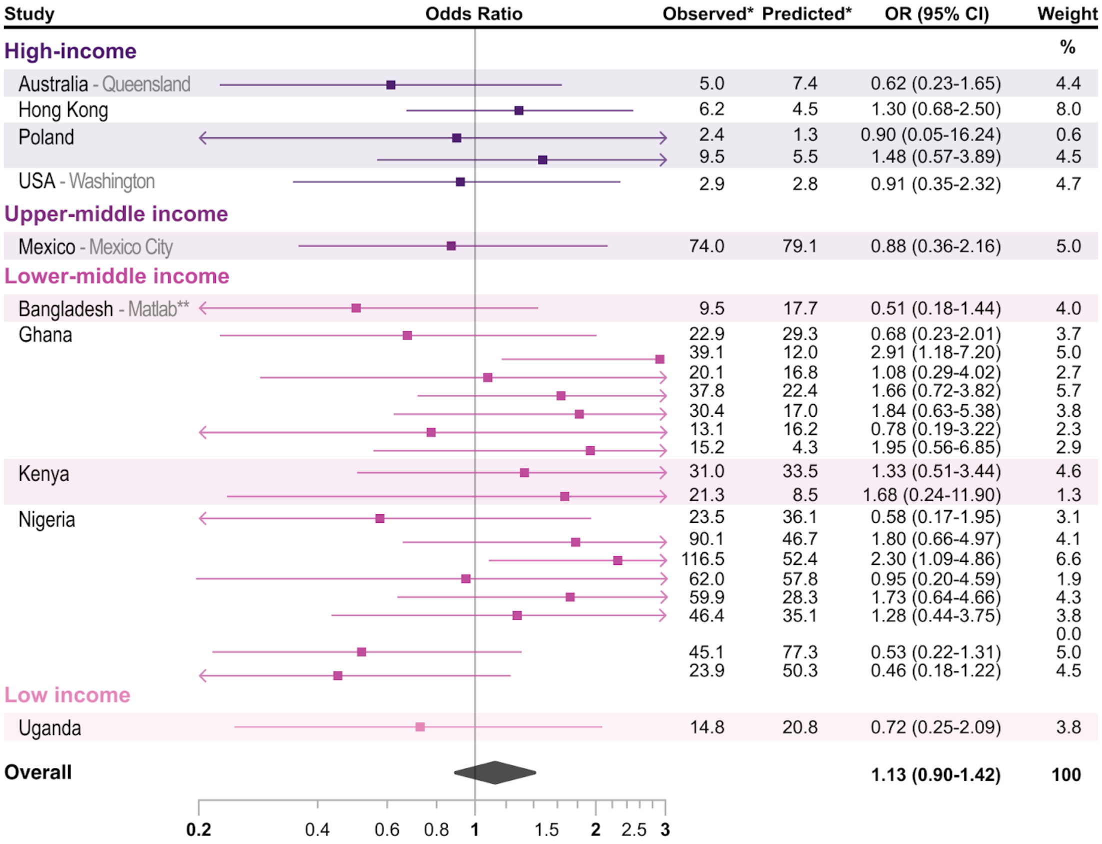

*Supplementary Figure 44: Individual and pooled **non-population-based estimates** of the association between lockdown and the odds of **stillbirth** among all births 22 weeks onwards, in the **fourth month** of lockdown. Individual dataset odds ratios (represented by boxes on plot) were calculated by comparing the observed odds of stillbirth in the fourth month of lockdown to the forecasted odds of stillbirth in the fourth month of lockdown from a linear regression model model that was fitted to pre-lockdown data. Horizontal lines surrounding each box on the forest plot are 95% confidence intervals. Arrows indicate upper and or lower bounds of the confidence interval that are outside the x-axis limits. *Per 1000 births;**Restricted to births from 28 weeks onwards*

Supplementary Tables

Supplementary Table 1: Summary of datasets included in the international Perinatal Outcomes in the Pandemic (iPOP) Study.

World Region Country (Region)	World Bank Income Setting	Data source	Nationwide, regional data, hospital or other data (% of births covered for population-based datasets)	Method/s used in data source to estimate gestational age	Years	Date of first COVID-19 lockdown in 2020 (i.e., Oxford Stringency Index reached 50 or over)*	Oxford Stringency Index at lockdown (max in first lockdown period)*
**Population-based**							
**Asia-Pacific**							
Australia (New South Wales)	High	Perinatal Data Collection (PDC) including all live- and stillbirths	Regional, Statewide (>99%)	Ultrasound, last menstrual period	2015-2020	March 23	52.8 (75.5)
**Middle East & North Africa**							
Iran	Upper-middle	National neonatal data including all live- and stillbirths	National (>95%)	Ultrasound, last menstrual period	2017-2020	March 19	51.9 (59.3)
**Europe**							
Belgium	High	Regional Birth Register including all live- and stillbirths	National (100%)	Ultrasound, last menstrual period	2015-2020	March 14	50.9 (81.5)
Denmark	High	Regional Birth Register including all live- and stillbirths	Regional (98% of births in Central Denmark Region)	Ultrasound, last menstrual period	2016-2020	March 13	63.0 (72.2)
Finland	High	National Medical Birth Register including all live- and stillbirths	National (100%)	Ultrasound, last menstrual period	2015-2020	March 16	61.1 (71.3)
Hungary	High	National Birth Register including all live- and stillbirths	National (100%)	Last menstrual period	2015-2020	March 12	50.0 (76.9)
Iceland	High	National Medical Birth Register including all live- and stillbirths	National (100%)	Ultrasound, last menstrual period	2015-2020	March 16	50.9 (53.7)
Norway	High	National Medical Birth Register including all live- and stillbirths	National (100%)	Ultrasound, last menstrual period	2015-2020	March 15	51.8 (79.6)
Scotland	High	Maternity care discharge records linked to statutory stillbirth records including all live- and	National (99%)	Ultrasound, last menstrual period	2015-2020	March 22	62.0 (79.6)

		stillbirths, but excluding home births					
Sweden	High	Swedish Pregnancy Register including all live- and stillbirths, but excluding planned births outside of hospital, excluding planned home births but including unplanned births outside of hospital	National (94%)	Ultrasound, last menstrual period	2015-2020	March 25	50.9 (64.8)
Switzerland	High	Federal Statistical Office, Vital Statistics (BEVNAT) Switzerland including live- and stillbirths	National (100%)	Ultrasound, last menstrual period, symphysis-fundal height	2015-2020	March 17	73.2 (73.2)
Wales	High	National Community Child Health Database and Maternity Indicators Dataset including all live- and stillbirths	National (100%)	Ultrasound, last menstrual period	2015-2020	March 22	62.0 (79.6)
**North America**							
Canada (excluding Quebec)	High	Canadian Institute for Health Information (CIHI) Discharge Abstract Database (DAD), including live- and stillbirths, but excluding home births	National (98%)	Ultrasound, last menstrual period	2015-2020	March 18	61.1 (74.5)
USA	High	Data extracted from birth certificates, which are required to be completed for all births	National (>99%)	Ultrasound, last menstrual period	2015-2020	March 16	52.3 (72.7)
**Latin America & Caribbean**							
Brazil	Upper-middle	National Birth Register including all live- and stillbirths	National (100%)	Last menstrual period	2015-2020	March 17	57.9 (81.0)
Chile	High	National Birth Registry including all live births	National (100%)	Ultrasound, last menstrual period	2015-2020	March 18	55.6 (87.5)
Peru	Upper-middle	National Birth Registry including all live births	National (100%)	Ultrasound, last menstrual period, ballard score, other	2016-2020	March 14	50.0 (96.3)
Uruguay	High	National birth register including live- and stillbirths	National (>99%)	Last menstrual period, ballard score	2015-2020	March 15	51.9 (72.2)
**Non-population-based**							
**Asia-Pacific**							
Australia (Queensland)	High	Antenatal data record from a tertiary facility including live- and stillbirths, including home births	One hospital	Ultrasound, last menstrual period	2015-2020	March 23	52.8 (75.5)
Hong Kong	High	Clinical Data Analysis and Reporting System (CDARS) including all live- and stillbirths from all public sector health facilities	Facility (80% of all births in Hong Kong, excludes private sector)	Ultrasound, last menstrual period	2015-2020	February 8	52.8 (66.7)

**South Asia**							
Bangladesh	Lower-middle	Data from Matlab Health and Demographic Surveillance System (HDSS) including live- and stillbirths at both home and in facilities	Demographic surveillance system (coverage 90% of study area)	Ultrasound, last menstrual period	2015-2020	March 19	75.9 (93.5)
**Europe**							
Poland	High	Hospital medical records including live- and stillbirths	Two hospitals	Ultrasound, last menstrual period	2015-2020	March 15	57.4 (87.0)
**North America**							
USA (Washington State)	High	Obstetrical Care Outcomes Assessment Program, maternal and neonatal medical records	14 hospitals	Ultrasound, last menstrual period	2017-2020	March 16	52.3 (72.7)
**Latin America & Caribbean**							
Mexico	Upper-middle	Medical records from tertiary facility, hospital maternity and labour ward records including live- and stillbirths	One hospital	Ultrasound, last menstrual period	2017-2020	March 24	52.8 (82.4)
**Sub-Saharan Africa**							
Ghana	Lower-middle	Paper-based births registers including live- and stillbirths	Seven hospitals	Ultrasound, last menstrual period, symphysis-fundal height	1 hospital: 2015-2020 6 hospitals: 2017-2020	March 18	50.0 (86.1)
Kenya	Lower-middle	Hospital birth registry including live- and stillbirths	Two hospitals	Ultrasound, last menstrual period	2015-2020	March 15	50.9 (88.9)
Nigeria	Lower-middle	Hospital birth registry including live- and stillbirths	Four hospitals	Ultrasound, last menstrual period, symphysis-fundal height	2015-2020	March 26	52.3 (85.6)
		Hospital birth registry including live- and stillbirths	Three hospitals	Last menstrual period, Ultrasound dating	2015-2020		
		Hospital birth registry including live- and stillbirths	Two hospitals	Last menstrual period, Ultrasound dating	2015-2020		
Uganda	Low	Hospital birth registry including live- and stillbirths	One hospital	Ultrasound, last menstrual period, ballard score	2015-2020	March 25	69.4 (93.5)

*From Oxford COVID-19 Government Response Tracker: https://www.bsg.ox.ac.uk/research/research-projects/covid-19-government-response-tracker

Supplementary Table 2: Datasets excluded from the study analysis and reasons why

Country	Dataset and type	Reason for exclusion
**Nepal**	All, seven facilities	Data started from January 2018, and in most facilities there was a data quality exercise conducted in early 2019 inflating preterm birth rates during this period, making it impossible to draw inferences about impact of lockdown in 2020 (see Supplementary Figure 1).
**Ghana**	Facility 1	Small numbers of births (<50 per month).
	Facility 8 & 10	No available data for 2015-2019, only 2020.
**Nigeria**	Facility 5 & 6	No available data on gestational age, only birth weight. These are peripheral facilities which generally do not collect data on gestational age.
	Facility 7	While this is the largest private facility conducting private deliveries in the state, there were relatively small number of births (<50 per month).
**Kenya**	Facility 2	No data available from April 2020 onwards.
**Uganda**	Facility 1	Seven months of data missing in 2019.

Sensitivity analysis: comparison of change in association between lockdown and preterm births rates when using all births versus live births only

Supplementary Table 3: Odds ratio for change in preterm birth rates (births from 22 weeks onwards) with lockdown calculated by using [1] all births and [2] live births only for all population-based datasets

Country	First month of lockdown	Second month of lockdown	Third month of lockdown	Fourth month of lockdown
**Australia, NSW***				
All births	0.92 (0.83-1.03)	1.02 (0.91-1.13)	0.97 (0.87-1.08)	-
Live births	0.90 (0.81-1.01)	1.01 (0.91-1.13)	0.97 (0.87-1.08)	-
**Belgium**				
All births	0.88 (0.79-0.98)	0.93 (0.83-1.04)	0.96 (0.86-1.07)	1.06 (0.96-1.17)
Live births	0.89 (0.79-0.99)	0.93 (0.83-1.03)	0.97 (0.87-1.08)	1.06 (0.95-1.17)
**Brazil**				
All births	1.00 (0.96-1.04)	1.04 (0.99-1.08)	1.04 (0.99-1.08)	1.03 (0.99-1.08)
Live births	1.00 (0.95-1.04)	1.03 (0.99-1.08)	1.03 (0.99-1.08)	1.03 (0.98-1.07)
**Canada**				
All births	0.95 (0.89-1.02)	0.98 (0.92-1.05)	0.95 (0.89-1.02)	1.02 (0.96-1.08)
Live births	0.97 (0.91-1.04)	1.01 (0.95-1.08)	0.97 (0.91-1.04)	1.04 (0.98-1.11)
**Denmark, Central Region**				
All births	1.01 (0.74-1.37)	1.03 (0.77-1.40)	1.16 (0.87-1.55)	0.89 (0.66-1.20)
Live births	1.01 (0.74-1.37)	1.04 (0.77-1.40)	1.16 (0.87-1.54)	0.89 (0.67-1.20)
**Finland**				
All births	1.06 (0.89-1.26)	1.04 (0.87-1.23)	1.07 (0.90-1.28)	1.02 (0.86-1.21)
Live births	1.06 (0.89-1.27)	1.03 (0.90-1.18)	1.06 (0.89-1.26)	1.01 (0.85-1.20)
**Hungary**				
All births	1.00 (0.87-1.14)	1.02 (0.90-1.17)	0.98 (0.87-1.12)	1.01 (0.90-1.14)
Live births	0.99 (0.86-1.13)	1.03 (0.90-1.18)	0.99 (0.86-1.13)	1.03 (0.90-1.16)
**Iceland**				
All births	1.24 (0.71-2.16)	0.75 (0.41-1.35)	0.71 (0.39-1.30)	0.88 (0.49-1.59)
Live births	1.25 (0.72-2.19)	0.66 (0.35-1.25)	0.62 (0.32-1.18)	0.89 (0.49-1.63)
**Iran**				
All births	0.87 (0.78-0.98)	0.92 (0.82-1.03)	0.97 (0.87-1.09)	1.00 (0.90-1.12)
Live births	0.86 (0.76-0.97)	0.91 (0.81-1.02)	0.96 (0.86-1.08)	1.00 (0.90-1.11)
**Norway**				
All births	1.01 (0.88-1.16)	0.99 (0.86-1.15)	0.84 (0.72-0.97)	0.97 (0.84-1.12)
Live births	1.02 (0.89-1.18)	0.99 (0.85-1.15)	0.83 (0.71-0.97)	0.98 (0.85-1.14)
**Scotland**				
All births	0.91 (0.79-1.05)	0.86 (0.75-1.00)	1.05 (0.92-1.20)	0.96 (0.83-1.10)
Live births	0.89 (0.77-1.03)	0.85 (0.74-0.99)	1.05 (0.91-1.20)	0.95 (0.82-1.09)
**Sweden**				
All births	1.01 (0.90-1.13)	0.94 (0.84-1.05)	0.98 (0.87-1.10)	-
Live births	1.01 (0.90-1.13)	0.95 (0.85-1.07)	0.97 (0.86-1.09)	-
**Switzerland**				
All births	0.98 (0.86-1.10)	0.90 (0.80-1.02)	1.04 (0.93-1.17)	1.08 (0.96-1.22)
Live births	0.97 (0.86-1.08)	0.90 (0.80-1.01)	1.05 (0.94-1.17)	1.09 (0.97-1.22)
**Uruguay**				
All births	0.95 (0.80-1.13)	1.00 (0.84-1.19)	1.03 (0.87-1.23)	0.90 (0.76-1.08)
Live births	0.98 (0.82-1.12)	1.04 (0.87-1.24)	1.06 (0.88-1.26)	0.94 (0.78-1.12)
**Wales***				
All births	0.95 (0.81-1.11)	0.89 (0.75-1.05)	0.96 (0.83-1.12)	1.03 (0.88-1.20)
Live births	0.96 (0.82-1.12)	0.89 (0.75-1.05)	0.96 (0.83-1.12)	1.04 (0.89-1.22)

*Births 24 weeks onwards; NSW=New South Wales

Sensitivity analysis: comparison of change in association between lockdown and preterm births rates when restricting to births 28 weeks onwards

Supplementary Table 4: Odds ratio for change in preterm birth rates with lockdown calculated by using [1] all births 22 weeks onwards and [2] all births from 28 weeks onwards, for all population-based datasets

Country	First month of lockdown	Second month of lockdown	Third month of lockdown	Fourth month of lockdown
**Belgium**				
22 weeks	0.88 (0.79-0.98)	0.93 (0.83-1.04)	0.96 (0.86-1.07)	1.06 (0.96-1.17)
28 weeks	0.90 (0.81-0.99)	0.95 (0.86-1.05)	0.96 (0.87-1.06)	1.06 (0.96-1.16)
**Brazil**				
22 weeks	1.00 (0.96-1.04)	1.04 (0.99-1.08)	1.04 (0.99-1.08)	1.03 (0.99-1.08)
28 weeks	1.00 (0.96-1.05)	1.04 (0.99-1.08)	1.04 (1.00-1.09)	1.03 (0.98-1.08)
**Canada**				
22 weeks	0.95 (0.89-1.02)	0.98 (0.92-1.05)	0.95 (0.89-1.02)	1.02 (0.96-1.08)
28 weeks	0.95 (0.95-1.02)	0.99 (0.93-1.06)	0.97 (0.91-1.03)	1.02 (0.96-1.08)
**Denmark, Central Region**				
22 weeks	1.01 (0.74-1.37)	1.03 (0.77-1.40)	1.16 (0.87-1.55)	0.89 (0.66-1.20)
28 weeks	1.01 (0.73-1.40)	1.02 (0.75-1.40)	1.04 (0.76-1.41)	0.81 (0.59-1.11)
**Finland**				
22 weeks	1.06 (0.89-1.26)	1.04 (0.87-1.23)	1.07 (0.90-1.28)	1.02 (0.86-1.21)
28 weeks	1.05 (0.88-1.26)	1.01 (0.85-1.20)	1.05 (0.88-1.26)	1.00 (0.84-1.18)
**Hungary**				
22 weeks	1.00 (0.87-1.14)	1.02 (0.90-1.17)	0.98 (0.87-1.12)	1.01 (0.90-1.14)
28 weeks	1.00 (0.87-1.14)	1.01 (0.88-1.15)	0.99 (0.87-1.13)	1.01 (0.89-1.14)
**Iceland**				
22 weeks	1.24 (0.71-2.16)	0.75 (0.41-1.35)	0.71 (0.39-1.30)	0.88 (0.49-1.59)
28 weeks	1.16 (0.64-2.08)	0.67 (0.35-1.27)	0.67 (0.35-1.28)	0.85 (0.45-1.58)
**Iran**				
22 weeks	0.87 (0.78-0.98)	0.92 (0.82-1.03)	0.97 (0.87-1.09)	1.00 (0.90-1.12)
28 weeks	0.87 (0.77-0.98)	0.91 (0.82-1.02)	0.97 (0.86-1.08)	1.01 (0.91-1.12)
**Norway**				
22 weeks	1.01 (0.88-1.16)	0.99 (0.86-1.15)	0.84 (0.72-0.97)	0.97 (0.84-1.12)
28 weeks	1.01 (0.87-1.18)	0.99 (0.85-1.16)	0.84 (0.72-0.99)	0.95 (0.81-1.11)
**Scotland**				
22 weeks	0.91 (0.79-1.05)	0.86 (0.75-1.00)	1.05 (0.92-1.20)	0.96 (0.83-1.10)
28 weeks	0.91 (0.79-1.05)	0.85 (0.73-0.98)	1.05 (0.91-1.20)	0.96 (0.83-1.10)
**Sweden**				
22 weeks	1.01 (0.90-1.13)	0.94 (0.84-1.05)	0.98 (0.87-1.10)	-
28 weeks	1.03 (0.92-1.15)	0.93 (0.83-1.04)	0.97 (0.87-1.09)	-
**Switzerland**				
22 weeks	0.98 (0.86-1.10)	0.90 (0.80-1.02)	1.04 (0.93-1.17)	1.08 (0.96-1.22)
28 weeks	0.99 (0.87-1.12)	0.90 (0.79-1.02)	1.04 (0.92-1.17)	1.09 (0.97-1.23)
**Uruguay**				
22 weeks	0.95 (0.80-1.13)	1.00 (0.84-1.19)	1.03 (0.87-1.23)	0.90 (0.76-1.08)
28 weeks	0.95 (0.79-1.14)	0.99 (0.82-1.19)	1.05 (0.88-1.26)	0.90 (0.75-1.09)
**Wales***				
24 weeks	0.95 (0.81-1.11)	0.89 (0.75-1.05)	0.96 (0.83-1.12)	1.03 (0.88-1.20)
28 weeks	0.93 (0.78-1.10)	0.88 (0.73-1.05)	0.98 (0.83-1.15)	1.01 (0.85-1.20)

*Births 24 weeks onwards

Sensitivity analysis: comparison of change in association between lockdown and preterm births rates in the meta-analysis when removing large countries

Supplementary Table 5: Pooled population-based estimates of the association between lockdown and the odds of preterm birth among all births 22 weeks onwards by month of lockdown, with estimates presented for our primary analysis (including all population-based datasets) as well as the sensitivity analysis excluding data from Brazil and the USA

Month of lockdown	Primary analysis (including all countries)		Sensitivity analysis (excluding data from Brazil and the USA)	
	Number of studies	Pooled odds ratio (95% CI)	Number of studies	Pooled odds ratio (95% CI)
First month	18	0.96 (0.95-0.98)	16	0.96 (0.93-0.98)
Second month	18	0.96 (0.92-0.99)	16	0.95 (0.91-0.99)
Third month	18	0.97 (0.94-1.00)	16	0.96 (0.93-1.00)
Fourth month	16	0.99 (0.96-1.01)	14	0.98 (0.95-1.02)

*CI=Confidence interval

Supplementary Table 6: Details of ethical approval

Country, Region/Site	Ethical Approval Required	Further details
**Population-based data**		
**Asia-Pacific**		
**Australia, New South Wales**	Yes	Use of aggregated data for this study was approved by the NSW Population and Health Services Research Ethics Committee (2019/ETH11532)
**Middle East & North Africa**		
**Iran**	Yes	National approval code: IR.MUI.REC.1400.043.
**Europe**		
**Belgium**	Yes	The Ethics committee of the hospital AZ St-Jan Bruges was informed and responded to us on 16/02/2021 the following: *"The Ethics committee has received the documentation of the aforementioned trial. We have no objections for this retrospective non-interventional study to be performed".*
**Denmark, Central Region**	Yes	Permission to data access was obtained from the Regional Council of the Central Denmark Region (§46 permission), sagnr. 1-45-70-43-20, 6 Jan 2021.Data protection (GDPR) sagsnr 1-16-02-611-20 and permission to share anonymised data was obtained 26 Jan 2021.
**Finland**	No	Only aggregated data provided, no need for ethical approval
**Hungary**	No	Only aggregated data provided, no need for ethical approval
**Iceland**	Yes	Ethical approval was obtained from the National Bioethics Committee on Oct 13th, 2020. VSNb2020080003/03.0 I
**Norway**	No	Only aggregated data provided, no need for ethical approval
**Scotland**	Yes	Ethical approval per se was not required, but approval for contribution of Scottish data was secured from the Public Health Scotland Data Protection team
**Sweden**	No	Only aggregated data provided, no need for ethical approval
**Switzerland**	No	Only aggregated data provided, no need for ethical approval
**Wales**	No	Data provided through SAIL.
**North America**		
**Canada**	No	Only aggregated data provided from publicly available data, no need for ethical approval
**USA**	No	Only aggregated data provided from publicly available data, no need for ethical approval
**Latin America & Caribbean**		
**Brazil**	No	Only aggregated data provided from publicly available data, no need for ethical approval
**Chile**	No	No ethical approval needed given that public access databases of the Civil Registry Service (for 2019 and 2020) and the Department of Statistics of the Ministry of Health were accessed from 2015 to 2018
**Peru**	No	Only aggregated data provided, no need for ethical approval. However, the study protocol was approved by the Institutional Review Board of Universidad Peruana Cayetano Heredia (Reference Number: CONSTANCIA 101-01-21)
**Uruguay**	No	Only aggregated data provided from publicly available data, no need for ethical approval
**Non-population-based data**		
**Asia-Pacific**		
**Hong Kong**	Yes	The study protocol was approved by the Institutional Review Board of the University of Hong Kong/ Hospital Authority Hong Kong West Cluster for CDARS database research (Reference Number: UW 20-166)
**Australia, Queensland**	No	Did not meet requirements for HREC review and is considered a clinical audit. EXMT/MML/73974 (V1)
**South Asia**		
**Matlab, Bangladesh**	Yes	Ethical approval was received by the IRB of International Centre for diarrheal Disease Research, Bangladesh (icddr,b)

**Europe**		
**Poland**		
Poznań University of Medical Sciences	No	Only aggregated data provided, no need for ethical approval. Waiver was obtained from the Poznań University of Medical Sciences Ethical Review Board.
Poznań Regional Hospital	No	Only aggregated data provided, no need for ethical approval.
**North America**		
**Washington state, USA**	No	Research did not include human subjects, IRB review was not required.
**Latin America & Caribbean**		
**Mexico City, Mexico**	Yes	Ethical approval was obtained from the IRB of the National Institute of Perinatology on May 4th, 2021 (2021-1-21).
**Sub-Saharan Africa**		
**Ghana**	Yes	The study was approved by the Ghana Health Service Ethics Review Committee (No. GHS-ERC 006/03/21).
**Kenya**	Yes	The study was approved by Jomo Kenyatta University of Agriculture and Technology Institutional Ethics Review Committee.
**Nigeria**		
Ibadan	Yes	The protocol was approved by University of Ibadan/University College Hospital Ibadan Ethics Review Committee UI/EC/21/0107.
Jos University	Yes	Ethical approval was obtained from Jos University Teaching Hospital, Bingham University Teaching Hospital Jos, and Plateau State Specialist Hospital.
Uyo Teaching Hospital	Yes	Ethical approval was obtained from the University of Uyo Teaching Hospital Ethical Review Board, Reference number UUTH/AD/96/Vol XXI/522. Permission was also obtained from the Akwa Ibom State Ministry of Health.
**Uganda**	No	No ethical approval needed. We obtained administrative clearance and reviewed records from the Department of Obstetrics and Gynaecology.

Supplementary References

1. Ford, D. V. *et al.* The SAIL Databank: building a national architecture for e-health research and evaluation. *BMC Health Serv. Res.* **9**, 157 (2009).

2. Jones, K. H. *et al.* A case study of the Secure Anonymous Information Linkage (SAIL) Gateway: A privacy-protecting remote access system for health-related research and evaluation. *J. Biomed. Inform.* **50**, 196–204 (2014).

3. Welpton, R. *SDC Handbook*. (figshare, 2019).

4. Kostenzer, J. et al. Neonatal care during the COVID-19 pandemic - a global survey of parents' experiences regarding infant and family-centred developmental care. EClinicalMedicine 39, 101056 (2021).
