# Supplementary material for: Changes in preterm birth and stillbirth during COVID-19 lockdowns in 26 countries
Source: Nat Hum Behav. 2023 Feb 27;7(4):529–44. doi: 10.1038/s41562-023-01522-y (PMC10129868; doi:10.1038/s41562-023-01522-y)
Supplement: Supplementary file 2 — Reporting Summary [file 41562_2023_1522_MOESM2_ESM.pdf]

nature portfolio

Corresponding author(s): Meghan Azad
Sarah Stock
David Burgner

Last updated by author(s): Oct 19, 2022

Reporting Summary

Nature Portfolio wishes to improve the reproducibility of the work that we publish. This form provides structure for consistency and transparency in reporting. For further information on Nature Portfolio policies, see our Editorial Policies and the Editorial Policy Checklist.

Statistics

For all statistical analyses, confirm that the following items are present in the figure legend, table legend, main text, or Methods section.

n/a	Confirmed	
☐	☒	The exact sample size (*n*) for each experimental group/condition, given as a discrete number and unit of measurement
☒	☐	A statement on whether measurements were taken from distinct samples or whether the same sample was measured repeatedly
☐	☒	The statistical test(s) used AND whether they are one- or two-sided<br>*Only common tests should be described solely by name; describe more complex techniques in the Methods section.*
☐	☒	A description of all covariates tested
☐	☒	A description of any assumptions or corrections, such as tests of normality and adjustment for multiple comparisons
☐	☒	A full description of the statistical parameters including central tendency (e.g. means) or other basic estimates (e.g. regression coefficient) AND variation (e.g. standard deviation) or associated estimates of uncertainty (e.g. confidence intervals)
☐	☒	For null hypothesis testing, the test statistic (e.g. *F*, *t*, *r*) with confidence intervals, effect sizes, degrees of freedom and *P* value noted<br>*Give P values as exact values whenever suitable.*
☒	☐	For Bayesian analysis, information on the choice of priors and Markov chain Monte Carlo settings
☒	☐	For hierarchical and complex designs, identification of the appropriate level for tests and full reporting of outcomes
☒	☐	Estimates of effect sizes (e.g. Cohen's *d*, Pearson's *r*), indicating how they were calculated

Our web collection on statistics for biologists contains articles on many of the points above.

Software and code

Policy information about availability of computer code

Data collection	This study was a secondary analysis of anonymized data so no data collection software were used.
Data analysis	All analyses were performed in R version 4.1.1. Analysis code is available on request from Sarah Stock (sarah.stock@ed.ac.uk).

For manuscripts utilizing custom algorithms or software that are central to the research but not yet described in published literature, software must be made available to editors and reviewers. We strongly encourage code deposition in a community repository (e.g. GitHub). See the Nature Portfolio guidelines for submitting code & software for further information.

Data

Policy information about availability of data

All manuscripts must include a data availability statement. This statement should provide the following information, where applicable:

- Accession codes, unique identifiers, or web links for publicly available datasets
- A description of any restrictions on data availability
- For clinical datasets or third party data, please ensure that the statement adheres to our policy

This study makes use of anonymized data held in the Secure Anonymized Information Linkage (SAIL) Databank. We would like to acknowledge all the data providers who made anonymized data available for research (listed in Supplementary Table 1). The responsibility for the interpretation of the information SAIL supplied is the authors' alone. Data may be available to researchers for analysis after securing relevant permissions from the data contributors and the databank in which the data are held (SAIL Databank). The approvals process is managed by application to the SAIL Databank who hold data sharing agreements with the data providers.

Restricted datasets may require additional approvals from data custodians and ethical authorities in the relevant country/setting. Enquiries for data access should be made using the contact form at https://saildatabank.com/contact, or by making an enquiry to ICODA at https://icoda-research.org/contact/.

Human research participants

Policy information about studies involving human research participants and Sex and Gender in Research.

Reporting on sex and gender	We do not look at sex/gender in this paper.
Population characteristics	This study was a secondary analysis utilizing aggregate data on the monthly number of births broken down by gestational age at birth groups. There was further disaggregation available in some datasets by whether they were live births or stillbirths and, specifically for preterm births (i.e., births at <37 weeks gestation), by whether they were spontaneous preterm births. There were no individual level data available in these datasets.
Recruitment	This was a secondary data analysis so there was no recruitment undertaken as part of the study.
Ethics oversight	Contributors from countries where the data were not publicly available obtained ethics approval from their respective institutional review boards (Supplementary Table 6). We did not seek ethical approval for publicly available data sources (Supplementary Table 6). All data contributors completed a Data Completion Agreement, which outlined the terms and conditions for uploading and storing data to the SAIL databank.

Note that full information on the approval of the study protocol must also be provided in the manuscript.

Field-specific reporting

Please select the one below that is the best fit for your research. If you are not sure, read the appropriate sections before making your selection.

☒ Life sciences ☐ Behavioural & social sciences ☐ Ecological, evolutionary & environmental sciences

For a reference copy of the document with all sections, see nature.com/documents/nr-reporting-summary-flat.pdf

Life sciences study design

All studies must disclose on these points even when the disclosure is negative.

Sample size	We ultimately included all eligible datasets in this analysis, with the sample size determined by this. For preterm birth, we are able to detect small changes with our sample size, particularly when we pool across all the datasets, so we are confident we have a sufficient sample size for this outcome. For stillbirth, which is a rarer outcome, there is greater uncertainty in our estimates (acknowledged in the discussion), but we still have sufficient power to detect associations for some countries.
Data exclusions	We excluded datasets if: (1) there were a small number of monthly births (<50) or (2) there was an insufficient timespan of data to reliably predict the expected preterm birth rates in the lockdown period or (2) there were implausible preterm birth rates in the dataset.
Replication	We undertook at number of sensitivity analyses varying our study population (for example, restricting to only live births), and examined the impact of removing large countries from the meta-analysis, none of which changed our conclusions.
Randomization	It would not be possible to randomize pregnant women to be exposed to pandemic-related restrictions ("lockdown") or not, so we instead relied on available aggregate level data to conduct this observational analysis. As with most analyses of aggregate level data, we were also unable to control for individual-level confounders that may affect the association between lockdown and our perinatal outcomes, as outlined in the limitations section in the Discussion.
Blinding	This study relied on aggregate level data extract from health facility records, electronic routine health records or national reporting systems. As all eligible births were included in the dataset and analysis, no blinding was required.

Reporting for specific materials, systems and methods

We require information from authors about some types of materials, experimental systems and methods used in many studies. Here, indicate whether each material, system or method listed is relevant to your study. If you are not sure if a list item applies to your research, read the appropriate section before selecting a response.

Materials & experimental systems

n/a	Involved in the study
☒	☐ Antibodies
☒	☐ Eukaryotic cell lines
☒	☐ Palaeontology and archaeology
☒	☐ Animals and other organisms
☒	☐ Clinical data
☒	☐ Dual use research of concern

Methods

n/a	Involved in the study
☒	☐ ChIP-seq
☒	☐ Flow cytometry
☒	☐ MRI-based neuroimaging
